# Sacha Inchi (*Plukenetia volubilis* L.) Protein Hydrolysate as a New Ingredient of Functional Foods

**DOI:** 10.3390/foods13132045

**Published:** 2024-06-27

**Authors:** Ana Lemus-Conejo, Alvaro Villanueva-Lazo, Maria E. Martin, Francisco Millan, Maria C. Millan-Linares

**Affiliations:** 1 Foundation Centre for Research and Development of Functional Food—CIDAF, Avda del Conocimiento 37, 18100 Granade, Spain; alemus@us.es; 2 Food Protein and Immunonutrition Group, Department of Food and Health, Instituto de la Grasa, CSIC, Campus Universitario Pablo de Olavide, Edificio 46, Ctra. de Utrera, Km. 1, 41013 Seville, Spain; fmillanr@ig.csic.es (F.M.); mcmillan@ig.csic.es (M.C.M.-L.); 3 Department of Cell Biology, Faculty of Biology, University of Seville, Av. Reina Mercedes s/n, 41012 Seville, Spain; mariamartin@us.es

**Keywords:** Sacha inchi, bioactive peptides, protein hydrolysates, THP-1 cells, in silico analysis

## Abstract

Sacha inchi (*Plukenetia volubilis* L.) is an under-exploited crop with great potential due to its nutritional and medicinal characteristics. A Sacha inchi protein isolate (SII), obtained from defatted Sacha inchi flour (SIF), was hydrolyzed by Bioprotease LA 660 under specific conditions. The hydrolysates were characterized chemically, and their digestibility and antioxidant capacity were evaluated by in vitro cell-free experiments to select the hydrolysate with major antioxidant activity. Sacha inchi protein hydrolysate at 20 min (SIH20B) was selected, and the anti-inflammatory capacity was evaluated by RT-qPCR and ELISA techniques, using two different doses in monocytes THP-1 stimulated with lipopolysaccharide (LPS). The results obtained showed that the in vitro administration of SIH20B down-regulated the TNF-α gene and reduced the release of this cytokine, whereas the anti-inflammatory cytokines IL-10 and IL-4 were up-regulated in LPS-stimulated monocytes and co-administrated with SIH20B. The peptides contained in SIH20B were identified, and the 20 more relatively abundant peptides with a mass by 1 kDa were subjected to in silico analysis to hypothesize those that could be responsible for the bioactivity reported in the hydrolysate. From the identified peptides, the peptides AAGALKKFL and LGVKFKGGL, among others, are proposed as the most biologically actives. In conclusion, SIH20B is a novel, natural source of high-value-added biopeptides that could be used as an ingredient in formulations of food or nutraceutical compounds.

## 1. Introduction

Today’s world is facing a great paradox: a large part of the population is dying of starvation and another large part, well nourished, is dying of chronic non-communicable diseases, including cardiovascular diseases, diabetes, cancer, and chronic respiratory diseases, among others. On the other hand, climate change, population growth, and land and environmental degradation mean that food production systems need to be fundamentally reformed. Thus, both the need for food and for it to be healthy has led international organizations, governments, industries, and consumers to demand new nutrient-rich, calorie-efficient, healthy, and environmentally sustainable foods, like new sources of protein such as legumes; algae; insects; jellyfish, microbial proteins; or vegetable seeds of underutilized and undervalued plant species [1], such as quinoa, flax, amaranth, hemp, chia, or Sacha inchi. Underutilized crops have a large untapped potential and can make a contribution, among other things, to food security, including improved nutrition and health through diet component diversification; enhance income opportunities; and serve as risk management tools for smallholder farmers [2,3].

Sacha inchi (*Plukenetia volubilis* L.), commonly known as Inca inchi, Inca peanut, or wild peanut mountain peanut, is native to the Amazon rainforests and is cultivated in Colombia and Ecuador, as well as in some Southeast Asian countries, such as Thailand, Vietnam, and China [4]. It grows in warm climates, at altitudes ranging from 200 and 1500 m above sea level [5,6,7]. This plant belongs to the Euphorbiaceae family, which is composed of 19 species [1], and belongs to the Plukenetia genus, which includes *P. brachybotrya*, *P. polyadenia*, *P. loretensis*, and *P. huayllabambana* [8]. It is an oleaginous, deciduous, vine-like, or shrub-like plant reaching a height of up to 2 m. From the age of 1 year, it produces lobed green fruits with 4–7 carpellate protected by a hard outer shell with a thin layer of soft tissue inside, which, once matured, contains 1.5–2 cm dark brown oval seeds [1,7]. Since the seeds are bitter, it is usually consumed after proper processing (e.g., roasting), and the leaves are also cooked for consumption [9,10].

Sacha inchi seeds are currently appearing as a potential source of macro- and micronutrients and bioactive compounds [11,12], with likely applications in the food and pharmaceutical industry [13]. The oil content ranges from 35 to 60%, according to different authors, including polyunsaturated fatty acids (PUFA) such as α-linolenic acid (ALA; C18:3, ω-3; 47–51%) and linoleic acid or (LA; C18:2, ω-6; 34–37%), in addition to monounsaturated fatty acids like oleic acid (~9.5%) and saturated fatty acids such as palmitic (4.4%) or stearic (2.7%) [12]. The seed protein content varies between 25 and 33% depending on the extraction method and cultivar [13]. The carbohydrate content varies from 12.1% to 30.9%, according to various authors [14,15,16]. Other minor compounds of note in Sacha inchi seed are vitamin E in α-tocopherol (50–114 mg/g oil) and δ-tocopherol (30–125 mg/g) forms, phenols, campesterol, stigmasterol, β-sitosterol, flavonoids, carotenoids, secoiridoids, lignans, and minerals [6].

The part of the population that suffers from chronic non-communicable diseases demands foods that not only nourish but also improve health and decrease the risk of contracting certain diseases; that is to say, they demand so-called functional foods, which are foods containing substances or compounds with a specific biological activity, commonly called bioactives, which can be of different natures and contain different pre- and pro-biotics, polyphenols, carotenoids, dietary fiber, sterols, vitamins, fatty acids, peptides, etc. [17]. Peptides can be generated by enzymatic hydrolysis, a fast, safe, and easily controllable method of producing protein hydrolysates [18], which are defined as a mixture of small peptides or even free amino acids that are produced by chemically or biologically methods and can contain bioactive peptides with a size between 2 and 20 amino acid units [19]. Bioactive peptides and hydrolyzed proteins, unlike the original proteins, usually have low-molecular-weight structures, thus enhancing digestibility and bioavailability [20,21].

Inflammation is a defense mechanism of the body to eliminate or limit the spread of harmful stimuli and heal the injury. Excessive inflammation is associated with the development of multiple diseases, such as inflammatory bowel disease, atherosclerosis, rheumatoid arthritis, Alzheimer’s disease, and even cancer [22]. It is a complex process modulated by a number of inflammatory mediators and cytokines and involves a long chain of molecular reactions and cellular activity [23]. THP-1 is a human monocyte-derived cell line [24] and it is widely used to study anti-inflammatory effects from different components [25,26]. During inflammatory processes, macrophages and inflammatory cells release inflammatory mediators such as cytokines [27], which are easily measurable biomarkers of inflammation, including tumor necrosis factor-α (TNF-α), interleukin-1β (IL-1β), or IL-6 [28].

The intake of antioxidant-rich foods may reduce the adverse effects of oxidative stress. Plant-based diets, due to the antioxidant, immunomodulatory, and anti-inflammatory properties of their bioactive compounds, may reduce the development and progression of disease. Convincing evidence coming from intervention studies demonstrates that plant-based diets modulate immune and inflammatory processes in metabolically afflicted patients via decreases in biomarkers of inflammatory status concentrations, like C-reactive protein (CRP) and IL-6 [29]. One possible inflammatory mechanism involved in the recognition of pathogens such as LPS or toxins (DAMS) involves the TLR4 receptor altering the metabolism of immune cells and releasing a large number of mediators, leading to the activation of nuclear factor κppa B (NF-κB) and TNFα [30]. Recent research on kiwicha protein hydrolysates recognizes another possible mechanism through the NLRP3 (receptor), an intracellular sensor which detects a wide range of microbial motifs, endogenous danger signals, and environmental irritants, leading to the formation and activation of the inflammasome, an essential process in natural immune defense mechanisms, such as chronic inflammation [31]. In addition, several in vitro studies using cells or animals have shown that immunomodulatory peptides from various food proteins, including plant sources, that are generated by enzymatic hydrolysis can be used as potential functional food ingredients with immunomodulatory activity and effects on neurological parameters, blood pressure, glucose homeostasis, and blood lipids or gut microbiota, among other things [32].

Thus, this search for safe, healthy, and sustainable food ingredients has led, in recent years, to a growing interest in seeds of ancestral crops such as Sacha inchi, which is presented as an alternative source of quality protein in view of its high nutritional value and healthy properties. The aim of this study was to obtain and optimize Sacha inchi (*Plukenetia volubilis* L.) seed protein hydrolysates from Sacha inchi protein isolate that can be used as potential ingredients in functional foods. We performed this, firstly, through their proximal composition, degree of hydrolysis, digestibility, amino acid composition, and molecular profile; and, secondly, through cell-free “in vitro” and cell-free “in vitro” to support their antioxidant and anti-inflammatory potential in the prevention and treatment of chronic diseases. The hydrolysate with the highest antioxidant activity was selected—the one hydrolyzed with Bioprotease and inactivated after 20 min (SIH20B)—and its anti-inflammatory activity was studied in THP-1 cells.

## 2. Materials and Methods

### 2.1. Material and Reagents

Sacha inchi was obtained from Inkanat. Sacha inchi protein isolate (SII) was achieved at a pilot scale from 20 kg defatted Sacha inchi flour (SIF), as described by Millan-Linares et al. [33], with minor modifications. Briefly, the extraction ratio was adjusted to 1:10 (*w*/*v*) during 1 h, at ambient temperature and a controlled pH, under continuous stirring. Following centrifugation, the supernatant was collected and adjusted to pH 4.5, which is the isoelectric point of Sacha inchi proteins, before being centrifuged again. Lastly, the proteins precipitated were dried using a co-current spray dryer (Production Minor^TM^; (GEA Niro, Søborg, Denmark) fitted with a rotary wheel (0.1 m diameter) atomizer, at an inlet temperature of 190 °C, outlet temperature of 90 °C, and air volume flow of 250 m^3^/h, and stored at room temperature. Bioprotease LA-660 was purchased from BIOCON (Barcelona, Spain). Chemicals compounds, solvents, and reagents were provided by Sigma Chemical (St. Louis, MO, USA), Gibco and Bachem AG (Bubendorf, Switzerland).

The cell type used was THP-1 monocytes (ATCC Number TIB-202). Culture media, penicillin (P), streptomycin (S), and fetal bovine serum (FBS) were from Gibco (Life Technologies SA, Madrid, Spain). LPS from Escherichia coli O55:B5 were purchased by Sigma Chemical Co. (St. Louis, MO, USA).

### 2.2. Hydrolysis of Sacha Inchi Protein Isolate

SII hydrolysis was carried out under continuous stirring, using a magnetic stirrer (Stuart, UK), at controlled pH and temperature conditions. The SII was resuspended in distilled H_2_O (10% *w*/*v*) at 50 °C. Bioprotease LA-660, a food-grade endoprotease, was added at a ratio enzyme/substrate = 0.3 AU (Anson units)/g protein (pH 8) for 5, 10, 15, 20, 30, and 60 min. In order to ensure the complete inactivation of any remaining enzyme activity, each sample was heated for 15 min, at 85 °C. Hydrolysates were constituted by the supernatants obtained after centrifugation at 8500 rpm for 15 min, named Sacha inchi protein hydrolysates 5 (SIH5B), 10 (SIH10B), 15 (SIH15B), 20 (SIH20B), 30 (SIH30B), and 60 (SIH60B); the numbers indicate the hydrolysis time (in minutes), and the letter “B” represents the enzyme Bioprotease. The supernatant of each sample was lyophilized in a Freeze Mobile 3 model equipment (VirTis Co., Gardiner, NY, USA), at 60 atm of vacuum pressure and −38 °C.

### 2.3. Evaluation of Hydrolysis Degree

The *Hydrolysis Degree* (HD) was determined by the reaction of free amino groups with 2,4,6-trinitrobenzenesulfonic acid (TNBS) [34]. A sample that had been hydrolyzed with HCl (6N) for 24 h at 110 °C was used as a measure of the total number of free amino groups.

### 2.4. Proximate Composition Analysis of Sacha inchi Protein Products

Proximal composition, including moisture, protein, ash, fat, and carbohydrates, was performed by following the official methods. Degree of moisture was defined at 110 °C to constant weight. The total protein matter was estimated as % nitrogen content × 6.25 by elemental microanalysis, using LECO TRUSPEC MICRO (Leco Corporation, St. Joseph, MI, USA). Ash content was analyzed according to the ignition method, at 550 °C, for 36 h, in a muffle furnace. Fat determination was assessed by the Soxhlet method with hexane. Estimation of both phenolic total compounds and soluble sugars was assessed using standard curves, corresponding to chlorogenic acid and glucose, respectively [35,36].

### 2.5. Determination of Amino Acid Composition by Ultrahigh-Performance Liquid Chromatography (UHPLC)

Determination of amino acid compounds was carried out in accordance with the protocol described by Alaiz et al. [37], with minor modifications for hydrolyzed proteins. Samples were hydrolyzed by 24 h incubation with a volume of 4 mL of 6 N HCl, at 110 °C, in sealed tubes, under nitrogen. Following hydrolysis, samples were evaporated at 50 °C, using an evaporator, and subsequently redissolved with 1 M sodium borate and 0.02% sodium azide, at pH 9. Amino acid composition was defined in the acid hydrolysate with UHPLC (Acquity Arc, Waters, Massachusetts, USA), after a derivatization protocol with diethyl ethoxymethylenmalonate, using a 3 mm × 150 mm reversed-phase column (XSelect HSS T3 XP, 2.5 μm; Waters) and D, L-α-aminobutyric acid as the internal standard. One binary gradient system was employed with the solvents (A) 25 mM sodium acetate 0.02% sodium azide (pH 6.0) and (B) acetonitrile. For each amino acid, calibration curves were built with a mixture of amino acid standard under the same hydrolysis conditions of the samples (Merck, Madrid, Spain), and the resulting values were analyzed using EMPOWER (Waters, Santa Clara, CA, USA). In addition, tryptophan presence was evaluated following the method of Yust et al. [38].

### 2.6. Scanning Electron Microscopy (SEM) Analysis

Ultrastructural morphology characterizations of SII and protein hydrolysate obtained with Bioprotease LA-660 were performed by SEM. For that purpose, a thin layer of each sample was mounted using sticky conductive carbon tape on an aluminum stub, removing any unattached particles. The specimen holder was then sputter-coated under vacuum, using a Leica EM ACE600 high-vacuum coater (Leica Microsystems, Wetzlar, Germany), with approximately 20 nm gold–palladium. Images were observed with a Zeiss Cross-beam 550 scanning electron microscope (Zeiss, Madrid, Spain), using 2.00 kV accelerating voltage, and with different magnifications [31].

### 2.7. Pepsin-Pancreatic Digestibility

Protein digestibility in vitro was established following the method of Sinda-yikengera and Xia [39], with some modifications. Pepsin and pancreatin enzymes were acquired from Sigma Aldrich (Num CAS: 9001-75-6 and 8049-47-6 respectively). Pepsin is a native pepsin from porcine stomach mucosa secreted by the mucosal lining of the stomach, which is active under acidic conditions and catalyzes the hydrolysis of aminoacyl-proline to an amino acid and proline. Inhibitors include pepstatin A, aliphatic alcohols, and pH > 6.0. Pancreatin from porcine pancreas, is a combination of various digestive enzymes produced by the exocrine cells of this organ. It is a broad-spectrum protease composed of protease, trypsin, amylase, lipase, and ribonuclease. This combination of enzymes allows the hydrolysis of proteins, starch, and fats. The sample (100 mg) was added in a tube with 7.5 mL of pepsin prepared from 0.1 M HCl, at a concentration of 0.2 mg/mL, and then incubated for 2 h at 37 °C. After that, the suspension was neutralized with 0.5 M NaOH and subsequently treated with 3.75 mL of pancreatin, prepared from 0.2 M phosphate buffer (pH 8), at a concentration of 0.5 mg/mL. The mixture was gently shaken and then incubated at 37 °C for an additional 2 h. After incubation, the sample was treated with 5 mL of 10% TCA (trichloroacetic acid) and centrifuged at 10,000× *g*, at room temperature, for 20 min. Supernatant proteins were calculated as the % nitrogen content × 6.25 by elemental microanalysis in a Leco CHNS932 analyzer (St. Joseph, MI, USA). Protein digestibility was calculated as the percentage resulting from the ratio of protein in supernatant to total protein in sample.

### 2.8. Molecular Weights (MWs) by Fast Protein Liquid Chromatography (FPLC)

MWs of protein from the Sacha inchi products were estimated by gel filtration chromatography on an Acquity Arc equipped with a 2998 PDA Detector, a Sample Manager FTN-R, and a Quaternary Sol-vent Manager-R (Acquity Arc, Waters corporation, Milford, MA, USA). A prepacked chromatography column for high-performance size exclusion, Superose 12 10/300 GL, with molecules with MWs ranging from 1 to 300 kDa, was used. The following standards from Pharmacia biotech were used: Dextran Blue (2000 kDa), aldolase (158 kDa), bovine serum albumin (67 kDa), Conalbumin (75 kDa), ribonuclease A (13.7 kDa), and bacitracin (1423 kDa). Using both the logarithms of the MWs of these standard proteins and their elution volumes, a calibration line was prepared. For the elution, 50 mL of sodium phosphate buffer 100 mM and sodium azide 0.02% (*w*/*v*) adjusted at pH 6.8, with a flow of 1 mL/min, was used. The injected volume of the samples was 500 μL at a concentration of 1 mg protein/mL. The elution of the proteins was registered by the measurement of their absorbance at 280 nm.

### 2.9. Determination of Antioxidant Activity

#### 2.9.1. DPPH Radical Scavenging Activity

Sacha inchi hydrolysates’ effects on scavenging of 2,2-diphenyl-1-picrylhydracil (DPPH) free radical were evaluated according to Wu et al. [40], with slight modifications. A plate of 96 wells with different concentrations of BHT and samples was used. The range of BHT concentrations used was from 0.05 to 0.001 μg/µL, and in the case of samples, from 5 to 0.1 μg/µL. A negative control was treated under the same conditions, using distilled water instead of sample. The final volume used in each well was 150 µL. The mixture was stirred and incubated at room temperature for 30 min, in the dark, and absorbance was measured at 517 nm. Radical DPPH has an absorption band centered at about 517 nm which disappears when an antiradical compound reduces it. Thus, lower absorbance of the reaction mixture means higher DPPH scavenging activity. The IC50 DPPH is defined as the effective concentration that sequesters 50% of the free radicals; it is calculated via a non-linear regression from a plot of % DPPH activity versus sample concentration (μg/μL).

#### 2.9.2. Ferric Ion Reducing Antioxidant Power (FRAP)

The activity of Sacha inchi hydrolysates to reduce iron (III) was assessed according to Oyaizu [41]. A 96-well plate with different concentrations of BHT and samples was used. The range of BHT concentrations used was from 0.06 to 0.001 μg/µL, and in the case of samples, it was from 2.5 to 0.01 μg/µL. Briefly, 20 μL of sample, BHT, or water was added to each well with 50 μL of 0.2 M sodium phosphate buffer at pH 6.6 and 50 μL of potassium ferricyanide (0.03 M). The mix was incubated at 50 °C for 20 min, in the dark. Then, 50 μL of trichloroacetic acid (0.6 M) and 10 μL of ferric chloride (3.7 mmol/L) were added and incubated for 10 min at 50 °C, in the dark. After this, absorbances were measured at 700 nm. Higher absorbance values of the reaction mixture relate to an increase in the reducing power. The IC50 reducing power is defined as the effective concentration of the samples for which the absorbance is 0.5 for reducing power; it is calculated by non-linear regression from a plot of % reducing power activity versus sample concentration (μg/μL).

#### 2.9.3. Determination of the Antioxidant Activity by β-Carotene–Linoleic Acid Assay

The assay was determined by the Marco [42] protocol, with a few modifications to perform the test on a microplate. The method is based on determining the discoloration of β-carotene by the oxidation products of linoleic acid. For this purpose, a solution of BHT (dissolved in ethanol) is used as a positive control, while water serves as a negative control. A 96-well plate with different concentrations of BHT and samples was used. The range of BHT concentrations used was from 0.05 to 0.001 μg/µL, and in the case of samples, it was from 4.5 to 0.1 μg/µL. In brief, 10 mL of chloroform was added to 2 mg of β-carotene. After that, a mixture of 200 mg of tween 20, 20 mg of linoleic acid, and an aliquot of 1 mL β-carotene solution was prepared, and the chloroform was removed under vacuum, using a rotary evaporator. The resulting solution was vigorously stirred after adding 20 mL of distilled water enriched with oxygen. Finally, 20 μL of sample, BHT, or water and 200 μL of β-carotene reagent described before were added to each well, and the absorbance was measured at 10, 20, 30, 40, 50m and 60 min. The antioxidant activity (%) was calculated using the index protection (IP) with the following equation: IP = (A/A0) × 100, where A is absorbance at 60 min, and A0 is the absorbance at time zero. The IC50 is the effective concentration that inhibits 50% of β-carotene. It is calculated by nonlinear regression from a plot of % antioxidant activity versus sample concentration (μg/μL).

### 2.10. THP-1 Culture

THP-1 human monocytic cells were cultured using RPMI 1640 medium supplemented with 1% P/S and 10% heat-inactivated FBS. Cells’ culture was prepared in 12-well plates at 1 × 10^6^ cells/well. They were maintained in 5% CO_2_ at 37 °C in a CO_2_ incubator (Thermo Con Electron Corporation, Waltham, MA, USA). Four different conditions were used: untreated cells (negative control); cells exposed to 100 ng/mL of LPS as positive control; and cells exposed to 100 ng/mL of LPS + SIH20B at 50 or 100 µg/mL. These intermediate concentrations of SIH20B, previously evaluated in the cell viability assay, had shown good biological activities in monocytic cells with other plant protein hydrolysates [43]. LPS (*E. coli* 055: B5) (Sigma-Aldrich) was added 1 h before SIH20H. Following an additional period of 24 h of incubation, the medium was collected for ELISA, and the cells were processed for RNA extraction.

### 2.11. Cell Viability (MTT)

THP-1 cells were incubated with different amounts of the SIH20B in 96-well plates at 1 × 10^5^ cells/well for 24 h. Concentrations used were the following: 10, 50, 100, 250, and 500 µg/mL. Afterwards, 3-(4,5-dimethylthiazol-2-yl)-2,5-diphenyltetrazol bromide and MTT solution (Sigma-Aldrich, Madrid, Spain) were added to the wells and incubated for 3 h, until a purple precipitate was visible. MTT–formazan crystals were solubilized with dimethyl sulfoxide (DMSO, Sigma-Aldrich) and then measured with a Thermo Scientific Fluoroskan Ascent^®^ (ThermoFisher Scientific, Madrid, Spain) microplate reader at 570 nm corrected to 650 nm. Cell survival is based on the metabolic reduction of MTT absorbance compared with that obtained in control, non-treated cells, carried out by the succinate dehydrogenase mitochondrial enzyme, leading to the formation of a purple-colored compound (formazan), enabling the mitochondrial functionality of the treated cells to be determined [44]. The ability of the cells to reduce MTT is then an indicator of the integrity of the mitochondria, and their functional activity is accepted as a measure of cell viability.

### 2.12. RNA Extraction and RT-qPCR

RNA was extracted from THP-1 cells, using TRIsure reagent (Bioline, Almería, Spain), following the manufacturer’s instructions. Total RNA was extracted with TRIsure Reagent (Bioline, Memphis, TN, USA). RNA quality was estimated by A260/A280 ratio in a NanoDrop ND-1000 Spectrophotometer (ThermoFisher Scientific, Madrid, Spain). One microgram of total RNA was reverse transcribed with the iScript kit (Bio-Rad, Madrid, Spain), according to manufacturer’s protocol. Then, 10 ng of the resulting cDNA was used as template for qRT-PCR amplifications. For every PCR reaction, cDNA template was added to Brilliant SYBR green QPCR Supermix (Bio-Rad) containing the primer pairs for either gene or for a housekeeping gene (hypoxanthine phosphoribosyltransferase (HPRT)/glyceraldehyde 3-phosphate dehydrogenase (GAPDH)). Amplification qRT-PCR reactions were carried out in triplicate, and the average threshold cycle (Ct) values of them were the reference to calculate the relative expression of mRNA for every gene tested. The measurement of changes in mRNA expression was determined by the 2^−(ΔΔCt)^ standard method. The endogenous reference HPRT and GAPDH genes were used to normalize all data, which are depicted as relative values compared to the control. Individual fold changes resulting from every pair of samples coming from the two compared groups were estimated. Finally, the media of these individual values represent the overall fold change for the given gene. Table 1 shows the sequences of the designed oligonucleotides.

### 2.13. Extracellular Quantification of Cytokines

TNF-α, IL-1β, IL-6, and IL-10 concentrations in cell culture supernatants were quantified by commercial ELISA kits, following the manufacturer’s instructions (Diaclone, Besancon, France). Cytokine concentrations were expressed in pg mL^−1^, as calculated from the calibration curves from serial dilution of human recombinant standards in each assay.

### 2.14. Peptide Extraction, Purification, and Sequence Identification by LC-TIMS-MS/MS

Samples were acidified using 0.5% trifluoroacetic acid. The desalting and concentration step was assessed with ZipTip C18 (Millipore, Madrid, Spain), and samples were then speed-vacuum-dried. LC-TIMS-MS/MS was carried out using a nanoElute nanoflow ultrahigh-pressure LC system (Bruker Daltonics, Bremen, Germany) coupled to a timsTOF Pro 2 mass spectrometer, equipped with a CaptiveSpray nanoelectrospray ion source (Bruker Daltonics). In brief, about 200 ng of peptide digest was loaded onto a Bruker FIFTEEN C18 capillary column (15 cm length, 75 μm ID, 1.9 μm particle size, 120 Å pore size; Bruker Daltonics). Peptides were separated at 30 °C, using a 20 min gradient, at a flow rate of 300 nL/min (mobile phase A (MPA): 0.1% FA; mobile phase B (MPB): 0.1% FA in acetonitrile). A step gradient from 0 to 35% MPB was applied over 13 min, followed by a 35-to-90% MPB step of 13-to-15 min, and it finished with a 90% MPB wash for an additional 5 min for a further time. TimsTOF Pro 2 was run in DDA-PASEF mode. Mass spectra for MS and MS/MS scans were recorded between 100 and 1700 *m*/*z*. Ion mobility resolution was set to 0.85–1.30 V s/cm^2^ over a ramp time of 100 ms. Data-dependent acquisition was assessed using 4 PASEF MS/MS scans per cycle, with a duty cycle close to 100%. A polygonal filter was applied on the *m*/*z* space and ion mobility to exclude low *m*/*z*, mainly single-charged ions from the selection of PASEF precursors. An active exclusion time of 0.4 min was applied to precursors that reached 20,000 intensity units. The collision energy was increased stepwise as a function of the ion mobility ramp, from 27 to 45 eV. The raw data were examined in PEAKS Studio ProX (Bioinformatics Solution Corp). Protein unique peptides was set to larger than 1, and a high confidence score of −10lgP > 20 was applied to indicate an accurately identified protein.

### 2.15. In Silico Analysis

For the 20 most abundant peptides with a molecular weight <1000 Da identified in SIH20B, the following in silico analyses were performed: (a) the net charge of identified peptides at neutral pH and the isoelectric point, hydrophobicity, amphipathicity, and steric hindrance were estimated by ToxinPred1 software (accessed on 21 May 2024) (https://webs.iiitd.edu.in/raghava/toxinpred/design.php) [45]; (b) the solubility was calculated via Peptide Property Calculator (accessed on 21 May 2024) (http://pepcalc.com/) [46]; (c) PeptideRanker (accessed on 21 May 2024) was used to predict the probability of being bioactive (http://bioware.ucd.ie/~compass/biowareweb), giving a score from 0 to 1.0, at a threshold of 0.5 [47]; (d) PreAIP (accessed on 21 May 2024) (http://kurata14.bio.kyutech.ac.jp/PreAIP/) was used to predict the most anti-inflammatory sequences responsible for the bioactivity [48]; and (e) AnOxPePred-1.0 (accessed on 21 May 2024) (https://services.healthtech.dtu.dk/service.php? AnOxPePred-1.0) was used to predict the antioxidant (quantified by free radical scavenging and ion chelating scores) properties of peptides, using the convolutional neural network [49].

### 2.16. Statistical Analysis

All values are expressed as arithmetic means standard deviations (SDs). Graph Pad Prism Version 6.01 software (San Diego, CA, USA) was used to evaluate data. One-way analysis of variance (ANOVA), followed by Tukey’s multiple comparisons test as a post hoc test, was used to calculate the statistical significance of differences in each parameter among the groups. A *p*-value of less than 0.05 was considered statistically significant.

## 3. Results

### 3.1. Chemical Characterization of Sacha Inchi Protein Isolate and Different Sacha Inchi Hydrolysates

**Figure 1:**
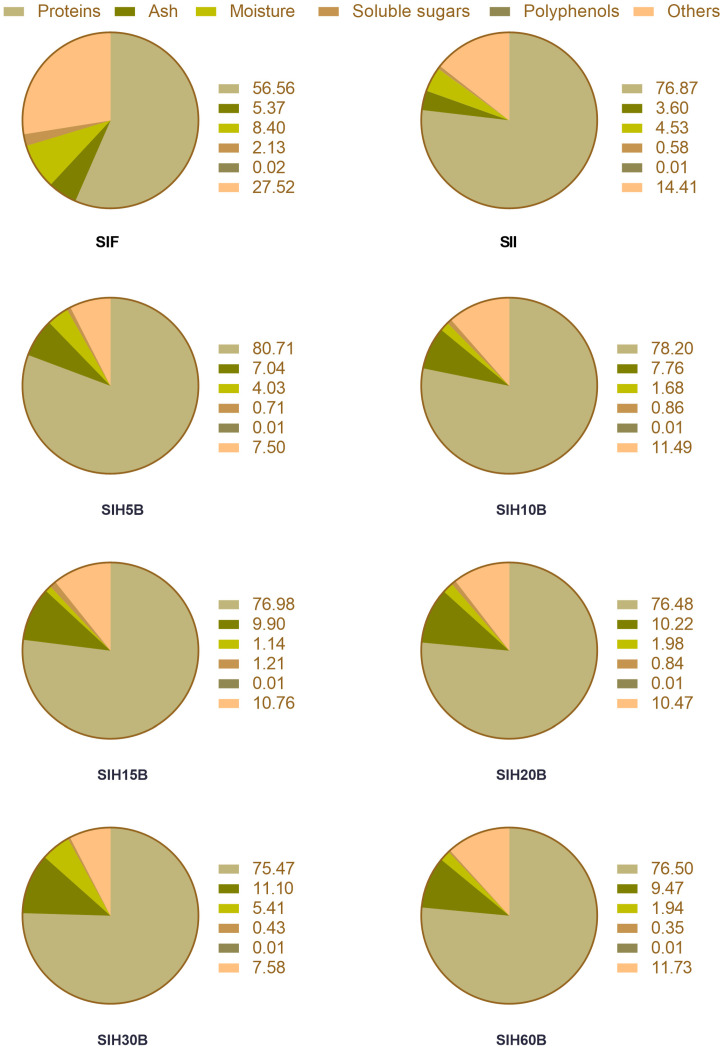
Chemical composition (%) of Sacha inchi protein products. Sacha inchi flour (SIF), Sacha inchi isolate (SII), and Sacha inchi hydrolysates at different time: 5 (SIH5B), 10 (SIH10B), 15 (SIH15B), 20 (SIH20B), 30 (SIH30B), and 60 min (SIH60B). Others: 100 − (proteins + moisture + ash + sugars + polyphenols). Values shown refer to the mean as a result of three determinations.

The production of protein isolate mainly consists of three stages: (i) protein extraction, (ii) isoelectric precipitation, and (iii) drying of the precipitated protein. Alkaline extraction enables the removal of undesirable compounds in the protein isolate, fiber, sugars, lipids, polyphenols, and alkaloids and the preparation of a protein isolate for high-quality products, facilitating protease activity during hydrolysis [50]. The proximal composition of Sacha inchi protein products is shown in Figure 1.

The protein content of SII is 76.87% on a dry basis. These values are similar in the different Sacha inchi protein hydrolysates. SIF, from which SII is obtained, has a protein richness of 56.56%, higher than defatted flour of emerging crops like chia (34.95%) [51], hemp (33.30%) [52], or kiwicha (15.88%) [31], and similar to traditional crops as defatted soybean meal, which is sold, according to commercial standards, with a minimum protein content of 50% by dry weight [53]. As regards ash content, a higher ash content is observed in the hydrolysates as a consequence of the addition of sodium hydroxide during the hydrolytic process to keep the pH constant [54]. The polyphenol content of the defatted meal and protein isolate was less than 0.05%. In this respect, low levels of polyphenols are desirable from a functional and nutritional point of view because of the possible interactions they may have with proteins present in the food matrix [55].

**Figure 2:**
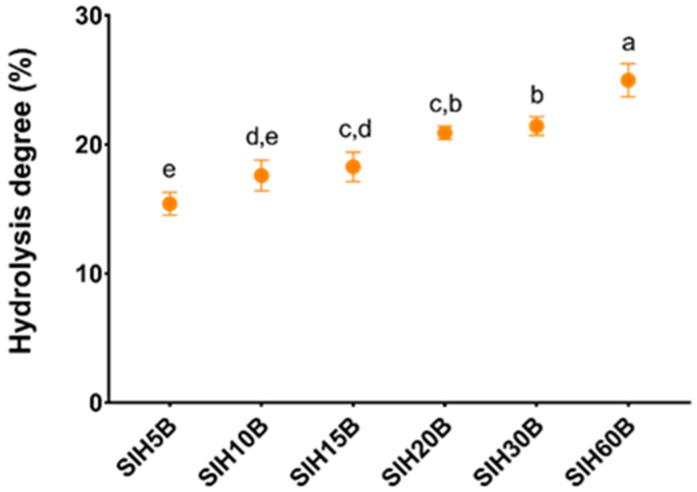
Hydrolysis degree during enzymatic hydrolysis by Bioprotease of SIH5B, SIH10B, SIH15B, SIH20B, SIH30B, and SIH60B. Left *y*-axis data refer to the percentage of the cleaved bonds in the peptide structure. Every shown value is the result of three determinations and depicted as means ± standard deviation. Different letters relate to statistical differences (*p* < 0.05), as determined through one-way ANOVA, followed by a post hoc Tukey test.

Performing enzymatic processes under soft conditions minimizes undesirable by-products produced by chemical hydrolysis. Protein hydrolysates obtained by this method have technological advantages, such as improved solubility and a decrease in the potential allergenicity, due to an increase in polar groups (-NH_4_^+^, -CO^2−^) and a disruption of linear and conformational epitopes [56], in addition to better functional properties than the raw protein due to the release of bioactive peptides present in the original protein, with biological activities such as antioxidant or opioid activities [57]. These functional properties depend on the HD, which measures the number of peptide bonds that have been broken relative to the total number of peptide bonds in the original protein. It is well known that extensive hydrolysis produces small peptides (1–3 kDa) that are more likely to be bioavailable and bioactive. The extensive hydrolysis of SII was performed using Bioprotease LA-660, an enzyme obtained from a Bacillus subtilis strain and that has a high proteolytic capacity, hydrolyzing proteins in a non-specific manner, in the way represented in Figure 2. The hydrolysis rate increased during the first 5 min and then remained stable for the next 55 min, reaching a maximum HD of 23.34% in SIH60B. These values coincide with other hydrolysates carried out on Sacha inchi cake with papain, another food-grade enzyme [58], although they differ from other hydrolysates of quinoa, with Bioprotease LA-550 obtaining a degree of hydrolysis of 31% although the time used for hydrolysis was 180 min, much longer than the hydrolysate with the maximum time of this trial, which was 60 min [59]. The results obtained with respect to HD using Bioprotease are similar to other studies using food-grade enzymes, such as Alcalase or Protamex, indicating that Bioprotease is a good option as an endoprotease enzyme in obtaining protein hydrolysates [60].

The digestibility of seed proteins is limited by the presence of trypsin and chymotrypsin inhibitors, as well as by the globular structure of the proteins [61]. In addition, during the preparation of the isolates, there is a partial denaturation of the proteins which enhances subsequent enzymatic hydrolysis by making the digestive enzymes more accessible to their site of action [62]. Sacha inchi hydrolysates have a high protein digestibility in vitro, close to 100%, while that of SII was just over 50%, so it was observed that enzymatic hydrolysis improved this digestibility. The values obtained in this study are higher than other digestibility studies carried out by different authors, probably due to the use of different enzymes for the hydrolysis [58].

In summary, Sacha inchi hydrolysates appear to be a good source of high-quality protein, with high protein digestibility. However, it is important to remember that protein digestibility is not the only factor to consider in protein quality, the abundance of essential amino acid, and the quantity and quality of other nutrients should also be considered [63,64].

### 3.2. Amino Acid Composition of Sacha Inchi Protein Products

All Sacha inchi protein products meet the nutritional requirements proposed by FAO/WHO (Table 2), with the exception of lysine in the case of SII and Lys, and leucine and valine in the case of hydrolysates. This coincides with the amino acid profile of different Sacha inchi seeds provided by Ruiz et al. [16]. Regarding essential amino acids (Table 2), Sacha inchi is rich in tryptophan, phenylalanine, and threonine, and it also contains high amounts of isoleucine and histidine. For non-essential amino acids (Table 3), the highest content was glutamic acid and aspartic acid. Significant amounts of arginine and serine were also found in Sacha inchi protein products. Legumes are often poor in sulfur amino acids, methionine, and cysteine, but in the case of Sacha inchi, this limitation did not occur, and it presents adequate values of these amino acids [65,66]. The predominant essential amino acids were the aromatic amino acids (phenylalanine, tyrosine, and tryptophan). Tyrosine is a natural antioxidant that helps protect cells from oxidative damage. In line with this, tripeptides with tryptophan and tyrosine at their C-terminus showed strong radical scavenging activity. The antioxidant ability of the two aromatic amino acids, tryptophan and phenylalanine, has been connected with their capacity to act as radical scavengers, and the antioxidant activity of tyrosine is related to the special capability of phenolic groups to act as hydrogen donors [67]. Phenylalanine and tyrosine can also act as regulators of gene expression, which means they can influence how certain genes are turned on and off [68].

### 3.3. Antioxidant Effects of Sacha Inchi Protein Products

Different assays are used to determine the exogenous antioxidant capacity of foods based on the description of the ability of redox molecules to scavenge free radicals. Exogenous antioxidants can be used to prevent damaging effects of the excessive amounts of free radicals in cells, through free radical scavenging, reduction of hydroperoxides, or chelation of prooxidant transition metals [69]. Many peptides that exert antioxidant bioactivity are being recognized as food ingredients and as supplements in nutraceuticals and functional foods [70]. Up to three protocols were carried out to elucidate the antioxidant potential of Sacha inchi protein products: DPPH free radicals sequester ability, ferric ion reducing antioxidant power (FRAP), and β-carotene–linoleic acid assay. The results (Table 4) indicated that the SIH20B had a higher free radical scavenging capacity in the DPPH assay than the isolate and the other hydrolysates.

In terms of FRAP activity, the isolate and the hydrolysates showed similar antioxidant activity, with no significant differences between them. In addition, antioxidant activity was measured by means of the β-carotene assay. The results in this case showed that the hydrolysates with the longest hydrolysis time (SIH20B, SIH30B, and SIH60B) had the highest antioxidant activity. Although certain aspects of the structure–function relationship of antioxidant peptides are not yet well understood, it is suggested that the most crucial factors affecting antioxidant properties relate to amino acid composition and sequence [68]. It is suspected that the antioxidant activity shown by Sacha inchi protein products is due to the increased presence of aromatic amino acids Trp, Phe, Tyr, and His, which have been linked to strong free radical scavenging activities through direct electron transfer. Particularly, the potent radical scavenging and metal chelating ability of His has been credited to its imidazole ring, as the aromatic side chain can contribute to hydrogen atom transfer (HAT) and single-electron transfer reactions [71,72]. After the determination of the antioxidant capacity of the different Sacha inchi hydrolysates, SIH20B was selected to study the anti-inflammatory activity due to the fact that it has the best results in regard to its antioxidant capacity. In addition, it is economically more efficient to use a short-time hydrolysis to obtain a protein-rich product.

### 3.4. Sacha Inchi Products Remodel Their Surface Ultrastructure after Enzymatic Hydrolysis

**Figure 3:**
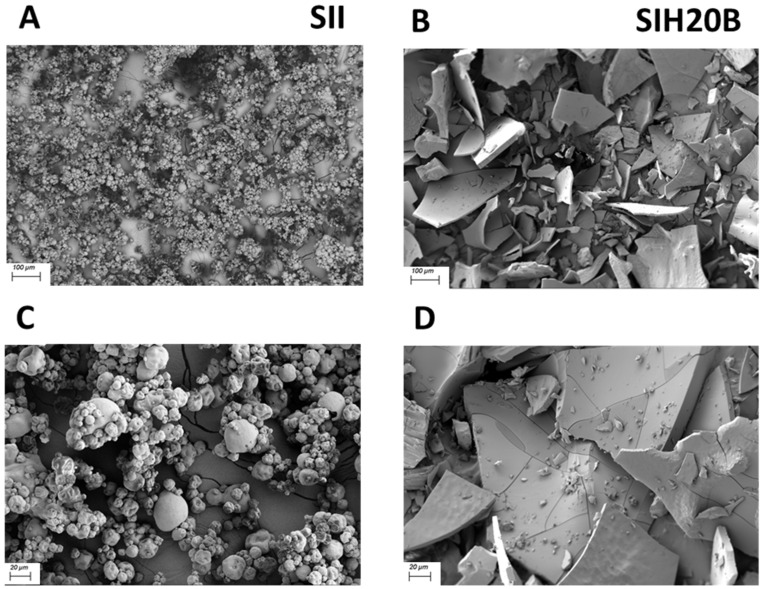
Ultrastructural characteristics of SII and SIH20B by SEM at various magnifications. SEM images (**A**,**B**) were taken at Mag = 90×, and SEM images (**C**,**D**) were taken at Mag = 345×.

Figure 3 shows the surface morphologies of SEM images of both SII and SIH20B at various magnifications. Electron microscopy facilitates the observation of the changes in the surface of the hydrolysates in comparison to the isolate, as the protein’s irregular and granule aggregates turn to flattened fragments after enzyme activity. The new protein pattern relates to the peptidic bonds cleavage, which leads to a reduction in particle size. Images were taken using the same microscope parameters (Mag = 90× and AV = 2.0 kV/Mag = 345× and AV = 2.0 kV).

This ultrastructure characterization is in line with other studies that show protein aggregation or fracture pattern changes as a parameter related to a reduction in samples particle size and solubility [73,74,75].

### 3.5. Molecular Profile of Sacha Inchi Protein Products

**Figure 4:**
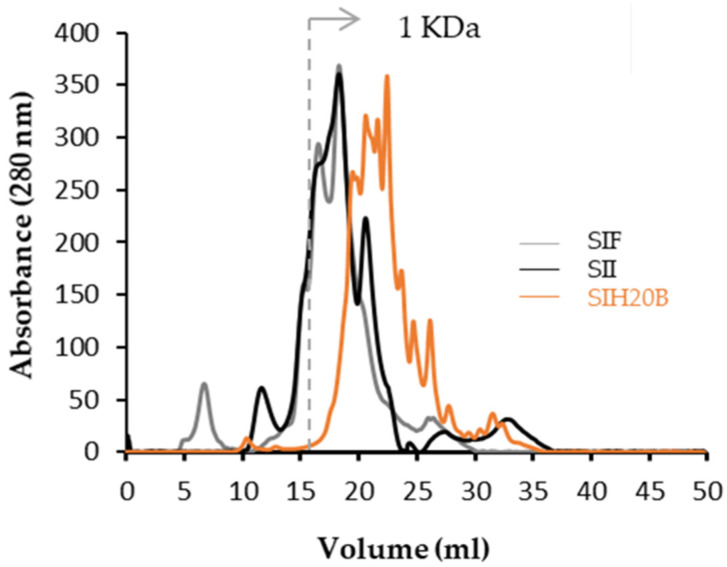
Molecular-weight profiles by size-exclusion UHPLC of SIF, SII, and SIH20B.

Figure 4 shows how most of the proteins and polypeptides contained in SIF and SII are between 50 and 3 kDa and how the hydrolysis process with enzyme Bioprotease potentially reduced the molecular-weight values of most the SIF and SII proteins, achieving 1 kDa in SIH20B. Although, the column used is not capable of reliably resolving peptides smaller than 1 kDa, the molecular profile of SIH20B demonstrates the existence of peptides smaller than 1 kDa and justifies the study of the peptidomes of said hydrolysate to identify and quantify them, since it has been reported that the peptides with the highest bioactivity have a molecular-weight distribution between 0.4 and 2 kDa (2–20 amino acids), and they are also the most resistant to degradation by gastrointestinal proteases and, therefore, are potentially able to reach the bloodstream intact [76,77].

### 3.6. Cell Viability

**Figure 5:**
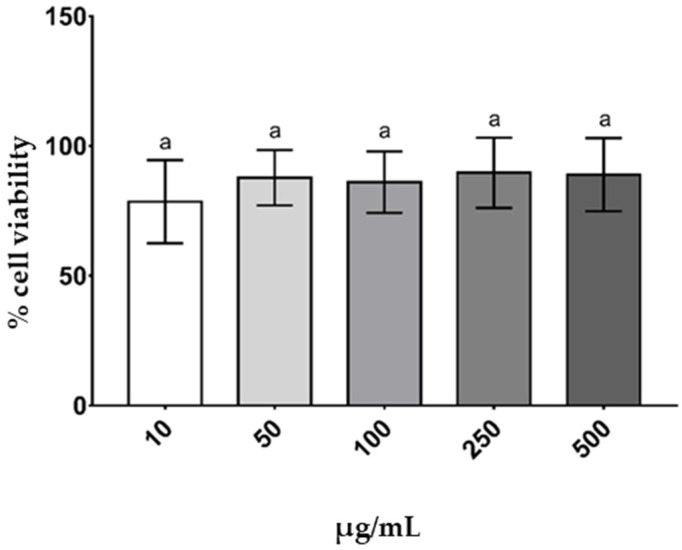
Effect of SIH20B on viability of THP-1. Cells were treated with SIH20B (10–500 μg/mL) for 24 h. According to MTT assay, cell viability was expressed as percentage of absorbance relative to control (untreated) cells. Experiments were carried out in triplicate, at least three independent times. Values are presented as means ± SD, and those marked with different letters are significantly different (*p* < 0.05), as determined through one-way ANOVA, followed by a post hoc Tukey test.

THP-1 monocytes cells were incubated with SIH20B, with concentrations ranging from 10 to 500 μg/mL, for 24 h. Cell viability determined by the MTT method and was not affected by any SIH20B concentration (Figure 5), which means that the present compound does not affect the cell integrity.

### 3.7. SIH20B on Cytokine Gene and Protein Expression in THP-1 Cell Culture

**Figure 6:**
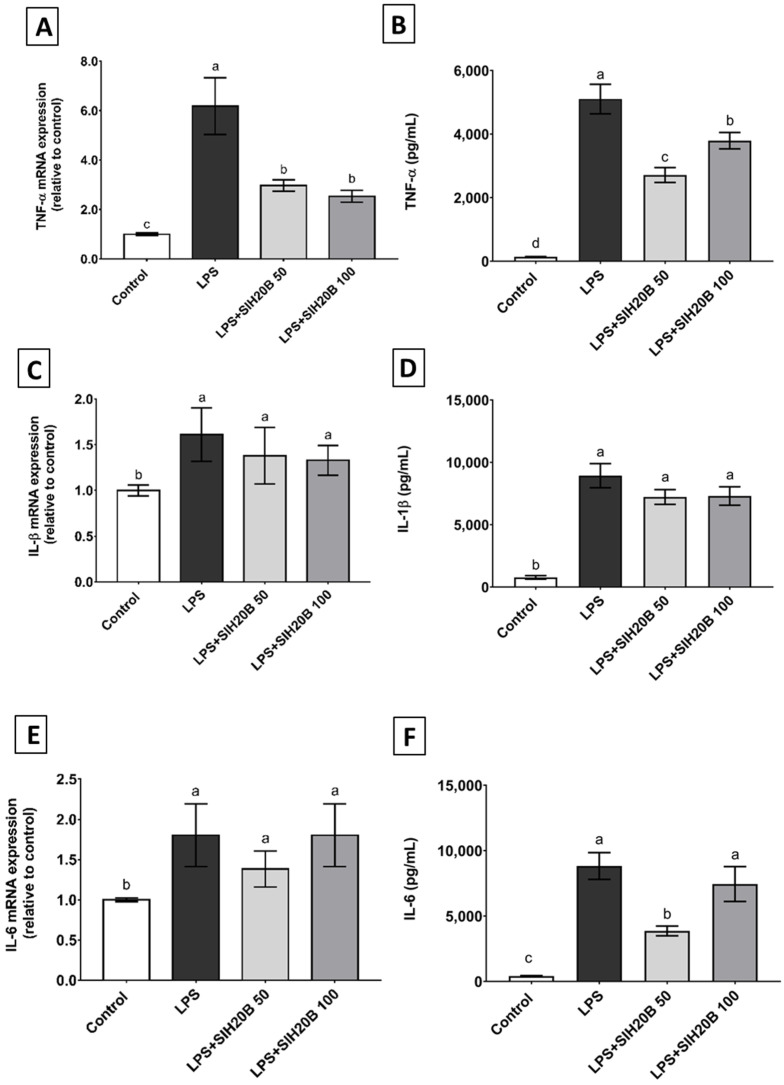
Effect of SIH20B on expression of pro-inflammatory cytokine genes and proteins. The mRNA levels and the release of cytokines of TNF-α (**A**,**B**), IL-1β (**C**,**D**) and IL-6 (**E**,**F**) were measured after the treatment of THP-1 cells with LPS (100 ng/mL) in the absence or presence of SIH20B at 50 and 100 μg/mL. Control means untreated cells and the experiments were carried out in triplicate. Values are presented as means ± SD of three determination, and those marked with different letters are significantly different (*p* < 0.05), as determined through one-way ANOVA, followed by a post hoc Tukey test.

Inflammatory processes involve the cell activation of circulating monocytes [78] and release many kinds of cytokines to enhance the capacity for cellular defense, including pro-inflammatory cytokines, such as TNF-α, IL-1β, or IL-6; and anti-inflammatory cytokines, such as IL-10 or IL-4 [79]. Oxidative stress and inflammation, in which proinflammatory cytokines play a crucial role, favor the development of many metabolic diseases, such as obesity or diabetes [80]. LPS is known for its monocyte/macrophage activation, and its effect causes the stimulated release of important modulator molecules, including pro-inflammatory cytokines and chemokines [81]. In order to study the anti-inflammatory activity of SIH20B, the expression of genes such as TNF-α, IL-1β, and IL-6, as well as IL-10 and IL-4 mediators, was estimated using the RT-qPCR method in the THP-1 monocytes cell line under LPS stimulation. The protein level of those genes was also measured by ELISA in the supernatant of the cell culture. As expected, LPS increased mRNA levels of TNF-α, IL-1β, and IL-6 genes (Figure 6A,C,E). However, these LPS-induced changes were blocked in the presence of SIH20B at 50 or 100 μg/mL. Both doses were effective in reducing the expression levels of TNF-α gene in comparation with the cells treated with LPS in a significant way. These results are similar to those of other studies made with Sacha inchi protein products where the authors demonstrated that albumin fraction of SI seeds exhibited immunomodulatory properties by stimulating spleen lymphocytes [12]. It is widely known that there are peptides that, when released from the original protein, can have different biological activities, such as antioxidant or anti-inflammatory activities [82]. In the literature, there are many studies where the immunomodulatory effects of plant protein hydrolysate were investigated [83]. For example, Lemus-Conejo et al. [84] described how an octapeptide called GPETAFLR, isolated from *Lupinus angustifolius* L., has a neuroprotective activity partially inhibiting the release of pro-inflammatory cytokines and promoting the survival of neurons and neuronal homeostasis, and the hydrolysate from which it was isolated showed an anti-inflammatory effect in the human monocytic cell line THP-1 [85]. Specifically, a bean protein hydrolysate had a similar effect to the hydrolysate in the present study, preventing increased TNF-α expression and modulating vascular permeability and LDL migration into the subendothelial space [86]. The results of cytokine mRNA expression are consistent with cytokine production in the supernatant of culture medium (Figure 6B,D,F): a decrease in protein levels assessed by ELISA could be observed not only for TNF at both concentrations, but also for IL6 at the lower concentration, despite the fact that no gene expression changes were observed for this cytokine.

**Figure 7:**
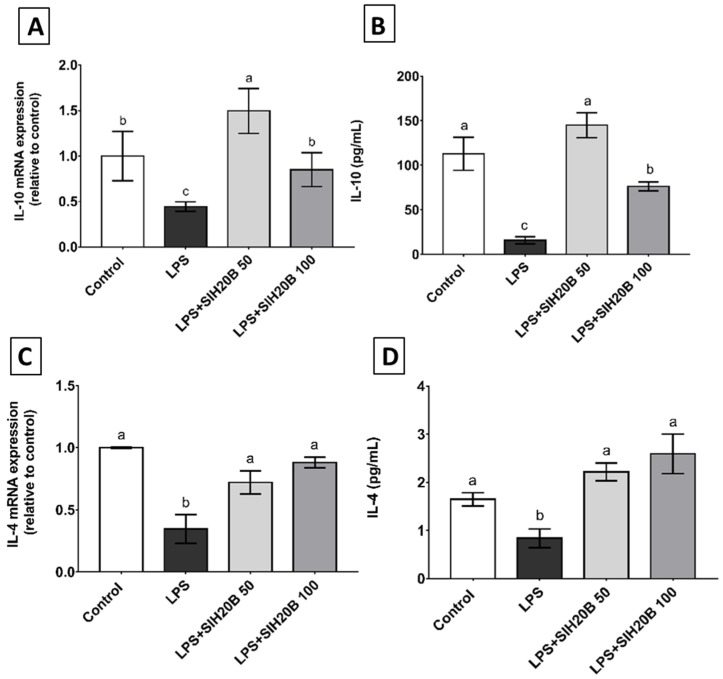
Effect of SIH20B on expression of anti-inflammatory cytokine genes and proteins. Levels of mRNA and the release of both IL-10 (**A**,**B**) and IL-4 (**C**,**D**) cytokine molecules were measured after the treatment of THP-1 cells with LPS (100 ng/mL) in the absence or presence of SIH20B at 50 and 100 μg/mL. Control means untreated cells and the experiments were carried out in triplicate. Values are presented as means ± SD of three determination, and those marked with different letters are significantly different (*p* < 0.05), as determined through one-way ANOVA, followed by a post hoc Tukey test.

Sacha inchi hydrolysate inhibited inflammatory phenomena by enhancing anti-inflammatory markers such as the cytokines IL-4 and IL-10. Despite the fact that LPS-activated-cells reduced the expression of IL-10 and IL-4 genes in THP-1 cells, co-administration with SIH20B counteracted this effect (Figure 7A,C). This effect is confirmed with the ELISA assay (Figure 7B,D), where the level of proteins was also increased with respect LPS-activated cells, indicating that SIH20B acts at both the transcriptional and post-transcriptional levels. Hempseed-derived proteins had a similar effect in glial cells, inhibiting inflammatory events by boosting anti-inflammatory markers, including IL-4 and IL-10 [87]. The results were similar in mung bean protein hydrolysate that could modulates the immune response through NF-κB pathway in lipopolysaccharide-stimulated RAW 264.7 macrophages, increasing IL-10 production [79]. In the forthcoming studies, it would be interesting to investigate the molecular mechanisms underlying the immunomodulation produced by the possible bioactive peptides present in the SIH20B.

### 3.8. In Silico Analyses of Identified Peptides

The peptidome of SIH20B was fully characterized by LC-TIMS-MS/MS. The number of peptides identified was 3788, including 842 peptides (22.23%) with a molecular weight under 1 kDa. In view of the high number of peptides that were identified, the selection criteria were the most relative abundance values (determined by area) with a molecular weight below 1 kDa, which is the size for those more easily absorbed peptides that can exert their bioactivity in the target cells [88]. Protein hydrolysates contain different numbers of components as polypeptides, small–medium peptides, and free amino acids, among others. This fact produced limitations regarding the identification of every single peptide. For this reason, bioinformatics tools could help to identify compounds that are biologically active [89]. In Table 5, data are represented in terms of charge, pI, hydrophobicity, stearic hindrance, amphipathicity, and solubility, calculated with different bioinformatics tools, such as ToxinPred and PepCalc. All peptides were predicted as non-toxic based on an analysis using Toxin Pred1 software.

The amino acid sequence is extremely important in order to predict the biological activities, involving the interactions of amino acids, electrostatic and hydrogen-bonding properties, the locations of amino acids, and the steric properties of the residues at the C- and N-terminal [90]. In the present study, the potential of each peptide to be biologically active was estimated with PeptideRanker [47] and was expressed as a free radical scavenger and anti-inflammatory activity, with the in silico tools PreAIP [48] and AnOxPePred [49], respectively.

On the other hand, amphiphilicity is an important feature in order to cross the membrane and enables the internalization of bioactive substances in cells. Moreover, the conformation and the length of the sequence is equally important as the accumulation of positive charge [91]. According to PeptideRanker, the peptides with the highest probability of being bioactive were GDGSLRML (0.8074), GDGSLRLM (0.8027), AAGALKKFL (0.7997), GNGSLRML (0.7512), LFPPPGKA (0.7289), LGVKFKGGL (0.6273), and PGVLKAPPP (0.5328), and the peptides with the highest amphipathicity were PGVLKKHP (1.10), followed by PQREVQSQ (0.93), NGVLKKFL (0.92), AAGALKKFL (0.82), and LGVKFKGGL (0.82).

Therefore, AAGALKKFL and LGVKFKGGL can be considered peptides with a high probability of being bioactive and also of being able to cross cell membranes. In the present research, the platform AnOxPePred [49] was used to evaluate the antioxidant activity of the selected peptides and PreAIP to evaluate anti-inflammatory activity. According to the developers of such in silico tools, the threshold to consider a peptide as anti-inflammatory with high confidence would be 0.468, and antioxidant, 0.5 [48,49]. Considering these limits, one of the two candidate peptides chosen above as potentially bioactive and amphipathic, AAGALKKFL, also shows potential anti-inflammatory activity in the PreAIP tool (0,478). However, it is the LFPPPGKA sequence which showed high bioactive potential and better antioxidant activity in AnOxPePred (0,521). The bioactivity of these peptides could be explained for the presence of hydrophobic amino acids His (H), Trp (W), Phe (F), Pro (P), Gly (G), Lys (K), Ile (I), and Val (V) in their sequences, which can improve the antioxidant capacity of peptides because they may lead to favorable hydrophobic microenvironments [92]. It is also known that imidazole, indole, and pyrrolidine rings that are present in the molecular structure of tryptophan and proline stabilize the formation of radical peptides following electron donation from ROS and free radicals [68,93].

It should be noted that the positive results of protein hydrolysates result from the depletion of the entire peptide pool, not only from specific peptides [74]. However, it must be considered that this is an approximation to predict the biological activities, but it is necessary to in vitro values with synthetic peptides, if available, in order to elucidate in animal models the mechanisms underlying the potential in vivo activity of these peptides and for human trials to suggest their healthy attributes.

## 4. Conclusions

SIH20B, which was obtained after 20 min of hydrolysis with the enzyme Bioprotease, is presented as a promising functional food ingredient due to its nutritional characteristics, technofunctionality, and its antioxidant potential, mainly due to its ability to scavenge the DPPH radical. Overall, our results in THP-1 monocytes suggest that the SIH20B could be used as a potential functional component to prevent diseases related to excess oxidative stress and local inflammation in chronic diseases due to the immunomodulatory activity that is exerted on cytokines involved in inflammatory processes, such as TNF-α or IL-10. In addition, the in silico analysis of its peptidome profile suggested that the more promising anti-inflammatory and antioxidant peptides are AAGALKKFL and LFPPPGKA, with the former being the most likely to be absorbed at the cellular level, due to its higher amphipathicity. Therefore, SIH20B is a novel, natural source of high-value-added biopeptides that could be used as a new ingredient in the formulation of healthy, green, high-quality food or nutraceutical compounds, like others vegetable protein hydrolysates of kiwicha [31], chia [51], or hemp [52], among others, as alternatives to protein hydrolysates of animal origin, such as whey, fish, or egg. However, further in vitro gastrointestinal digestion studies according to the INFOGEST method will be necessary in the future to consider whether other enzymes involved in food digestion, such as lipases, amylases, etc., influence the composition and bioactivity of the SIH20B digest, despite the low non-protein content of this functional ingredient [94].

## Figures and Tables

**Table 1:** Primer sequences for RT-qPCR gene expression analysis.

Gen	No. GenBank	Forward Reverse	Sequence (5′→3′)
TNF-α	NM_000594	Forward Reverse	TCCTTCAGACACCCTCAACC AGGCCCCAGTTTGAATTCTT
IL-1 β	NM_000576	Forward Reverse	GGGCCTCAAGGAAAAGAATC TTCTGCTTGAGAGGTGCTGA
IL-6	NM_000600	Forward Reverse	TACCCCCAGGAGAAGATTCC TTTTCTGCCAGTGCCTCTTT
IL-10	NM_000572	Forward Reverse	GCCTAACATGCTTCGAGATC TGATGTCTGGGTCTTGGTTC
IL-4	NM_000589	Forward Reverse	TCAACCCCCAGCTAGTTGTC TGTTCTTCGTTGCTGTGAGG
HPRT	NM_002046	Forward Reverse	GAGTCAACGGATTTGGTCGT GACAAGCTTCCCGTTCTCAG
GADPH	NM_001289745	Forward Reverse	ACAGTCAGCCGCATCTTCTT ACGACCAAATCCGTTGACTC

**Table 2:** Essential Amino acid composition of Sacha inchi protein products: SIF, SII, SIH5B, SIH10B, SIH15B, SIH20B, SIH30B and SIH60B.

Essential Aminoacid	SIF	SII	SIH5B	SIH10B	SIH15B	SIH20B	SI3H0B	SIH60B	FAO ^1,2^
His	14.03 ± 0.49	22.22 ± 0.15	48.33 ± 1.60	16.95 ± 1.02	15.11 ± 0.60	17.64 ± 2.14	15.99 ± 1.11	16.06 ± 0.06	15
Ile	25.45 ± 1.33	37.76 ± 0.29	35.37 ± 3.20	37.15 ± 2.06	31.65 ± 1.59	37.24 ± 4.31	37.55 ± 2.26	37.61 ± 0.45	30
Leu	41.69 ± 1.17	64.77 ± 0.35	53.41 ± 1.17	58.66 ± 3.52	48.22 ± 2.59	57.79 ± 7.70	50.61 ± 2.78	51.43 ± 0.83	59
Lys	29.12 ± 0.92	39.35 ± 0.14	35.42 ± 1.01	39.01 ± 2.54	32.02 ± 1.67	37.55 ± 4.76	34.23 ± 1.95	34.54 ± 0.60	45
Met + Cys	19.86 ± 3.56	25.16 ± 0.71	24.34 ± 1.68	24.01 ± 3.38	17.90 ± 1.69	25.62 ± 4.06	22.35 ± 2.77	18.45 ± 0.39	22
Met	5.40 ± 2.30	4.44 ± 0.07	8.83 ± 0.28	5.57 ± 1.69	3.50 ± 0.57	8.15 ± 1.37	8.20 ± 1.90	6.42 ± 0.31	16
Cys	14.46 ± 1.26	20.72 ± 0.64	15.51 ± 1.40	18.43 ± 1.69	14.36 ± 1.12	17.47 ± 2.69	14.15 ± 0.87	11.93 ± 0.08	6
Phe + Tyr	46.92 ± 1.27	72.29 ± 0.66	55.91 ± 1.62	61.85 ± 4.04	49.89 ± 2.47	64.79 ± 7.92	51.76 ± 2.76	52.06 ± 0.79	38
Thr	29.13 ± 0.47	44.29 ± 0.20	44.69 ± 0.58	42.13 ± 2.57	34.88 ± 2.00	40.80 ± 4.93	35.16 ± 1.80	35.40 ± 0.49	23
Trp	17.53 ± 0.00	24.29 ± 0.00	20.44 ± 0.01	19.25 ± 0.00	19.87 ± 0.00	19.36 ± 0.00	19.01 ± 0.00	19.49 ± 0.00	6
Val	30.74 ± 1.54	44.47 ± 0.22	32.01 ± 1.96	33.10 ± 1.42	29.39 ± 1.12	33.17 ± 2.74	32.48 ± 1.50	32.47 ± 0.30	39
Total essential aminoacids	274.33	399.76	374.26	356.11	296.79	359.58	321.49	315.86	277.00

Data is shown in % and ± SD of three determination. ^1^ FAO/WHO/UNU. Scoring pattern mg/g protein requirement in adults; ^2^ FAO and FINUT 2017. Dietary protein quality evaluation in human nutrition. FAO Food and Nutrition Paper NO. 92.

**Table 3:** Non-essential Amino acid composition of Sacha inchi protein products: SIF, SII, SIH5B, SIH10B, SIH15B, SIH20B, SIH30B and SIH60B.

Non-Essential Aminoacid	SIF	SII	SIH5B	SIH10B	SIH15B	SIH20B	SIH30B	SIH60B
Asp + Asn	69.15 ± 1.98	114.83 ± 1.02	138.36 ± 2.99	150.17 ± 8.65	124.28 ± 5.39	148.22 ± 19.63	126.39 ± 11.48	128.34 ± 1.92
Glu + Gln	78.37 ± 1.72	132.43 ± 1.05	132.23 ± 2.79	145.30 ± 8.41	118.86 ± 6.38	139.15 ± 17.32	122.12 ± 7.32	122.59 ± 1.53
Ser	39.43 ± 0.70	57.81 ± 0.22	48.33 ± 1.60	54.68 ± 3.41	44.09 ± 2.32	51.85 ± 6.38	43.01 ± 2.08	43.46 ± 0.72
Gly	63.38 ± 0.76	45.53 ± 1.37	33.38 ± 0.44	37.33 ± 2.31	30.35 ± 1.51	35.70 ± 4.34	31.52 ± 1.96	31.89 ± 0.36
Arg	61.84 ± 1.30	98.52 ± 0.46	83.39 ± 1.59	91.66 ± 5.59	75.32 ± 3.82	87.65 ± 3.20	79.03 ± 4.10	79.55 ± 1.30
Ala	21.79 ± 0.57	34.15 ± 0.23	31.33 ± 0.61	34.49 ± 2.15	27.75 ± 1.43	33.03 ± 2.25	28.89 ± 1.80	29.50 ± 0.47
Pro	1.00 ± 0.35	2.47 ± 0.20	29.39 ± 8.89	17.60 ± 2.33	29.10 ± 6.89	37.81 ± 6.05	28.46 ± 7.10	21.18 ± 1.16
Total non-essential aminoacids	334.96	485.74	496.41	531.23	449.75	533.41	459.42	456.51

Data is shown in % and ± SD of three determination.

**Table 4:** Antioxidant activity of Sacha inchi protein products.

Sample	DPPH (mg/mL)	Reducing Power (mg/mL)	Β-Carotene (mg/mL)
BHT	0.014 ± 0.004 ^a^	0.016 ± 0.002 ^a^	2.130 × 10^−9^ ± 0.030 × 10^−9a^
SII	146.120 ± 7.573 ^f^	2.417 ± 0.101 ^b^	1.224 ± 0.070 ^e^
SIH5B	66.143 ± 10.412 ^e^	2.178 ± 0.410 ^b^	0.855 ± 0.147 ^d^
SIH10B	28.865 ± 1.151 ^c^	2.200 ± 0.294 ^b^	0.260 ± 0.123 ^c^
SIH15B	28.351 ± 1.204 ^c^	2.280 ± 0.074 ^b^	0.242 ± 1.204 ^c^
SIH20B	16.670 ± 0.589 ^b^	2.486 ± 0.130 ^b^	4.180 × 10^−4^ ± 4.330 × 10^−4 b^
SIH30B	33.708 ± 2.643 ^c^	2.507 ± 0.308 ^b^	3.980 × 10^−4^ ± 1.470 × 10^−4b^
SIH60B	40.650 ± 3.713 ^d^	2.393 ± 0.185 ^b^	3.170 × 10^−3^ ± 1. 270 × 10^−4 b^

* Data represent the IC50 in different cell-free in vitro experiment. Values are presented as means ± SD of three determination, and those marked with different letters are significantly different (*p* < 0.05), as determined through one-way ANOVA, followed by a post hoc Tukey test.

**Table 5:** Characterization of the 20 highest relative abundance (determined by Area) peptide sequences identified in SIH20B based on in silico analyses.

Peptide	Area	Length	Molec. Weight	Charge ^1^	PI ^1^	Hydrophobicity ^1^	Steric Hindrance ^1^	Amphipathicity ^1^	Solubility ^2^	PeptideRanker ^3^	PreAIP ^4^	AnOxPePred ^5^
LATMMDDE	6.47 × 10^+7^	8	956.35	−3.0	0.59	−0.12	0.67	0.16	Good	0.1166	0.373	0.335
LGDECYGSG	6.47 × 10^+7^	9	956.35	−2.1	0.62	−0.06	0.65	0.14	Good	0.3430	0.383	0.457
LGDDSCYQ	6.47 × 10^+7^	8	956.35	−2.1	0.61	−0.20	0.66	0.16	Good	0.2995	0.386	0.398
GNGSLRML	3.39 × 10^+7^	8	863.42	1.0	10.84	−0.13	0.65	0.31	Poor	0.7512	0.471	0.403
GDGSLRLM	3.39 × 10^+7^	8	863.42	0.0	6.64	−0.14	0.65	0.31	Good	0.8027	0.446	0.365
TAGSLRME	3.39 × 10^+7^	8	863.42	0.0	6.55	0.29	0.62	0.47	Good	0.2015	0.438	0.377
GDGSLRML	3.39 × 10^+7^	8	863.42	0.0	6.64	−0.14	0.65	0.31	Good	0.8074	0.443	0.389
PGVLKAPPP	8.73 × 10^+6^	9	874.53	1.0	10.57	0.01	0.51	0.41	Poor	0.5328	0.360	0.435
PGVLKKHP	8.73 × 10^+6^	8	874.53	2.1	10.91	−0.19	0.50	1.10	Good	0.2851	0.399	0.410
LLSGAGVSFQ	4.11 × 10^+6^	10	977.52	0.0	3.70	0.16	0.61	0.12	Poor	0.2366	0.458	0.371
YLMLATPGL	4.11 × 10^+6^	9	977.53	0.0	3.70	0.23	0.57	0.00	Poor	0.4295	0.530	0.404
PQREVQSQ	1.92 × 10^+6^	8	970.48	0.0	7.31	−0.53	0.62	0.93	Good	0.0553	0.405	0.300
SAVSLGTHVL	1.77 × 10^+6^	10	982.54	0.1	7.54	0.15	0.53	0.14	Poor	0.2637	0.401	0.444
MVPLAQLVN	1.58 × 10^+6^	9	983.55	0.1	7.54	0.14	0.62	0.14	Poor	0.1605	0.396	0.379
MNACESC	1.07 × 10^+6^	7	870.27	−1.1	1.12	−0.13	0.64	0.18	Good	0.2798	0.422	0.353
AAGALKKFL	0.98 × 10^+6^	9	917.57	2.0	10.74	0.04	0.60	0.82	Good	0.7997	0.478	0.372
LGVKFKGGL	0.98 × 10^+6^	9	917.57	2.0	10.73	0.05	0.65	0.82	Good	0.6273	0.370	0.345
NGVLKKFL	0.98 × 10^+6^	8	917.57	2.0	10.68	−0.06	0.66	0.92	Good	0.4740	0.465	0.334
LFPPPGKA	0.84 × 10^+6^	8	825.47	1.0	10.12	0.03	0.52	0.46	Poor	0.7289	0.365	0.521
LLFGHATLE	0.75 × 10^+6^	9	999.54	−0.9	5.10	0.16	0.52	0.30	Poor	0.2124	0.473	0.498

^1^ Peptides were calculated from https://webs.iiitd.edu.in/raghava/toxinpred/design.php/ (accessed on 21 May 2024) where charge, isoelectric point (pI), hydrophobicity, steric hindrance, and amphipathicity were calculated. ^2^ Peptides were calculated from http://pepcalc.com/ (accessed on 21 May 2024), where the net charge at neutral pH was calculated. Meantime, this website estimates peptide solubility in pure water through the combined results of both charged residues number and the length of the peptide. ^3^ The likelihood of the peptides being bioactive was evaluated by PeptideRanker (http://bioware.ucd.ie/~compass/biowareweb). ^4^ Peptides are calculated from http://kurata14.bio.kyutech.ac.jp/PreAIP/ (accessed on 21 May 2024), where the probability of the peptide to exert anti-inflammatory effects is estimated based on various characteristics such as initiation, primary sequence, evolutionary, and structural information through a random forest classifier. ^5^ Peptides were subjected to calculation via https://services.healthtech.dtu.dk/services/AnOxPePred-1.0/ (accessed on 21 May 2024), a website used to predict the antioxidant properties (as quantified by free radical scavenging and ion chelation scores) of the peptides using a conventional neural network.

## Data Availability

The original contributions presented in the study are included in the article/Supplementary Material, further inquiries can be directed to the corresponding author.

## Supplementary Materials

The following supporting information can be downloaded at: https://www.mdpi.com/article/10.3390/foods13132045/s1, Table S1: Peptidome table SIH20B.
