# Supplementary material for: Sacha Inchi (Plukenetia volubilis L.) Protein Hydrolysate as a New Ingredient of Functional Foods
Source: Foods. 2024 Jun 27;13(13):2045. doi: 10.3390/foods13132045 (PMC11241537; doi:10.3390/foods13132045)
Supplement: Supplementary file 1 [file foods-13-02045-s001.zip › foods-3072501-supplementary.pdf]

Peptidome table SIH20B

Peptide	Length	Area	Mass
NVPGPGMGFLMWPY	14	85260000	1564,7207
EFDAPLALLMEGPY	14	85260000	1564,7483
LYPVGVDEPMSVPY	14	85260000	1564,7483
GVPM(+15,99)LTFGSPR	11	79350000	1176,5962
LATM(+15,99)M(+15,99)DDE	8	64740000	956,3467
LGDEC(+57,02)YGSG	9	64740000	956,3546
LGDDSC(+57,02)YQ	8	64740000	956,3546
AWLPTQSWKPVRAY	14	51960000	1701,8992
TLPTATNLKTL	11	46840000	1171,6812
FRGLVLSAPGR	11	46840000	1171,6826
TLGASGLVVLRS	12	46840000	1171,6924
LTPGANKLLVF	11	46840000	1171,6965
AAAFLVLSVGPK	12	46840000	1171,6965
NVFLVLGAVGAL	12	46840000	1171,6965
NVFLVLGKSPV	11	46840000	1171,6965
VNFLVLGSKVP	11	46840000	1171,6965
LTPKC(+57,02)VLKTL	10	46840000	1171,7
TLPKC(+57,02)VLTKL	10	46840000	1171,7
ALEPDSGPAAK	11	38280000	1054,5295
ALEPDNGVLQ	10	38280000	1054,5295
LAEPDASAPGK	11	38280000	1054,5295
ALEPDQAAGAL	11	38280000	1054,5295
ALEPDNGVGAL	11	38280000	1054,5295
ALEPDNRGAL	10	38280000	1054,5408
PSEPNAAKNK	10	38280000	1054,5408
RADLFVPEVGRVS	13	38190000	1443,7834
RADLFVPEVGRVS	13	38190000	1443,7834
HNLNNPSDSDLFNPR	15	36280000	1738,8022
GDGSLRPY	8	33940000	863,4137
GDGSLRLM(+15,99)	8	33940000	863,4171
TAGSLRME	8	33940000	863,4171
GDGSLRLM(+15,99)	8	33940000	863,4171
GDGSLRM(+15,99)L	8	33940000	863,4171
GN(+,98)GSLRM(+15,99)L	8	33940000	863,4171
GADVRLKFGTQ	11	27310000	1190,6406
MGSFAANGSFLLKDMFGPPFPGM	23	25760000	2418,1208
VSLYPPGAMM(+15,99)GVLM	14	25270000	1480,7129
TAVFPTPAMM(+15,99)GVLM	14	25270000	1480,7129
LTGFWYHNTVLM	12	25270000	1480,7173
LLN(+,98)TSNSLGDALDQNSEAMGNLLA	24	22130000	2461,1641
RESEQERENLEEEAQR	16	17510000	2030,9253
RSSAVEMGPEPDSGEEAKR	19	17510000	2030,9326
RSQEEEREQVGGSGEAQR	18	17510000	2030,9365
LGVAGASGSGEVSF	14	13960000	1236,5986
ASNKGLEEGVSF	12	13960000	1236,5986
AWLPTQSGEKESLTR	15	13340000	1701,8687
AWLPTPVPKDALSPGGP	17	13340000	1701,9089

GAVSLEDLNRL	11	12680000	1185,6353
HVEALTKSSLGC(+57,02)QHDMF	17	10790000	1958,8979
VHEALTKSSLGPC(+57,02)DDALM(+15,99)	18	10790000	1958,9077
VGTEGVDVSVFYV	13	9732000	1369,6765
NLPM(+15,99)VVAYL	9	9272000	1034,5471
NLPKGHPVSS	10	9272000	1034,5508
GGLPHKGGALE	11	9272000	1034,5508
NLPM(+15,99)LRYL	8	9272000	1034,5583
PGVLKAPPP	9	8726000	874,5276
PGVLKKHP	8	8726000	874,5388
PGQERFQGDHHQKAAL	16	8257000	1817,8921
DVFVPEVRGLW	11	7803000	1315,6924
VDFVPEVAAAVSL	13	7803000	1315,7024
LQGGDQNHRDFFLA	14	7008000	1616,7695
NQLDQNHRDFFLA	13	7008000	1616,7695
VVFN(+,98)DTANKNNQLDPPGAMAGPC(+57,02)RVA	26	6737000	2756,301
NEEWFFPGLVN	11	6611000	1350,6245
PQNFVVLTK	9	6351000	1044,5967
LQDNYPLVTE	10	5677000	1190,5818
MLKVDFSPASP	11	5677000	1190,6006
VGGAYSAGPANYV	13	5132000	1224,5774
VNANRDFQYV	10	5132000	1224,5886
VYLLLHSPVF	10	5038000	1186,675
LAEAPSTVPNG	11	4962000	1054,5295
LAEPNAQAGGK	11	4962000	1054,5408
MFALYGDGSLLM(+15,99)R	13	4922000	1488,7104
LMKGKKC(+57,02)VL	9	4684000	1075,6245
FYELLFGSWLF	11	4633000	1420,7068
VPLHFSGQAAFVK	13	4563000	1399,7612
VPLPGVTRSPALHG	14	4563000	1399,7935
QEGGLVGWPF	10	4439000	1088,5291
LLSGAGVSFQ	10	4113000	977,5182
YLMLATPGL	9	4113000	977,5256
RPM(+15,99)LTFGSPR	10	3894000	1176,6074
VVFN(+,98)DTRWYVK	11	3623000	1426,7244
LDVKQ(+,98)SN(+,98)ASTPQ	12	3608000	1288,6147
LVTPGFLESELTQGKLL	17	3524000	1844,0295
LVTPGFLESELTKGAGLL	18	3524000	1844,0295
YYGKAVLNVEAAAQ	14	3495000	1495,7671
SVLPTGASYALGTPNAL	17	3306000	1630,8567
SVLPTQSWKHVNHV	14	3306000	1630,8579
VVN(+,98)NFFDDFLRP	12	3225000	1482,7144
SGGVNDPFNGVEPM(+15,99)PS	16	3202000	1618,6934
QTLLHVEKGHGVTK	14	3119000	1545,8628
NVHAGRLVGPN	11	2806000	1132,6101
VSEHN(+,98)LDLVAREP	13	2766000	1478,7366
SVEHN(+,98)LDLVAREP	13	2766000	1478,7366
LGFPPTKFTPLM	12	2602000	1347,7261
MLNFVAAGDTSLWQV	15	2442000	1650,8076

GDDSHGEGGLL	11	2391000	1055,4519
SDGKEASYSSSAALGSFVQC(+57,02)VVQ	23	2302000	2376,0903
DTLAHRLEQGV	11	2276000	1237,6414
LGVRGGLMFNL	11	2256000	1175,6484
AGPPSLLPSPPV	12	2137000	1130,6335
YPALTVPAAHVWPY	14	2122000	1583,8137
WLTGGGVDLPC(+57,02)AETF	15	2002000	1621,7446
FWPYHGKENGAKPASTW	17	1971000	1974,9377
PQREVQSQ	8	1917000	970,4832
NFLN(+,98)SN(+,98)PSNNLKM(+15,99)N	14	1873000	1623,72
NFLN(+,98)SNQGNNLAGFN	15	1873000	1623,7278
LLASVSTLMVHV	12	1871000	1268,7163
VVVSKMGKSVHV	12	1871000	1268,7275
MLRLLTTSVHV	11	1871000	1268,7275
VVLDVKGSDAAGGLDLL	17	1865000	1640,8984
M(+15,99)PQGAGDDGDGVQ	13	1853000	1261,488
SAVSLGTHVL	10	1767000	982,5447
GVADEQLNLR	10	1711000	1113,5779
LDADDPFDRSPFPS	14	1699000	1577,6997
TGPGKEPHTGPHGGHPAKGSA	21	1629000	1975,9612
EGSVLDAGWFL	11	1585000	1192,5764
MVPLAQLVN	9	1582000	983,5474
SGAGTAFAPNAVV	13	1536000	1160,5825
APSGSLGPMFVV	12	1536000	1160,5898
FLLTLFGSASHLGFLT	16	1528000	1722,9343
NKLVSGVEPLHLGFLT	16	1528000	1722,9668
GGNAAKMAAKN	11	1516000	1031,5182
LRPHGNGNAP	10	1516000	1031,5261
QLNNPSN(+,98)SDLFNPR	14	1431000	1615,759
VTTVATVMAM(+15,99)VQLL	14	1404000	1491,8042
PKAGVNGLDDASGS	14	1326000	1286,6101
ESSSATLPLSH	11	1323000	1127,5459
SSNSNLRLL	9	1288000	1002,5458
LTADALPTKSEGKVPRAY	18	1197000	1916,0366
NSN(+,98)NQLDLNPR	11	1191000	1284,6057
LSVSESGGC(+57,02)GPA	12	1187000	1119,4866
SHSVPSM(+15,99)GTVDVK	13	1178000	1358,6499
QAVRLPHSVNLN	12	1167000	1346,7419
LTAVTSPVTNGKL	13	1153000	1299,7397
LVEAQAPETVL	11	1142000	1168,634
LVDVDGTGLALP	12	1142000	1168,634
LLFWPYHGKENKSGHFKN	18	1129000	2201,1169
NGPEGERPPSGGG	13	1073000	1209,5374
MNAC(+57,02)ESC(+57,02)	7	1068000	870,267
AWLPTYVLSKSALW	14	1063000	1633,8867
RPGLNGPSPGRG	12	1008000	1163,616
LN(+,98)KGLPHWNLN	11	998300	1305,6829
VFKDFVVVGDMK	12	987800	1382,7268
LSVAKMVHGVVM(+15,99)P	13	987800	1382,7415

VFPVGHVVSALAST	14	987800	1382,7559
AAGALKKFL	9	979800	917,5698
NGVLKKFL	8	979800	917,5698
LGVKFKGGL	9	979800	917,5698
EHEMGM(+15,99)GLDRSYVF	14	925600	1685,7178
TALPDAFPAGA	11	922600	1029,5131
FMMASALPYNDLSH	14	903200	1595,7112
NAGSTGPSTPGQ	12	877900	1072,4785
LMEEGM(+15,99)M(+15,99)PGLNVK	13	852700	1479,6772
QAWLPTALEFLHTQP	15	843800	1750,9043
LFPPPGKA	8	840700	825,4748
C(+57,02)VHKSTLMWM(+15,99)L	11	831800	1420,6665
LM(+15,99)WGGSVGPQ	10	831500	1046,4856
DKEVAEKPSM(+15,99)P	11	817500	1245,5911
QLSAEKAVLPAGAH	14	816400	1390,7568
LM(+15,99)TLPDLAGWLTPQ	14	791500	1570,8064
LLPFSSGGSRVPVYLVQ	17	784100	1818,0039
LMKVKMKVVH	10	758200	1211,7246
LLFGHATLE	9	754200	999,5389
PNVSVSFK	8	733300	876,4705
GVSPELSSN	9	711800	888,4189
GGNSKELGQ	9	711800	888,4301
QYVNGPKLLY	10	688800	1193,6445
SSAQPTTLKML	11	688700	1175,6221
NVATLTLLGPN	11	685500	1111,6238
YSNSPKLLYK	10	670800	1211,655
VEEGKVEAH	9	660400	996,4876
LESDANPSP	9	633200	928,4138
VSPVGPLVGPE	11	630500	1049,5757
DGGANSPVTYGGTA	14	612800	1265,5525
MLGRLTAAATPQ	12	583700	1228,6597
SVSKPVGVL	9	577800	884,5331
LDAQGRTKA	9	576700	958,5196
KGEAAALLQ(+,98)KENEYVK	16	568100	1790,9414
KTSLSQMALALEPVVL	16	553900	1698,959
PLAVGALVGPNAF	13	546800	1224,6865
KTHQDAKLS	9	539600	1026,5458
LGYEAQDGSAQ	11	530900	1137,4939
ANQNNQLGGHSPSNKPS	17	528600	1748,8191
DGDSLSVNLVGAL	13	528200	1258,6404
NPSPYGTLPLT	11	507400	1158,592
VAVM(+15,99)VLLTGAVP	12	504100	1184,6838
LKGLLWDEHGNSGLELW	17	500900	1965,9949
VFPGFATADAGQL	13	499600	1292,6401
PSGLPPTGHV	10	496500	960,5029
KVFSGPPT	8	478800	831,449
VGGVQFPPV	9	478600	898,4912
VVDENGNSVFDGSWKVP	17	477300	1847,8689
ELGGNGGYNSLGK	13	472600	1264,6047

VGSLEPTPAPAT	12	471200	1138,5869
ESDAPSQTLL	10	465700	1059,5083
LGTGAELLHLPQ	12	436400	1247,6873
SLGLQPDVALLPSYSNAPQLF	21	432700	2229,168
VPNRGTLPVL	10	428700	1064,6343
TTAFLKGLGQ	10	419900	1034,5759
FGLAVVHPVP	10	419900	1034,5913
LGSVLAGRGL	10	399900	941,5658
YVVFVVPPPPAA	12	397700	1254,7012
DKLALKKVAC(+57,02)SLL	13	396400	1457,864
GFLLNGEWTPW	11	381700	1318,6345
KMVSLVGAPGGNYLPA	16	365800	1572,8333
MWPTAAALL	9	364400	972,5103
DKEFLPGPMTLK	12	359100	1374,7217
YAEKTPSDSDLFNPR	15	356100	1738,8162
YKVVLDVKEDSALNGELL	18	344500	2004,0779
NFPVLPAAPN	10	342600	1038,5498
LEPDDPAVE	9	331600	983,4447
KTGVLRFRL	9	331200	1088,6819
YYKVVL	6	329800	783,4531
DNM(+15,99)TPC(+57,02)Y	7	304800	915,3102
NLGTLGVTPV	10	302800	969,5495
PGVALRVPGP	10	292400	961,5709
KVSHAYKADSRL	12	271500	1373,7415
EGTSLWGN(+,98)PK	10	264100	1088,5139
MVLPLNAMP	9	255900	984,5136
YVVFVVM(+15,99)RKV	10	255200	1254,7158
VTLVGLSLPV	10	233900	996,6219
VTLTLLAAPV	10	233900	996,6219
EGLTNGGKM(+15,99)SMVK	13	233500	1366,6584
NPLLALVGPN	10	2,30E+05	1006,5811
QTGVLAYKK	9	2,30E+05	1006,5811
GPVVRLVGPGG	11	2,30E+05	1006,5923
VVGPRLVGPN	10	2,30E+05	1006,5923
NEAAVYPVSF	10	215900	1095,5237
VPLPHSVGKPA	11	195500	1100,6343
QVVPDGPPSHNFL	13	186800	1405,699
FFQ(+,98)LQNLASAHE	12	1,78E+05	1404,6672
FM(+15,99)ELKNTQHEL	11	1,78E+05	1404,6707
ALEPDNVGLQ	10	177200	1054,5295
NPPELNKDGLGHE	13	1,54E+05	1418,679
NHGQ(+,98)LKNNSVPPN	13	1,54E+05	1418,6902
LKDGADVLK	9	1,38E+05	957,5494
PQNFLVLK	8	1,38E+05	957,5647
PKNM(+15,99)LVLK	8	1,38E+05	957,5681
ADLALLPGALALNA	14	1,36E+05	1321,7605
ADLALLPQLALNA	13	1,36E+05	1321,7605
TTVVGKSLDGGPRA	14	1,29E+05	1356,7361
LNDVFVPEVGRL	12	1,29E+05	1356,74

LELDNYSNNKL	11	1,14E+05	1321,6514
NQLDNYSDLKL	11	1,14E+05	1321,6514
LNGVVVDHGAPL	12	110900	1189,6455
DYLHALLMGPEY	12	1,10E+05	1420,6697
AVRLPHEGNLN	11	1,02E+05	1218,647
LGRLPHDANLN	11	1,02E+05	1218,647
AVRLPHWNLN	10	1,02E+05	1218,6621
LGRLPHWNLN	10	1,02E+05	1218,6621
ANAAAVVPQLALPA	14	8,44E+04	1304,7451
ADLALLPPVVLNA	13	8,44E+04	1304,7703
ELKGVLVGPN	10	7,99E+04	1024,5917
SVAGVLLVGPN	11	7,99E+04	1024,5917
ELKAVVVGPN	10	7,99E+04	1024,5917
ELGVKLVGPN	10	7,99E+04	1024,5917
EKGVLLVGPN	10	7,99E+04	1024,5917
EGKVLLVGPN	10	7,99E+04	1024,5917
ELKRLVGPN	9	7,99E+04	1024,6029
Q(+,98)LKRLVGPN	9	7,99E+04	1024,6029
KGGKVGLVGNP	11	7,99E+04	1024,6029
NKKVVAVGPN	10	7,99E+04	1024,6029
QTGVLRPAAL	10	7,99E+04	1024,6029
KGGKRLVGPN	10	7,99E+04	1024,6141
FHADNYPRRF	10	7,70E+04	1321,6316
LWPDKFSMGLE	11	7,70E+04	1321,6377
ENAVGATKVAHPE	13	7,70E+04	1321,6626
NEAVVHDNAGALL	13	7,70E+04	1321,6626
SSAASANTLLNRP	13	7,42E+04	1300,6736
SSASAANTLLNRP	13	7,42E+04	1300,6736
GYDPRTLLNPGV	12	7,42E+04	1300,6775
LGPETRRTVL	10	7,37E+04	1140,6614
ALDGTDVGEGPGYDDL	16	7,24E+04	1592,6841
QVEHELEFGYDDL	13	7,24E+04	1592,6995
LNEQGPPEFGYDDL	14	7,24E+04	1592,6995
FFVDNLQ(+,98)FGYDDL	13	7,24E+04	1592,7034
FMLAMSFTLMGPEY	14	6,65E+04	1636,7339
FMADAQLYKEASYT	14	6,65E+04	1636,7444
ESDAAGVDALLMWPY	15	6,65E+04	1636,7444
ASAPTAPVMAYLGEEM	16	6,65E+04	1636,7476
WSVGSHAFGMLWM(+15,99)L	14	6,65E+04	1636,7529
LHVTRPPRTQ	10	6,60E+04	1203,6836
HGKDNQKWSLR	11	6,50E+04	1367,7058
PSLALAAPGPP	11	6,17E+04	989,5545
PGVVRLVGGH	10	6,17E+04	989,577
NSKEARRLKFN	11	5,55E+04	1361,7527
LNNPSN(+,98)SDLFNPR	13	5,50E+04	1487,7004
LNNPSDSDLFNRP	13	5,50E+04	1487,7004
SGVGPKVC(+57,02)L	9	5,34E+04	915,4847
PETSRTLL	8	5,34E+04	915,5025
HGKENQKWSLR	11	5,23E+04	1381,7214

M(+15,99)FQ(+,98)LKNATGVHE	12	5,15E+04	1390,655
FFEVGFQPGAVPP	13	5,15E+04	1390,6921
PGSRHTSPGAYDPVSS	16	5,14E+04	1613,7434
PGHSRGGQDSKDNLF	15	5,14E+04	1613,7546
PGHSRNQDSSVGNLF	15	5,14E+04	1613,7546
PGHSRGGQDSAGAASLF	17	5,14E+04	1613,7546
WLYNNGRTPLVL	12	4,93E+04	1444,7827
DVKQ(+,98)SN(+,98)AAPSGTLL	14	4,85E+04	1401,6987
DVKESN(+,98)AARADLL	13	4,85E+04	1401,71
DVKQSTNTGTPAAL	14	4,85E+04	1401,71
QAWLPTQSEGKPVQVY	16	4,65E+04	1829,9312
KAGELPTQSWKPVGVAY	17	4,65E+04	1829,9675
LLGLPSYSNAPQLF	14	4,62E+04	1518,8081
GVSTGPGQEFVGQG	14	4,60E+04	1318,6152
RSTGPGQEFRQG	12	4,60E+04	1318,6377
KTPHVTVGPPRTQ	13	4,49E+04	1416,7837
EVANELDSPGYDDL	14	4,46E+04	1535,6626
LGEAPNPEFGYDDL	14	4,46E+04	1535,678
FFVDGLEFGYDDL	13	4,46E+04	1535,6819
LMYTGLQ(+,98)FGYDDL	13	4,46E+04	1535,6853
LYMTGLQ(+,98)FGYDDL	13	4,46E+04	1535,6853
PGVVVGLVGHG	11	4,45E+04	989,5658
GFKGANVKVA	10	4,45E+04	989,5658
VGTKPENDYRGLLV	14	4,42E+04	1559,8308
VLRGGENDVLFATAV	15	4,42E+04	1559,8308
TVVDSGPAGSVFDGQ	15	4,40E+04	1434,6626
VLSNSPEGSVFDGQ	14	4,40E+04	1434,6626
LVNSSPGESVFDGQ	14	4,40E+04	1434,6626
DSGLGKNQAVFDAN	14	4,40E+04	1434,6738
LVNRDADSVFN(+,98)GQ	13	4,40E+04	1434,6738
LVNDRGESVFDGQ	13	4,40E+04	1434,6738
NFGFVSADVTPGPQ	14	4,40E+04	1434,678
LVLSC(+57,02)NPSVFDGQ	13	4,40E+04	1434,6814
LVSMAGPQSVFDGQ	14	4,40E+04	1434,6814
LVMAGSGPASVFDGQ	15	4,40E+04	1434,6814
LVSC(+57,02)ANLLVM(+15,99)DGQ	13	4,40E+04	1434,6846
LVM(+15,99)GFSYGVFDGK	13	4,40E+04	1434,6853
LVHDYRSVFDGQ	12	4,40E+04	1434,6892
FVYVVGKTVLM	11	4,28E+04	1254,7046
M(+15,99)VPVVVGPVVLM	12	4,28E+04	1254,708
VGWYVK	6	4,04E+04	750,4064
RDHPVK	6	4,04E+04	750,4136
EGLYNN(+,98)RGTPLVL	13	3,82E+04	1445,7515
M(+15,99)VSAKQSGVAANM	13	3,73E+04	1308,6167
VFPPTGSAFAANM	13	3,73E+04	1308,6172
VM(+15,99)PVVPQHAANM	12	3,73E+04	1308,6318
NLPLANAVAH	10	3,64E+04	1018,556
VGAPLQGGAHL	11	3,64E+04	1018,556
NLPMLRYL	8	3,64E+04	1018,5634

KGSRLMVMAGLPE	13	3,64E+04	1387,7314
PEAALPTQSWAGPVRAY	17	3,63E+04	1812,9158
MTVPDLQQLW	10	3,61E+04	1229,6113
QSRGMPKQLGE	11	3,61E+04	1229,6187
C(+57,02)APGKVVLPHADHAN	15	3,54E+04	1584,783
C(+57,02)AHTPPPLGKVSHAN	15	3,54E+04	1584,783
NLTGAAVDAFVSVHAN	16	3,54E+04	1584,7896
GGEAAAVRLPHWHAN	15	3,54E+04	1584,791
GGFALAFNLAPSVHAN	16	3,54E+04	1584,8049
SPGGDTYNGTAGE	13	3,52E+04	1224,4895
DVKESDAARGELLMWPY	17	3,48E+04	1978,9458
VDKEDSAVGAWLLMWPY	17	3,48E+04	1978,95
YVNGPKLLY	9	3,45E+04	1065,5859
LNNPSDSNNFNPR	13	3,45E+04	1487,6753
NLNPSDNSQGLSKD	14	3,45E+04	1487,6853
QVNPSN(+,98)SDLGNTGGK	15	3,45E+04	1487,6853
LNNPSN(+,98)SN(+,98)LFNGPV	14	3,45E+04	1487,6892
LNNPSDMATAEPKT	14	3,45E+04	1487,6926
LNNPSDSMPALASTA	15	3,45E+04	1487,6926
LNNPSN(+,98)SDLFNRP	13	3,45E+04	1487,7004
LNNPSDSDLFNPR	13	3,45E+04	1487,7004
VQNPSN(+,98)SDLFPRN	13	3,45E+04	1487,7004
LNNPSVHHGM(+15,99)NRP	13	3,45E+04	1487,7051
LNNPSDAAHNSVHL	14	3,45E+04	1487,7117
LNNPAMSDFQVPR	13	3,45E+04	1487,719
LNNPSAMDLFNRP	13	3,45E+04	1487,719
NNGRTPLVL	9	3,44E+04	982,556
SSGWVGC(+57,02)C(+57,02)MGMDERGSSGQ	19	3,43E+04	2046,7651
VVN(+,98)ENGNSVLGLGPQGVPHSTA	22	3,38E+04	2146,0654
MDDYGFQ(+,98)LSVTM(+15,99)L	13	3,27E+04	1535,6523
ENQHAVEFGYDDL	13	3,27E+04	1535,6528
FVDAFVQ(+,98)FGDMM(+15,99)L	13	3,27E+04	1535,6677
MVMYGFQ(+,98)DAPTYL	13	3,27E+04	1535,6677
VFN(+,98)YGPEMHQSNL	13	3,27E+04	1535,6714
FVDYGFQ(+,98)LDGDFL	13	3,27E+04	1535,6819
FVDYGFELDGDFL	13	3,27E+04	1535,6819
LMAYVTEFGYDDL	13	3,27E+04	1535,6853
ENGKHLEFGYDDL	13	3,27E+04	1535,6892
LMLAPQKGLAL	11	3,23E+04	1153,6892
MMVFVVVSVVK	11	3,19E+04	1236,6975
VFPPHAKVVLM	11	3,19E+04	1236,7053
FVPPAHKVVLM	11	3,19E+04	1236,7053
LGRLPHGNELN	11	3,12E+04	1218,647
AVRLPHDANLN	11	3,12E+04	1218,647
AVRLPHGQDLN	11	3,12E+04	1218,647
AVGVLPHWNLN	11	3,12E+04	1218,6509
AVVGLPHWNLN	11	3,12E+04	1218,6509
AVLRPHWNLN	10	3,12E+04	1218,6621
LDFTAGETKV	10	3,12E+04	1079,5498

LDFFPTVLE	9	3,12E+04	1079,554
LDFNVVAVGF	10	3,12E+04	1079,5652
GLPLLKADKVQAQ	13	3,10E+04	1379,8135
AVPLLKADKVQAAG	14	3,10E+04	1379,8135
GQKSGFVKLL	10	3,04E+04	1075,6389
GQKGSFVKLL	10	3,04E+04	1075,6389
KTGGSNTHLER	11	3,01E+04	1198,6055
NLDAMRLVGPN	11	3,01E+04	1198,6128
GDDSLWNLR	9	2,99E+04	1074,5093
LLGNRGDLL	9	2,97E+04	969,5607
ADLALPLLALNA	12	2,95E+04	1193,7019
AAPKEEQEERDLEVGQ	16	2,86E+04	1826,8645
TAVPERTRTVL	11	2,84E+04	1241,7092
QEGEGEC(+57,02)E	8	2,83E+04	936,313
LWSSASGAGDK	11	2,80E+04	1077,509
LWPSYGGAEV	10	2,80E+04	1077,5132
PGHSRGGQDSKDN	13	2,80E+04	1353,6021
PGHSRGGQDSSRN	13	2,80E+04	1353,6133
NAVRLPHWNLN	11	2,73E+04	1332,7051
MDMFGFQ(+,98)LVMVM(+15,99)Q	13	2,71E+04	1592,6748
MVMYGFELGTFANN	14	2,71E+04	1592,7004
MQVTYLEFGYDDL	13	2,71E+04	1592,7068
M(+15,99)VDYGFELNLTFE	13	2,71E+04	1592,7068
C(+57,02)LTAKHLPHVSNLN	14	2,69E+04	1602,8301
C(+57,02)LKATHLPHVSNLN	14	2,69E+04	1602,8301
NQGSPLGNGVHDK	13	2,69E+04	1321,6375
NQLPPGTGHGMTL	13	2,69E+04	1321,6448
LQ(+,98)LDMYPDLTL	11	2,69E+04	1321,6475
NGALNPPTPLAEE	13	2,69E+04	1321,6514
NQLNNPGPSAPTL	13	2,69E+04	1321,6626
DQNHVFGDFLA	11	2,63E+04	1261,5728
DQNHRDFFLA	10	2,63E+04	1261,584
SN(+,98)AKPVRAY	9	2,62E+04	1005,5243
SWKPVRAY	8	2,62E+04	1005,5396
LGEETYAHFESDSM(+15,99)GPLDGFAETF	24	2,56E+04	2665,1165
VAEETYGM(+15,99)PAQTGDM(+15,99)GPLDGFAETF	25	2,56E+04	2665,1199
KAWLPTQSWAGPVHPR	16	2,53E+04	1829,969
KADALPTAGSWKVPHPGV	18	2,53E+04	1829,9788
WLPTQSWKPVRAY	13	2,52E+04	1630,8621
WLPTQSWKPVHPR	13	2,52E+04	1630,8733
LMLLFGFLAL	10	2,49E+04	1136,6667
LMALPVRGPAL	11	2,49E+04	1136,6738
NEEWFFPGLGRQ	12	2,48E+04	1478,6941
LNDTPEALMLKL	12	2,47E+04	1356,7322
AVLLPAYSNSPKLLY	15	2,41E+04	1647,9236
LGLLPAYSNAALGLLY	16	2,41E+04	1647,9236
LGLLPAYSNSPKLLY	15	2,41E+04	1647,9236
TPADQPVRAY	10	2,39E+04	1116,5564
EPASQPVRAY	10	2,39E+04	1116,5564

VFVPEVPTK	9	2,38E+04	1014,575
VFVPANGKAL	10	2,38E+04	1014,5862
LVVVGPPTPFVGP	13	2,38E+04	1277,7383
LVVVPGTPPFRP	12	2,38E+04	1277,7495
C(+57,02)AVVVTPEEVVAN	13	2,35E+04	1385,686
FMEGM(+15,99)PDY	8	2,34E+04	1004,3619
DFKLAHSAK	9	2,32E+04	1015,545
Q(+,98)PAVLVKFN	9	2,32E+04	1015,5702
MVLGGGGLFV	10	2,31E+04	948,5103
TLSATVPKDKL	11	2,28E+04	1171,6812
TLPGAMKLKTL	11	2,28E+04	1171,7
LPAARLVGPN(+,98)GL	12	2,22E+04	1177,6819
WLYNNGRTPLVLVQ	14	2,22E+04	1671,9097
WLSASVLM(+15,99)TPLVLVQ	15	2,22E+04	1671,927
TKPNTPGGVL	10	2,21E+04	982,5447
MVPLTVLNP	9	2,21E+04	982,5521
MPLVTPNVL	9	2,21E+04	982,5521
LKPHYNVL	8	2,21E+04	982,56
YVDGPKLLY	9	2,16E+04	1066,5698
MSLKGGPNKLSAPM	14	2,14E+04	1429,7422
KDNVGDQVTVL	11	2,14E+04	1186,6194
KMAQTPGAATVL	12	2,14E+04	1186,6379
SSAYWVGKTGPGDK	14	2,13E+04	1451,7043
SSVEGGGKRWYVQ	13	2,13E+04	1451,7156
LNDELQAGVKL	11	2,11E+04	1198,6558
NLDEQLARKL	10	2,11E+04	1198,667
ATVGNGVEGATVL	13	2,07E+04	1186,6194
KGSSAPGSGLDVL	13	2,07E+04	1186,6194
MVTVHKDKVM	10	2,07E+04	1186,6201
MVLSEDPGVLK	11	2,07E+04	1186,6267
ATVPESTAVTVL	12	2,07E+04	1186,6445
M(+15,99)GLM(+15,99)FAQHGS	10	2,06E+04	1109,4634
ATSGKLTGTKL	11	2,06E+04	1075,6238
MLKGKKVLC(+57,02)	9	2,06E+04	1075,6245
KGKMC(+57,02)VKLL	9	2,06E+04	1075,6245
LMKKMKGQL	9	2,06E+04	1075,6245
MLKKGVKC(+57,02)L	9	2,06E+04	1075,6245
MLKGKKAMGL	10	2,06E+04	1075,6245
LGGFETVKLL	10	2,06E+04	1075,6277
PFKGKKSTAL	10	2,06E+04	1075,6389
PFKPLAPPPL	10	2,06E+04	1075,6428
PTAGSWKPVRAY	12	2,03E+04	1331,6985
PTQSWKPVRHP	11	2,03E+04	1331,7097
LVNDRGESVFN(+,98)GQ	13	2,02E+04	1434,6738
YEGSGPESSGSSGC(+57,02)DGVGSSGQ	22	2,02E+04	2046,7708
YQ(+,98)GGDPTSSGSSGC(+57,02)DRGSGSAG	22	2,02E+04	2046,782
YEGSGWGEGSSGSSPGMGSSGQ	22	2,02E+04	2046,7861
GDDSNAGAPGPE	12	2,01E+04	1085,426
NAVGVLPHWNLN	12	1,94E+04	1332,6938

LPTQSWKPVVGAY	13	1,93E+04	1444,7715
LPTQSWKPVRAY	12	1,93E+04	1444,7827
LRGLSWQFDVKP	12	1,93E+04	1444,7827
MLSAAANNAPT	11	1,91E+04	1059,502
ESDAPNTTLL	10	1,91E+04	1059,5083
ESDAPSTQLL	10	1,91E+04	1059,5083
ESDAPSQTLL	10	1,91E+04	1059,5083
Q(+,98)SDAASASPLL	11	1,91E+04	1059,5083
ESDAAAVDGLL	11	1,91E+04	1059,5083
ESN(+,98)AGGKGNLL	11	1,91E+04	1059,5195
ESDARQELL	9	1,91E+04	1059,5195
NNAVGVLPHWNLN	13	1,87E+04	1446,7368
MPAVRLPHWNLN	12	1,87E+04	1446,7554
ADVGANARLTR	11	1,84E+04	1142,6155
SWKPVYVQ	8	1,83E+04	1005,5283
SWKPTAGFL	9	1,83E+04	1005,5283
YFVADLLEGRAAHWPY	16	1,80E+04	1906,9365
VVDAFDNFFVGRP	13	1,79E+04	1481,7302
VVDNFFNDFLPR	12	1,79E+04	1481,7302
LQQPTAGSWKM(+15,99)LTQP	15	1,78E+04	1700,8555
EKLSNAGTGSLSTSGPVP	18	1,78E+04	1700,8582
LPVGAALVGPNGL	13	1,75E+04	1176,6865
LPAARLVGPGGGL	13	1,75E+04	1176,6978
LEGLFPEDC(+57,02)YGQN	13	1,74E+04	1540,6504
LEGLFPDNM(+15,99)TM(+15,99)NQ	13	1,74E+04	1540,6538
LWLM(+15,99)PGEAM(+15,99)YGNQ	13	1,74E+04	1540,6689
FVPPTGPVVVLM	12	1,74E+04	1254,7046
FVPPTPRVVLM	11	1,74E+04	1254,7158
KNDKAGVLKFRGQ	13	1,73E+04	1459,8259
KNDAKRLKFGRQ	12	1,73E+04	1459,8372
YVVGPGSSLPQVPK	14	1,73E+04	1426,782
DGVFPPVVGVVVLM	14	1,73E+04	1426,7893
RGAREEEHPYPR	12	1,73E+04	1495,728
NPTAAGHPGSQLL	13	1,70E+04	1261,6414
LM(+15,99)PGTEMFGPM(+15,99)HVAPETM(+15,99)	18	1,68E+04	2022,856
NVHGLNNVDSAANGFNVPG	19	1,67E+04	1894,8921
SLANFLDSDSPNNLNFK	17	1,67E+04	1894,906
KASNVKAESVVSF	13	1,64E+04	1364,73
VSLEDARGVL	10	1,64E+04	1057,5767
VSLEDLNRL	9	1,64E+04	1057,5767
LDGNVGLPHWHL	12	1,63E+04	1356,6938
NDAVRLPHWHL	11	1,63E+04	1356,7051
NAVAAKPHVSHLN	13	1,63E+04	1356,7263
VRLPHWNLN	9	1,62E+04	1147,625
GDDSNYDGMY	10	1,62E+04	1135,3765
GDDYM(+15,99)DELY	9	1,62E+04	1135,4016
LSAMGATFGHS	11	1,60E+04	1077,4912
TKLGSFSLGPQ	11	1,60E+04	1133,6082
Q(+,98)LGGRPALLNAPPELTFGSPR	21	1,57E+04	2191,1748

LGLLPAYSNSVAPALYLVQ	19	1,57E+04	1988,0981
LGLLPAYSGTSSSLVLALVQ	20	1,57E+04	1988,1194
LGLLPAYSNSPKLLYLVQ	18	1,57E+04	1988,1345
EKGVLLVGPN	10	1,55E+04	1024,5917
EKGVLLVGNP	10	1,55E+04	1024,5917
ELKGVLVGNP	10	1,55E+04	1024,5917
NKKRLVGPN	9	1,55E+04	1024,6141
QLQVGLVGPN	10	1,54E+04	1023,5713
NVRVGLVGPN	10	1,54E+04	1023,5825
NVGVRLVGPN	10	1,54E+04	1023,5825
ALKSMPEEVVAH	12	1,54E+04	1309,6699
GGTKGDGYVAGPV	13	1,54E+04	1176,5774
LVSSSTAAGNTGL	13	1,54E+04	1176,5986
SKGNETTTNLL	11	1,54E+04	1176,5986
HVLFDFGA	8	1,52E+04	904,4443
HVGDFFLA	8	1,52E+04	904,4443
AMFGAALQP	9	1,52E+04	904,4476
FVPPTVYNVEL	11	1,52E+04	1276,6704
DVKETTPASLMPNMWPYHGQENKKFAGAVP	30	1,51E+04	3342,6165
LDKAAANYLALPE	13	1,50E+04	1387,7346
M(+15,99)PLLALPAHLEAP	13	1,50E+04	1387,7534
KDLPSPVAK	9	1,49E+04	953,5545
QNEEWFFPGLGANV	14	1,49E+04	1606,7415
QNEEWFFPGLGRQ	13	1,49E+04	1606,7527
QNEEWFFPGLLDL	13	1,49E+04	1606,7666
LEGATGTDNLGEGKG	15	1,49E+04	1417,6685
LEFGLSMVN(+,98)TVAH	13	1,49E+04	1417,6912
DVLATPVGAKALAP	14	1,48E+04	1321,7605
SVLALLPETALVP	13	1,48E+04	1321,7856
EKGVLLVGPN	10	1,47E+04	1024,5917
GGKKRLVGPN	10	1,47E+04	1024,6141
VFVLPDLVN	9	1,46E+04	1014,575
VFVPEVVNL	9	1,46E+04	1014,575
VFVPVEVGGL	10	1,46E+04	1014,575
VFVPTLGVSP	10	1,46E+04	1014,575
VFVPEVVQV	9	1,46E+04	1014,575
VFVPEVTPK	9	1,46E+04	1014,575
VFVLTKTAH	9	1,46E+04	1014,5862
VFVPNGGVKV	10	1,46E+04	1014,5862
DVKESDAGPSATLL	14	1,45E+04	1401,6987
LTAGESGLGGEGELL	15	1,45E+04	1401,6987
NLNLLPVALRLS	12	1,45E+04	1321,8081
YSNSPKLLYLVQ	12	1,45E+04	1423,771
SSALYLLKPFGTQ	13	1,45E+04	1423,771
LLM(+15,99)VSPGADGGK	12	1,45E+04	1159,5906
AHSLRYVTR	9	1,44E+04	1101,6042
HLSYEREELNEEEAKGVH	18	1,43E+04	2168,0132
HQGGNM(+15,99)GEGRDTPATETT	18	1,43E+04	1873,7861
SVEHN(+,98)LN(+,98)LVAPEGVS	15	1,43E+04	1565,7573

VSEHN(+,98)LDLVAPERS	14	1,43E+04	1565,7686
GDDGGPDNGGME	12	1,42E+04	1119,3774
GDGPGDGNDGME	12	1,42E+04	1119,3774
GDDYDSNGMF	10	1,42E+04	1119,3816
PQNFVVVK	8	1,41E+04	929,5334
AWLTPQSADKPV	12	1,41E+04	1311,6824
EKLPTQSGEKPV	12	1,41E+04	1311,7034
GENDYRGLLV	10	1,40E+04	1134,5669
LVVVPGTPPDL	11	1,40E+04	1105,6382
HNLNNPMGSELFNPR	15	1,39E+04	1738,8208
GQEFRQDGHHQKVVG	15	1,37E+04	1720,8394
LGGVLPHLNAP	11	1,35E+04	1086,6187
LAALAPHLNAP	11	1,35E+04	1086,6187
GSDAALGDGLL	11	1,35E+04	987,4872
GSDAARGELL	10	1,35E+04	987,4985
PNPQ(+,98)LLESGVVHE	13	1,34E+04	1418,7041
PEDQ(+,98)FRQQ	8	1,34E+04	1047,4622
QSADKPVRAY	10	1,34E+04	1133,5828
VTVDGKLLLSVPM(+15,99)	13	1,34E+04	1386,7793
SLASGFTLQPLKDLSGELWMGLAS	24	1,32E+04	2520,2932
QAWLTPQSDAKPV	13	1,31E+04	1439,7407
LTGAAGELKTLHTE	14	1,31E+04	1439,762
TKGVLHGVGPP	11	1,31E+04	1060,6028
QTGVLFLGRA	10	1,31E+04	1060,6028
QPQQGESLR	9	1,30E+04	1041,5203
QPQQWSLR	8	1,30E+04	1041,5356
LWETYFDEGVDRPETY	16	1,29E+04	2018,8896
PGHSRNQDSSGV	12	1,28E+04	1239,5591
PGGPSPVGGADGVSS	15	1,28E+04	1239,573
PGHGPPPHDSSR	12	1,28E+04	1239,5745
QVDVFVPEVGRLQ	13	1,24E+04	1484,7986
DVLLAHN(+,98)PGLLLPA	14	1,24E+04	1442,8132
ADLLLGRGRSSVSL	14	1,24E+04	1442,8206
LSFPTPLFPG	10	1,22E+04	1074,575
RSQEQ(+,98)EVGEANDGK	14	1,22E+04	1546,6858
SADVFLPQ(+,98)VNVVS	13	1,22E+04	1374,7031
LGEPSHDTDGPGV	13	1,21E+04	1279,5681
EAVPSHDAMPVQ	12	1,21E+04	1279,5867
RFKDHEFSTGPGGHHQGAVR	20	1,21E+04	2219,0732
LADSLGVGLVM(+15,99)VPEVAVR	18	1,21E+04	1840,0127
LGLLPSYSNSPQLF	14	1,19E+04	1534,803
VFVPQ(+,98)VAAALW	11	1,18E+04	1200,6543
MPGFVLLVSAAP	12	1,18E+04	1200,6577
VFVPEVGRLW	10	1,18E+04	1200,6655
GPGKEASGHHGGHHKQVR	18	1,18E+04	1874,936
GPGQELAAHGGPHHQKVGV	19	1,18E+04	1874,95
YVVAVVKTAVFM	12	1,18E+04	1325,7417
VAFPPTGPVVVLM	13	1,18E+04	1325,7417
GLEETYWDENSSSDGPLHTPAETF	24	1,18E+04	2681,1404

AVQ(+,98)ETYGM(+15,99)VANGTC(+57,02)NGPLHVVAETM(+15,99)	25	1,18E+04	2681,177
GVDLPSNPSWDANDH	15	1,17E+04	1622,696
VTVGADGNVPGA	12	1,17E+04	1055,5247
MLKGKKFAPG	10	1,17E+04	1075,6211
LHPPC(+57,02)VKLL	9	1,17E+04	1075,6211
MLKGKKAFGP	10	1,17E+04	1075,6211
LSATGKVVSSK	11	1,17E+04	1075,6238
GKMKGMAKLL	10	1,17E+04	1075,6245
MLKGKVKC(+57,02)L	9	1,17E+04	1075,6245
GKKGVLHGAPL	11	1,17E+04	1075,6501
EAGLMAANNALWNL	14	1,16E+04	1486,7239
WALMAAPDSLWNL	13	1,16E+04	1486,7278
GSFGDKMGGGLSSQ	14	1,16E+04	1326,5874
NKRSPGGRYN	10	1,15E+04	1147,5847
NKRSC(+57,02)SRVAA	10	1,15E+04	1147,5879
HSSGAAQGSLNEDVGQTSY	19	1,15E+04	1906,8293
EVFVPRAGM(+15,99)PV	11	1,14E+04	1216,6274
DVFLPEVTNLA	11	1,14E+04	1216,634
YYKVVLDVK	9	1,13E+04	1125,6433
LEFGLTESDTRLH	13	1,13E+04	1516,7522
LEFGLPGYDTRLH	13	1,13E+04	1516,7673
GKDNQKEGSLR	11	1,11E+04	1230,6316
MWPYHGKENQKWAATL	16	1,11E+04	1958,946
TAVFPPEKAFVLM	13	1,11E+04	1448,7737
ELLDMYVHLAHE	12	1,10E+04	1468,7019
VGDLPGC(+57,02)TQDEEASEPAETF	20	1,08E+04	2150,8948
HLTGHYPKSW	10	1,08E+04	1224,604
RAAGVNLTR	9	1,07E+04	956,5515
GNPEN(+,98)EFQKF	10	1,06E+04	1209,5303
GGGPQ(+,98)DEFQKF	11	1,06E+04	1209,5303
GKQ(+,98)NQKDASLR	11	1,06E+04	1244,6472
EGLAASALLM(+15,99)ADPY	14	1,05E+04	1436,6858
MLGGVSKN(+,98)ANAR	12	1,03E+04	1217,6187
PDLLPHWNLN	10	1,03E+04	1217,6194
AFNLN(+,98)EQLARKL	12	1,03E+04	1416,7725
AFNLDEKLARQL	12	1,03E+04	1416,7725
GEGN(+,98)N(+,98)HAGSLGE	12	1,03E+04	1142,4475
LATANYAAALALEP	14	1,02E+04	1387,7346
GSDAGKGNGLLMWPYHGKDNKKWGTVV	27	1,01E+04	2914,4548
SNLNAEVSVSF	11	1,01E+04	1165,5615
ADVFVPEVQVV	11	1,01E+04	1200,6389
ADVFVPSVTGPL	12	1,01E+04	1200,6389
LYNNGRTPLVL	11	9,97E+03	1258,7034
LWGMDALNMMGANN	14	9,91E+03	1536,6523
ELLM(+15,99)WPYHGK	10	9,89E+03	1288,6274
ELLFEGPGWGGK	12	9,89E+03	1288,645
SVQKENGGAGEKHKRGPS	18	9,83E+03	1864,9502
DVKESDAAEVGG	12	9,82E+03	1175,5305
DVKESDAARGE	11	9,82E+03	1175,5417

DVKESN(+,98)AAERG	11	9,82E+03	1175,5417
PFLALLEDWVW	11	9,69E+03	1387,7175
KLDMYPSLLE	10	9,62E+03	1207,6157
KLDFFPANKE	10	9,62E+03	1207,6238
MLVLGAMESASGK	13	9,56E+03	1292,6467
VFPPVSM(+15,99)SAVLM	12	9,56E+03	1292,6509
EPGNAPANGFGYDDL	15	9,52E+03	1535,6528
FVN(+,98)YGPEMFGDFL	13	9,52E+03	1535,6643
MDDYGFQ(+,98)LLGVMF	13	9,52E+03	1535,6677
FVDYGM(+15,99)ELPANHQ	13	9,52E+03	1535,6714
QGPAPDLEFGYDDL	14	9,52E+03	1535,678
MDDYGFQ(+,98)LVATLY	13	9,52E+03	1535,6853
MATPSC(+57,02)EC(+57,02)	8	9,40E+03	954,3245
TQGVLRALAP	10	9,37E+03	1024,6029
YPM(+15,99)GEDLM(+15,99)	8	9,33E+03	986,3725
YPETMSME	8	9,33E+03	986,3725
LGEETLLFPGQDSGDSGMLLPAETM(+15,99)	25	9,29E+03	2623,2031
NLLAPLVGPGG	11	9,25E+03	1006,5811
TQGVLAPKPP	10	9,25E+03	1006,5811
STAGGGPAVSGTRPY	15	9,23E+03	1376,6685
HEQ(+,98)LKNTLEQH	11	9,23E+03	1376,6685
LWPSATNPTAGGPQL	15	9,20E+03	1508,7622
FLQVPNLPWFEEF	13	9,11E+03	1664,8237
FLKPVNLPGEFEEF	14	9,11E+03	1664,845
EQLPM(+15,99)AQADALWVQ	14	9,08E+03	1614,7712
WAQLPTGN(+,98)MALWNL	14	9,08E+03	1614,7864
LVDENGNSVFN(+,98)GSVR	15	8,95E+03	1606,7588
LVDQ(+,98)NGNSVFDGSVR	15	8,95E+03	1606,7588
MWPSGGVGKENQKVATGDV	19	8,84E+03	1958,9519
YVMNSVALADNL	12	8,84E+03	1308,6384
PRVPSSEVLW	10	8,78E+03	1168,624
MPGRTPLVLW	10	8,78E+03	1168,6426
SVVAANLAAALPE	13	8,78E+03	1224,6714
NPLALLVGPN	10	8,69E+03	1006,5811
VRPRLVGPN	9	8,69E+03	1006,6036
NHVMSTFLA	9	8,68E+03	1018,4906
AMFFKFTGA	9	8,68E+03	1018,4946
VVDQ(+,98)NGNSVFDGSVR	15	8,57E+03	1592,7429
DALLALRPNA	10	8,56E+03	1052,5979
LVNDRGDSVFN(+,98)GQ	13	8,46E+03	1420,6582
NQDSSRNLFSGL	12	8,44E+03	1336,6372
VLSGHASGYVFNGQ	14	8,32E+03	1434,6892
FLGGEGGPM(+15,99)LL	11	8,28E+03	1105,5479
FLGDAGPGM(+15,99)LL	11	8,28E+03	1105,5479
GNAGEDEFQFK	11	8,27E+03	1240,5359
NGQQ(+,98)N(+,98)KVGFTM(+15,99)	11	8,27E+03	1240,5393
LTDGNEEREVKEEEAQRHL	19	8,23E+03	2281,0933
LALLLHTVGSL	11	8,17E+03	1135,6965
LALLPAVSGAPK	12	8,17E+03	1135,6965

GDDSLVSNLQ	10	8,10E+03	1046,488
GDDSLADNLK	10	8,10E+03	1046,488
LMLAPGKLAL	10	8,06E+03	1025,6306
DKSNHGPEGFYDDL	14	8,04E+03	1592,6743
MDDYVLQFMHSHA	13	8,04E+03	1592,6753
MDPVHVGEFGYDDL	14	8,04E+03	1592,6816
TPNEDGLADVGYDDL	15	8,04E+03	1592,6841
VVDENGNSVFGNDDL	15	8,04E+03	1592,6953
TPNEDGNSVFTADNL	15	8,04E+03	1592,6953
QVEAPGPGEFGYDDL	15	8,04E+03	1592,6995
QSHNGVLEFGYDDL	14	8,04E+03	1592,7107
VFDYGM(+15,99)KGFDATLN	14	8,04E+03	1592,718
DLWDPPAGGGPE	12	8,02E+03	1209,5303
DVEGGMLVYPM	11	8,02E+03	1209,541
DVFVPEVGRLR	11	7,98E+03	1285,7141
TLPQNFLVKGVL	12	7,98E+03	1327,7864
TLPQNFLVLKR	11	7,98E+03	1327,7976
APSLAGRLVGPN	12	7,93E+03	1150,6458
M(+15,99)M(+15,99)AGLPPPC(+57,02)	9	7,83E+03	1004,413
YEEMSVFT	8	7,83E+03	1004,4161
LAGRLVPQ(+,98)VLAN	12	7,75E+03	1250,7346
KVDYAR	6	7,74E+03	750,4024
DARAADLLMWPYHGKENQKWKDL	23	7,74E+03	2784,3804
LSEDLNRL	8	7,72E+03	958,5084
LGSAVLLVGATP	12	7,67E+03	1096,6492
VTKVPEVGRL	10	7,67E+03	1096,6604
DVFVPEVLTPK	11	7,63E+03	1242,686
AVGVLPHDAPLN	12	7,60E+03	1201,6455
LGGVLPHDANLP	12	7,60E+03	1201,6455
AVGVLPHDANLP	12	7,60E+03	1201,6455
KADSHNLPLLLDK	13	7,56E+03	1462,8142
LSNSHNLPLLRSL	13	7,56E+03	1462,8257
NLPLLGNLL	9	7,52E+03	965,5909
NLPLLGGAVL	10	7,52E+03	965,5909
LEKRLVGPN(+,98)GL	11	7,50E+03	1195,6924
ELKRLVGPN(+,98)GL	11	7,50E+03	1195,6924
LYNDGTRPLVL	11	7,48E+03	1259,6873
DNQKWSLR	8	7,44E+03	1045,5305
KTELTMR	7	7,44E+03	877,4691
KSARTHQYPGTVGPAPQGTQ	20	7,43E+03	2080,0449
QSSLRHVFTSEKHEKNPG	18	7,43E+03	2080,0449
VSGPKVGVLGNP	12	7,42E+03	1122,6396
NQLDNYALFLASATL	15	7,37E+03	1652,8411
NKLDNHKGGDDKVTL	15	7,37E+03	1652,8481
FMNEFN(+,98)PVADKELMM(+15,99)	15	7,30E+03	1831,783
ENFLSMMPVDFENLF	15	7,30E+03	1831,8162
TLLTPGHVNPLLPA	14	7,28E+03	1441,8293
PAAEQHQQLR	10	7,28E+03	1176,5999
LVVVGPVPVNN	11	7,24E+03	1105,6494

VVLDVKESDAARGELL	16	7,23E+03	1712,9309
LLPAYSNSPKLLY	13	7,22E+03	1477,8181
LLPAYSNSKPLLY	13	7,22E+03	1477,8181
YSNSPKLLY	9	7,20E+03	1083,5601
QLGEPSWPVDGSH	13	7,16E+03	1407,6418
QLGEPSSYSVDVQ	13	7,16E+03	1407,6519
KDARLKM(+15,99)GVGK	11	7,06E+03	1217,6914
KWRLQFGVGK	10	7,06E+03	1217,7034
LDVKQ(+,98)SDAARADLL	14	7,02E+03	1514,7939
PLQPATQML	9	6,97E+03	997,5267
AAGPKRRDAG	10	6,97E+03	997,5417
LFTGPAYGLGNDQL	14	6,95E+03	1464,7249
AVVGM(+15,99)FPVAVK	11	6,90E+03	1132,6313
NHVKGVLVGPN	11	6,90E+03	1132,6353
GPVTRSRTFA	10	6,83E+03	1090,5884
EEARRLKFN	9	6,81E+03	1161,6255
VKGSNDFGVVPTK	13	6,77E+03	1346,7195
LRSFWNLN	8	6,74E+03	1048,5454
RLPHWNLN	8	6,74E+03	1048,5566
LGVPHVSNLN	10	6,74E+03	1048,5664
DVKGAMSPGVSGLLMWPYHGAGDNKKFVPAQ	31	6,69E+03	3256,616
GAGKLKVGSE	10	6,69E+03	944,5291
ESPANLVSLMMWPYHGKENQKTGLASL	27	6,68E+03	3000,4836
NLPLLEGVL	9	6,62E+03	966,575
NLPLLERL	8	6,62E+03	966,5862
WSLQ(+,98)AN(+,98)GTGVL	11	6,61E+03	1146,5557
YLDLQPC(+57,02)PM(+15,99)	9	6,55E+03	1151,499
DQNHRDFFL	9	6,54E+03	1190,5469
YNHAAGM(+15,99)E	8	6,53E+03	907,3494
LDGVGGVYRA	10	6,51E+03	1005,5243
LDQNHRDFFLA	11	6,49E+03	1374,668
KKSAEHQQC(+57,02)NGLLGNTVPPN	20	6,48E+03	2191,0803
SAAVGLLLQPP	11	6,47E+03	1064,623
MSPGNREHA	9	6,46E+03	997,4399
M(+15,99)APEVVEAH	9	6,46E+03	997,4539
C(+57,02)AVVAANALPVVAN	14	6,43E+03	1367,7231
GSSVVTEPLPVVAN	14	6,43E+03	1367,7295
GDN(+,98)SLWNLRTLL	12	6,36E+03	1401,7251
ADMGLWNLVGTLL	13	6,36E+03	1401,7327
FLGPYHGQDNQKWKNN	16	6,32E+03	1944,9231
SQGAVTHDLVAEP	13	6,32E+03	1322,6467
GKSASAVHGGGVAPE	15	6,32E+03	1322,6577
GPAEANPPLLALPEGVAH	18	6,31E+03	1751,9207
PEREPLALLVHQDVH	15	6,31E+03	1751,9319
NSVTLGVDLSSDAETM(+15,99)	16	6,25E+03	1653,7402
NSTVLGVN(+,98)LC(+57,02)EAETF	15	6,25E+03	1653,7556
VDPAQVLPNP	10	6,22E+03	1048,5552
YVGHKGLLY	9	6,22E+03	1048,5706
GRSVMVDPLLTVMPN	15	6,18E+03	1627,8425

TKMVAVDPLLSNLVE	15	6,18E+03	1627,8855
RLESPGGR	8	6,12E+03	870,4671
RANTDLAPSK	10	6,10E+03	1071,5671
RANTN(+,98)RLAR	9	6,10E+03	1071,5896
SAVFPDLGGTL	11	6,09E+03	1075,5549
SAVFN(+,98)PLGGTL	11	6,09E+03	1075,5549
LKRLVGPN	8	6,06E+03	895,5603
PEDQ(+,98)FQKF	8	6,05E+03	1038,4658
PEDEFQKF	8	6,05E+03	1038,4658
NKLMAGGSHHGTL	13	6,01E+03	1321,656
QNLDPAAQPAANL	13	6,01E+03	1321,6626
MVTVLGKVPDE	11	5,96E+03	1186,6267
AVTPGM(+15,99)PSVTVL	12	5,96E+03	1186,6267
LLGGDFDGGAP	11	5,95E+03	1017,4767
LGELGGDMGGL	11	5,95E+03	1017,48
DAARADLLMWPYHGKDNAGKWGTVV	25	5,95E+03	2770,3647
KLSGLAPAGPN	11	5,90E+03	1023,5712
PGHPFLQDSNGTPTFSGL	18	5,89E+03	1870,885
PGHSRNGADSVVSNAFSGL	19	5,89E+03	1870,8921
YYKVVEVVQEDVLAKSANN	19	5,87E+03	2167,116
LPVDVFVPERGVL	13	5,83E+03	1438,8184
DALALLNPV	9	5,83E+03	924,528
SLPGLHKSHL	10	5,82E+03	1087,6138
MVSPYHGQDNKKVGDSAL	18	5,80E+03	1944,9363
VSELKGLVVGPN	12	5,75E+03	1210,6921
VSELKGVLVGPN	12	5,75E+03	1210,6921
DVQESN(+,98)PKASTLL	13	5,75E+03	1401,6987
LTAGESN(+,98)AALQDVL	14	5,75E+03	1401,6987
DVQESSVALDNLL	13	5,75E+03	1401,6987
LEKGVLVGPNGL	12	5,74E+03	1194,6973
LEKRLVGPNGL	11	5,74E+03	1194,7085
HGKENQKVSSVGL	13	5,71E+03	1381,7314
PEEVAVH	7	5,69E+03	779,3813
FDFPLTMP	8	5,59E+03	966,4521
LHVTRPVDRQ	10	5,57E+03	1219,6785
LLN(+,98)TSNSNNGALNESSGC(+57,02)PTNLL	23	5,55E+03	2390,1018
LVSYLGWPLVSPP	13	5,53E+03	1426,7859
NVNEQLARKL	10	5,50E+03	1183,6672
WRGLALC(+57,02)GTKPVW	13	5,49E+03	1542,813
LVGVRTLEPH	10	5,49E+03	1119,6399
NNAPYLGGGGLQ	12	5,43E+03	1159,5623
EAVPVAPDLSY	11	5,43E+03	1159,5762
LNAGPSDSLAKP	12	5,41E+03	1168,6086
SSTSYGVK	8	5,36E+03	827,4025
SSTSYGVK	8	5,36E+03	827,4025
EVATESSTVPNSAGPQ	16	5,34E+03	1572,7268
WPLPSAMTDAAPGLM(+15,99)	15	5,34E+03	1572,7317
DERWYVK	7	5,33E+03	994,4872
SKAVPELAQL	10	5,32E+03	1054,6023

QLVGEARLQ	9	5,32E+03	1012,5665
NGVLALPLP	9	5,30E+03	892,5381
VTTN(+,98)GKKGSM(+15,99)VKLL	14	5,29E+03	1491,833
LEPLC(+57,02)GQPL	9	5,24E+03	1025,5215
AALLERDNKYVKA	13	5,23E+03	1489,8252
VVDEDGNSVYVSKAAGPAGDVV	22	5,23E+03	2147,0381
NSNNQLDKAPGGEARM(+15,99)GNSL	20	5,21E+03	2087,9653
DAPSAYDVQ	9	5,20E+03	964,4138
REEAREEEAAGR	12	5,20E+03	1401,6597
TTVSGGEEATPSVAP	15	5,20E+03	1401,6624
NSGLGHVFANSLE	13	5,18E+03	1343,647
TASDTSAQA	9	5,15E+03	850,3668
NGATTSETA	9	5,15E+03	850,3668
TAEALM(+15,99)EA	8	5,15E+03	850,3742
SSELAGRLVGPN	12	5,07E+03	1198,6306
NNKVEAMAADGAVLLMWPY	19	5,05E+03	2092,012
AASGVLTTKGQT	12	5,04E+03	1132,6089
FFLKPGNVAR	10	5,00E+03	1147,6501
FFLKPGNLGR	10	5,00E+03	1147,6501
LPVLC(+57,02)AVPP	9	5,00E+03	964,5416
SLWGFTLTAEVPSVAGGALTFGSRP	25	4,94E+03	2520,301
SLTVYETLTDPPKVEALTFGSRP	23	4,94E+03	2520,311
KVDYVK	6	4,94E+03	750,4276
KDVYVK	6	4,94E+03	750,4276
LFVPEVGQTL	10	4,93E+03	1101,6069
LLEGM(+15,99)SSGGGLAP	13	4,92E+03	1203,5806
AALALPHGGAP	11	4,91E+03	973,5345
AAALLPHNAP	10	4,91E+03	973,5345
AVGVLPHGGPA	11	4,91E+03	973,5345
SDLEVVFDAKKTKPA	15	4,89E+03	1646,8879
APTDLMDLLM(+15,99)DAPY	14	4,89E+03	1580,7102
TSSQHLQ	7	4,86E+03	799,3824
LTC(+57,02)LGESSVPAR	12	4,84E+03	1288,6445
MLGWGSLLGEVQ	12	4,84E+03	1288,6484
TTHSTYVGQGNPGSHR	16	4,83E+03	1697,7869
RGDPGLAGAGPTSYSHR	17	4,83E+03	1697,8232
FHSTVALFPGMGM(+15,99)TF	15	4,82E+03	1657,7632
YVVVSALKM	9	4,80E+03	1008,5677
FFLTEHTF	8	4,80E+03	1040,4967
FFLTHETF	8	4,80E+03	1040,4967
FVPEVVNL	8	4,77E+03	915,5065
M(+15,99)PAVYHGKENQKRSGEL	17	4,76E+03	1958,9631
TTLLLGN(+,98)PSPAPK	13	4,73E+03	1308,7288
TTLLLGDPALAPQ	13	4,73E+03	1308,7288
AHAKLKGY	8	4,72E+03	886,5024
WQYLADSSLPAY	12	4,70E+03	1412,6611
FVFPRYPF	8	4,69E+03	1071,5542
SVLLLGNPELLHAAVP	16	4,67E+03	1641,9453
SVLLLGNPQ(+,98)LLHPGLA	16	4,67E+03	1641,9453

VPFLVLK	7	4,64E+03	814,5316
VPFLVKL	7	4,64E+03	814,5316
LM(+15,99)GDGSYSGLVSGLEWATTN	20	4,61E+03	2072,936
YGNAWELGSVLGSYSGGAATN	21	4,61E+03	2072,9438
DDNLMHTVAVSGLEWATTN	19	4,61E+03	2072,9473
KPVVAGY	7	4,61E+03	732,417
LVQNGSGNSVFDGQ	14	4,58E+03	1420,6582
LGPTGGMTLGEPY	13	4,57E+03	1291,6118
VYVVVGPVVPFVHG	14	4,56E+03	1466,8286
RGGATGPPPDGPV	13	4,54E+03	1176,5886
LVVAFC(+57,02)GGGAGGL	13	4,54E+03	1176,5962
RALEPDNRLQ	10	4,52E+03	1210,6418
LM(+15,99)PATYPYPSSSMSPETY	18	4,49E+03	2036,8748
M(+15,99)VAGRVFVVLM(+15,99)	11	4,49E+03	1252,6672
M(+15,99)SPAPVLSFSADQ	13	4,48E+03	1364,6282
NLSALTTKGPN	11	4,45E+03	1114,5981
TFQKLGV	7	4,44E+03	791,4541
VKTLPETNAVL	11	4,44E+03	1183,6812
VTGALGRSKAPK	12	4,44E+03	1183,7036
SPDGVVSTYYVNFQ	14	4,42E+03	1574,7253
VQ(+,98)GAVQLLKASAPQV	15	4,42E+03	1508,8562
VQ(+,98)GGLKVVGVTGAPQV	16	4,42E+03	1508,8562
EGYVNMSVLAWNL	13	4,38E+03	1494,7178
GDGSLRM(+15,99)L	8	4,37E+03	863,4171
SVLPLGVATHAGAPV	15	4,35E+03	1387,7822
TLAGAHARTLPLPA	14	4,35E+03	1387,7935
SSNEAM(+15,99)C(+57,02)ERGSNGT	14	4,33E+03	1514,5725
FLTSPPLFPGSVSL	14	4,33E+03	1460,7915
MNVSLNLGGGGQ	12	4,33E+03	1145,5498
MAAADALAWVQ	11	4,33E+03	1145,554
HHKQVR	6	4,30E+03	803,4514
PMVVVPEDVSLTMDAGAPL	19	4,26E+03	1939,9634
VDLVVPQ(+,98)N(+,98)VSLVMTSGPSP	19	4,26E+03	1939,9812
LM(+15,99)YVGPC(+57,02)PETY	11	4,25E+03	1344,573
LFPGQM(+15,99)M(+15,99)PETY	11	4,25E+03	1344,573
LLLGYLETPALLGGNS	16	4,24E+03	1629,8977
ENQKWSLGV	9	4,24E+03	1059,5349
KFGAGRLVGNP	11	4,22E+03	1114,6248
LGVRPGGFFNL	11	4,22E+03	1175,645
LGVRNPFFGGL	11	4,22E+03	1175,645
GDDSLWNLVGW	11	4,21E+03	1260,5774
MVKNNGRTPVLL	12	4,19E+03	1340,7598
LYVPNGRTPLVL	12	4,19E+03	1340,7815
KVVDNFFTETSAPR	14	4,17E+03	1609,8101
KVVDNFFGGDFLGPV	15	4,17E+03	1609,814
GYDN(+,98)N(+,98)GLHQVG	11	4,17E+03	1174,489
FMPGVPGPLKFH	12	4,17E+03	1325,6953
WSNVTLTQQT	10	4,15E+03	1176,5774
YVNGPKLLYVVQ	12	4,15E+03	1391,7812

M(+15,99)TAFLGSLAP	10	4,14E+03	1022,5107
VPERGVDVE	9	4,07E+03	998,5032
VPEVNVM(+15,99)PV	9	4,07E+03	998,5107
LSEEAVVPLKFN	12	4,07E+03	1344,7288
ATARPPRSNGAATA	14	4,04E+03	1339,6956
YPEASYPY	8	3,97E+03	988,4178
YVVPGPLHKFVGP	13	3,96E+03	1408,7866
PM(+15,99)QGGKRLVGPN	12	3,95E+03	1268,666
AKPFAQLFPGMWPY	14	3,90E+03	1651,822
PGQEVFGANHPHQKVR	16	3,87E+03	1799,918
NSNNTTPGKNHGPGHSDKA	19	3,87E+03	1931,8833
NSNLELDQLWSWPM(+15,99)LA	16	3,87E+03	1931,9087
GTTN(+,98)C(+57,02)SGM(+15,99)L	9	3,85E+03	956,358
LGLLPSYSNAC(+57,02)TMF	14	3,84E+03	1572,7317
LYNNGRTLPVLVQ	13	3,83E+03	1485,8303
VTTN(+,98)GQKGSFVK	12	3,79E+03	1265,6616
NQLDNYGKFTAGRGL	15	3,77E+03	1652,8269
VFVPLDGRLL	10	3,75E+03	1127,6702
NEKSLLF	7	3,75E+03	849,4596
NSGVTLLF	8	3,75E+03	849,4596
QEQEEQEETVAQNVGK	16	3,74E+03	1844,8389
LTPGSSKLTY	10	3,74E+03	1065,5706
NSDGVVSVM(+15,99)DGGA	13	3,73E+03	1222,5137
DVKESAVGAN(+,98)GELL	14	3,71E+03	1401,6987
DVKEGNGVTNGSLL	14	3,71E+03	1401,71
MLTKLAADSEQVP	13	3,71E+03	1401,7173
DSPPTVSLM(+15,99)GMTAGH	15	3,69E+03	1515,6697
LNPMLRYL	8	3,68E+03	1018,5634
WYEHVPPPNF	10	3,66E+03	1284,5928
VFQPFKDPNK	10	3,66E+03	1218,6396
KGSVFKLL	8	3,66E+03	890,5589
NSNLGELSVDMC(+57,02)	12	3,65E+03	1337,5591
SAGPEVHHVM(+15,99)DGS	13	3,65E+03	1337,5669
ELPSPSDMGFTAS	13	3,65E+03	1337,5808
DVPPPVQAPLTLL	13	3,64E+03	1358,781
EARRLFK	7	3,63E+03	918,5399
Q(+,98)ARRLKF	7	3,63E+03	918,5399
GDDSLWGGLRWYVK	14	3,62E+03	1650,8154
QAWLPTQAGAGDPGLPRP	18	3,62E+03	1830,9375
KAWLPTESWQPVHGPV	16	3,62E+03	1830,9417
LQQGGVVLQ	9	3,59E+03	940,5342
PGAM(+15,99)PLVLK	9	3,59E+03	940,5416
C(+57,02)AVVLPEEVVAN	12	3,59E+03	1298,6541
LSNDQLDQNHYSPAFL	16	3,58E+03	1860,8643
HLVGPDKRKLLS	12	3,58E+03	1361,8142
FLQPVNLVSPM(+15,99)EEFVAS	17	3,57E+03	1921,9495
FAAFGGNYLVALFYLVAS	18	3,57E+03	1921,9978
SHNLPLLR	8	3,57E+03	948,5505
GDDSPPTETGE	11	3,56E+03	1103,4255

LLLVM(+15,99)PGHGSLS	12	3,56E+03	1238,6692
VQNNPSDSDLFNPR	14	3,54E+03	1601,7434
NLNNPSN(+,98)SDLFNPR	14	3,54E+03	1601,7434
MVKWRVNGDFVTNGSRYM	18	3,54E+03	2159,0403
LFNATDNALEP	11	3,53E+03	1203,5771
METGAGVLAMQP	12	3,53E+03	1203,5627
DGDSATVNLAKN	12	3,53E+03	1203,573
HHLVPSNLGL	10	3,52E+03	1085,5981
LRPHGENLN	9	3,52E+03	1048,5413
LAAAGGATGPL	11	3,51E+03	897,4919
LGPGFGLPSM(+15,99)TTGLH	15	3,50E+03	1499,7441
MGEGLKPGGSAVGQL	15	3,49E+03	1399,7129
VLDDLVESVFDGQVQEGQLF	20	3,48E+03	2236,0898
LLN(+,98)TSNSNNKLNSSGPNNSNDTSPK	25	3,45E+03	2617,2214
GSDAVTAAALLM(+15,99)ADPYHGK	19	3,45E+03	1902,9146
VSLLLGDPELHAALPV	16	3,43E+03	1642,9294
TAVN(+,98)VAKTMLGSSVLL	16	3,43E+03	1603,8855
TAVN(+,98)VAPPRGPLWLL	15	3,43E+03	1603,9084
VSLPTESWAGPV	12	3,43E+03	1241,6292
GYRPTLLNPR	10	3,42E+03	1185,6619
LGGNVM(+15,99)VVQPLVD	13	3,40E+03	1355,7119
AMHKNHVGDFFLA	13	3,40E+03	1485,7188
VAPDAM(+15,99)APK	9	3,38E+03	914,4531
GLPDATVGSV	10	3,38E+03	914,4709
LKVPNLPGEFEEF	13	3,38E+03	1517,7766
DTM(+15,99)SDVQ	7	3,36E+03	810,3066
SSAN(+,98)TSM(+15,99)P	8	3,36E+03	810,3066
VDYN(+,98)PVGYYVALK	13	3,35E+03	1500,75
LNNPSDSRASSVEGGVGDLL	20	3,34E+03	1985,9653
LNNPSDSDLFNRANPSLL	18	3,34E+03	1985,9807
TKVVPEVGGVL	11	3,31E+03	1096,6492
KSASVVHQDSSEQHQKLGV	19	3,31E+03	2063,0396
LVPVEPSTGQVL	12	3,30E+03	1237,6917
PFEC(+57,02)AVGDE	9	3,28E+03	1022,4015
NWYNDQPVLSVN	12	3,27E+03	1447,6731
FFSVSPAVGLVGTAC(+57,02)KH	17	3,27E+03	1775,9028
PNTPASPAVGLVGFSSLY	18	3,27E+03	1775,9094
EGLLM(+15,99)DAPYHGK	12	3,25E+03	1345,6335
EGLLM(+15,99)GEPYHGK	12	3,25E+03	1345,6335
PGVLQKVGGGP	11	3,23E+03	1007,5764
TPTETYSTVSM(+15,99)SSGNAM(+15,99)PKV	20	3,22E+03	2118,9448
DAAKQLAQ	8	3,22E+03	843,445
MLDAKVAP	8	3,22E+03	843,4524
EVAPGYLGGSE	11	3,20E+03	1077,4978
HGGDGEGMSGLVSGLEWATTN	21	3,20E+03	2073,906
DGNFGSYSGLVSGLEWATTN	20	3,20E+03	2073,928
AWLPTESADKVP	12	3,15E+03	1312,6663
NLGSNASAAAL	11	3,15E+03	987,4985
LASPPVLVGPN	11	3,13E+03	1062,6074

VSHWPAPTPAEP	12	3,13E+03	1287,6248
EGNDYRGLLVGE	12	3,12E+03	1320,6309
PLEVVN	6	3,12E+03	669,3697
NKENLRLKFN	10	3,12E+03	1274,7095
LEDAKRLKFG	10	3,12E+03	1175,6663
LTAFVGPTVEAP	12	3,11E+03	1200,6389
MLVVVGPTPPFPR	13	3,09E+03	1408,79
KLSDGPYGSNQGPLA	15	3,07E+03	1502,7366
LADPSASALVM(+15,99)	11	3,06E+03	1089,5376
LADPSSAALVM(+15,99)	11	3,06E+03	1089,5376
VM(+15,99)AM(+15,99)VRHVFDGQ	12	3,06E+03	1420,6592
TFKRLVGPN	9	3,06E+03	1030,5923
QNEQ(+,98)WFFPGLNV	12	3,03E+03	1478,6831
VSLALPGATP	10	3,02E+03	924,528
GKLNAAALAP	10	3,02E+03	924,5392
NLSAFLN(+,98)SDSPNPPE	15	3,02E+03	1601,7209
LSEELALAK	9	3,00E+03	972,5491
PGQEHPSAPPGPHQKTGP	18	3,00E+03	1817,8809
PGKETNHHGGHHQKVR	16	3,00E+03	1817,9146
LGDSAGPGSP	10	2,99E+03	856,3926
LGSDAGPSGP	10	2,99E+03	856,3926
SSAGDMDSNHNQ	12	2,98E+03	1261,4629
VPEVLNV	7	2,97E+03	768,4381
MLLGDVAAAN(+,98)PAP	13	2,96E+03	1239,6169
QAPDAPGAAPQSAPGHK	17	2,96E+03	1598,78
WSLQ(+,98)TDLSNL	10	2,95E+03	1176,5662
C(+57,02)FLKGVLVGPN	11	2,95E+03	1202,6482
LNLNLALPVRL	11	2,95E+03	1234,7761
NASLALNYLALPE	13	2,95E+03	1387,7346
GGGTLALYNLALPE	14	2,95E+03	1387,7346
SHNLPLLEKV	10	2,95E+03	1148,6553
SHNLPLLRSL	10	2,95E+03	1148,6665
QVRLKFGTGA	10	2,95E+03	1075,6138
KVRSGPFGTK	10	2,95E+03	1075,6138
RDELTFGSAPT	11	2,94E+03	1192,5723
ATAKVDGLFPGGL	13	2,92E+03	1244,6765
LGEGLDGM(+15,99)MD	10	2,92E+03	1052,4155
VTGADGVNPGA	11	2,91E+03	956,4563
RLTGDSVAGVM(+15,99)L	12	2,90E+03	1233,6387
SADVFLKAGPADGK	14	2,90E+03	1374,7144
LTNKYNLPMLRLY	13	2,89E+03	1637,8962
LTNKYNLPMLRYL	13	2,89E+03	1637,8962
MFVSMGVEPVHRQY	14	2,88E+03	1678,7959
MFWMLFAHPNK	11	2,88E+03	1420,6785
NALLSTNKM(+15,99)LLAP	13	2,85E+03	1400,7698
PTLLHPSHLLY	11	2,82E+03	1289,7131
ETAAEGLTC(+57,02)GVSGSP	15	2,82E+03	1434,6296
GN(+,98)PSLEGNGVL	11	2,82E+03	1056,5088
PGSRGKENQKWNNK	14	2,82E+03	1641,8335

AKTVPYVAPLLQAGLSFKH	19	2,81E+03	2039,1567
HNLNNSPDDSAAAANKT	17	2,81E+03	1738,7871
RSSKEGTGVLNP	12	2,80E+03	1243,6521
SWKTKGNSVLT	11	2,80E+03	1219,656
ETGVASNLLY	10	2,80E+03	1065,5342
YSAHSLLLY	9	2,80E+03	1065,5496
MAVVSHRVVVAN	12	2,79E+03	1280,7024
SHNLLPLQVGL	11	2,79E+03	1189,6819
LSDGGNVHEA	10	2,78E+03	997,4465
SMPEVVAEH	9	2,78E+03	997,4539
SMPEVVEHA	9	2,78E+03	997,4539
VSLLPTWLPGPAGPLPKDA	19	2,77E+03	1928,0771
WLLPTRAHVSAGVVVVGPA	19	2,77E+03	1928,0996
LGGVLPHAAPK	11	2,77E+03	1058,6235
AGTGVKHPVGTK	12	2,77E+03	1150,6458
RQ(+,98)FDPGGPPEGGGADSSRN	19	2,75E+03	1900,8298
LSPAFGPVGAVF	12	2,74E+03	1160,623
M(+15,99)VVLSVAGVSGH	12	2,73E+03	1170,6067
DQHQKLRHF	9	2,73E+03	1207,6211
QPAQ(+,98)LSSFVGPLY	13	2,72E+03	1406,708
SHWPPSHDVQ	10	2,72E+03	1188,5312
EGLPTKMAAAGPV	13	2,72E+03	1240,6484
LN(+,98)APTKTTAAGVP	13	2,72E+03	1240,6663
LADPTKSSVAGPV	13	2,72E+03	1240,6663
PSTFVDKAPQLF	12	2,72E+03	1348,7026
WFFYGN(+,98)GSLESSL	13	2,72E+03	1506,6667
WFFYGDGSLRM(+15,99)L	12	2,72E+03	1506,6965
LM(+15,99)EYGGPPEGYMPETY	16	2,71E+03	1848,7585
AQGASLFVLPL	11	2,71E+03	1114,6387
AKGALSVM(+15,99)LPL	11	2,71E+03	1114,6421
LSVPGTGYGSSDGPGQL	17	2,70E+03	1590,7524
GNPESTEATASF	12	2,68E+03	1209,5149
EDLAGAKVTAETAL	14	2,66E+03	1387,7195
MVGSLGENLTAALL	14	2,66E+03	1387,738
NDLGRLPHWH	10	2,66E+03	1243,6211
PVAVGEEEHPYPR	13	2,64E+03	1478,7153
GVPLGEEEHPYPR	13	2,64E+03	1478,7153
NGTVFN(+,98)GNVVGEQGVL	16	2,64E+03	1603,7842
NLNGPLGDADLLLM(+15,99)	14	2,62E+03	1470,7388
VSLLLGPDELPKY	13	2,62E+03	1442,802
NNASDLNFNHPPE	13	2,62E+03	1467,6379
QVLTLSGLTQLVLQ	14	2,60E+03	1511,8923
NDGVLRRGTK	10	2,60E+03	1114,6206
VHDTSNGVANLL	12	2,60E+03	1238,6255
QKLAAALNGLGGLAPL	16	2,57E+03	1505,8928
EDLLALLAM(+15,99)VVGE	13	2,57E+03	1387,7268
DATKAPLVGR	10	2,56E+03	1026,5822
RAMHSTLPGAR	11	2,56E+03	1195,6243
RSDPVPLEGAR	11	2,56E+03	1195,6309

NLDNLGVVPLPNGLM	15	2,55E+03	1564,8284
NLLVAASGVPLMGPQV	16	2,55E+03	1564,8647
LLAPVQVGPAGVL	13	2,54E+03	1232,7493
GKLLLWPF	8	2,53E+03	972,5797
GDDSVTC(+57,02)FG	9	2,53E+03	956,3546
ESVRGGPDMLFWPGLTDV	18	2,53E+03	1974,9509
TAVVQWTGNVLTVMPN	16	2,53E+03	1728,887
TSAPGVHKLVH	11	2,53E+03	1144,6353
MDN(+,98)YGFELGKPLLNENLGYPD	21	2,52E+03	2399,0991
TAVPETSVLSAL	12	2,52E+03	1186,6445
MLKVFSPVQLM	11	2,52E+03	1291,7031
TDGQKGSFVKLL	12	2,52E+03	1291,7136
ELNVSVLLVLANAM(+15,99)	14	2,50E+03	1500,8223
TAVRLPHWNLN	11	2,48E+03	1319,7097
NHARNPSDSDLFGGPR	16	2,48E+03	1738,8135
PASQSFALLM(+15,99)SGLDEVGLLAEAF	23	2,48E+03	2381,1824
GPQLDNRPLGPKA	13	2,46E+03	1361,7415
VAHTGDAHVESAL	13	2,45E+03	1305,6313
HNVHLHLHELVKVEAVRH	18	2,45E+03	2166,1921
GSLGALPLRGPN	12	2,45E+03	1150,6458
PVLPVTVPTLTPPSTVAHA	19	2,45E+03	1896,072
LTLWPSHGTGSH	12	2,44E+03	1291,6309
LTLGEPPDLGGSH	13	2,44E+03	1291,6409
PFKGGRLFGPVVL	13	2,43E+03	1385,8184
LNNPSN(+,98)SDNVGPFL	14	2,43E+03	1487,6892
GPLEVGAEEEAQR	13	2,43E+03	1383,6628
LMGATAHSGGEL	12	2,41E+03	1142,5391
FMSVMNVFFDGK	12	2,41E+03	1420,6519
MAAVDEGNLPSDVVQ	15	2,39E+03	1543,7188
KTGVLAYKK	9	2,39E+03	1006,6175
KMTPASVNGKVSMGSAERGAPVLS	24	2,36E+03	2373,2144
KAFVVGPEADVFAGVSAEAVNPLS	24	2,36E+03	2373,2214
LN(+,98)SDALNNNLM(+15,99)K	12	2,36E+03	1362,645
VGADLPGANPGA	12	2,36E+03	1037,5142
LNNPSN(+,98)SNNFNPR	13	2,36E+03	1487,6753
VNAAEGEFGHGVNF	14	2,36E+03	1446,6528
GDDSLWNLVVGSF	13	2,35E+03	1407,667
RQREETM(+15,99)	7	2,35E+03	964,4396
NSFLNDLLYLQ	11	2,35E+03	1338,6819
MMPWMM(+15,99)MM	8	2,35E+03	1103,3806
KGGAQTPLVLGV	12	2,35E+03	1138,6709
LHVGSSRLTAF	11	2,34E+03	1186,6458
RTAAEHPC(+57,02)GHQKVR	14	2,34E+03	1645,822
STGPGKEMFGGPGGHHQQVR	20	2,34E+03	2062,9756
TRPWVSVGPQ	10	2,33E+03	1125,593
VM(+15,99)PM(+15,99)QANAA	9	2,33E+03	963,4154
REFDPGHSR	9	2,33E+03	1099,5159
M(+15,99)SVAASGAPQ	10	2,33E+03	933,4225
HHAATANLAW	10	2,32E+03	1090,5308

GDDPDPHSHSG	11	2,32E+03	1119,4216
PLETSAAP	8	2,32E+03	784,3967
NAPGTAAAL	9	2,32E+03	784,4079
SSELQRLVGPN	11	2,31E+03	1198,6306
AAPEEVGELNEEEAQR	16	2,30E+03	1769,8066
AAPEEGVEVGAEEEAQR	17	2,30E+03	1769,8066
MLALYLTGPAL	11	2,30E+03	1161,6467
VKVLPHVSNKV	11	2,29E+03	1218,7449
TAVPDAMNVV	10	2,28E+03	1015,5008
TAVPDAMVVN	10	2,28E+03	1015,5008
LTLGGPAKAKNLT	13	2,28E+03	1282,7607
QAGELTLLSDAAGPV	15	2,26E+03	1440,7461
HAALPFPRPQ	10	2,25E+03	1132,614
EHEASMSLN(+,98)VGLVHM(+15,99)	15	2,25E+03	1669,7439
DVFVPLNLTM(+15,99)L	11	2,25E+03	1276,6736
PGHSRGGQM(+15,99)SGPGP	14	2,24E+03	1336,5942
PGHSRGGGAM(+15,99)SNPP	14	2,24E+03	1336,5942
EAAALLM(+15,99)WPY	10	2,23E+03	1179,5635
WLYNNGRTPLVLV	13	2,22E+03	1543,8511
FVASNPGVKFKSL	13	2,22E+03	1392,7764
LAVLPQYVNGKPLLY	15	2,21E+03	1686,9707
AERGADLLMWPYHGK	15	2,21E+03	1742,8562
LNPLVELK	8	2,21E+03	924,5644
RAPLTAALL	9	2,21E+03	924,5756
NANGKGVSVSF	11	2,21E+03	1078,5408
EDLALPGRLEM(+15,99)E	12	2,19E+03	1387,6653
LLNATPQLVL	10	2,19E+03	1080,6543
LLNPRSGLVL	10	2,19E+03	1080,6655
VVPGPKLLY	9	2,18E+03	984,6008
LLGGDGFGP	9	2,18E+03	831,4127
PC(+57,02)LVVLWFTADTPQ	14	2,17E+03	1645,8174
LDYAMPVAALD	11	2,16E+03	1177,5688
VDFVPVVFGPAF	12	2,16E+03	1292,6804
KAPFLPETAVVQ	12	2,16E+03	1298,7234
AKTVPYGLVAPAL	13	2,16E+03	1298,7598
VEAGVGLFVDAQVSAATH	18	2,16E+03	1769,8948
VEAGENLVSVLGKEVNN	17	2,16E+03	1769,916
LLPAYSNSPKLLYLVQ	16	2,15E+03	1818,0291
VVDQ(+,98)N(+,98)GNSVFC(+57,02)ALVA	15	2,15E+03	1593,7344
GSATPWLGSLMGEGAGGLVQ	20	2,15E+03	1886,9197
LDVVVMVSVPPE	12	2,14E+03	1282,6843
DVLVVMVSVPEP	12	2,14E+03	1282,6843
LAAFVAVSAKPV	12	2,14E+03	1171,6965
ELAAPLKPPGDVLL	14	2,14E+03	1431,8337
NVFLVDKKPL	10	2,13E+03	1171,6965
LYHAAAGYLALPEGL	15	2,12E+03	1557,8191
MLVLPTPTLYSVPQ	14	2,12E+03	1557,8477
LANGVFLHEVVLM(+15,99)	13	2,12E+03	1456,7749
LLESFSLTQGGAL	13	2,11E+03	1334,7083

DALYNVVAC(+57,02)LPVLV	14	2,11E+03	1544,8271
HC(+57,02)GEWFFPGLGRQ	13	2,10E+03	1589,7197
EAPGEWM(+15,99)FPGLLDL	14	2,10E+03	1589,7437
LASANSVNSEVVPE	14	2,10E+03	1414,6938
AFLVVDNPLFDNFFPPTN	18	2,09E+03	2066,0149
AWLPTQSSVKC(+57,02)GRGR	15	2,08E+03	1701,8733
KFLAHASK	8	2,08E+03	900,5181
HTLVLNFTT	9	2,08E+03	1044,5603
HTTTFTLKP	9	2,08E+03	1044,5603
GAGKLKSRE	9	2,07E+03	944,5403
NPPELKNLFHDN	12	2,07E+03	1436,7048
NKLWERPLHLGFLT	14	2,07E+03	1722,957
NGHQ(+,98)LGGSTRLDF	13	2,07E+03	1401,6636
GPGQEVFGQGHPHQQVR	17	2,07E+03	1856,9031
SALSGFTLEDVGDK	14	2,06E+03	1437,6987
QTGVLRKFV	9	2,03E+03	1046,6235
MFFLPFF	7	2,03E+03	947,4615
NPTKSFKTMAPLL	13	2,01E+03	1446,7905
VM(+15,99)M(+15,99)STAEVM	9	2,01E+03	1029,4182
ADLALLHALALNA	13	2,00E+03	1304,7451
PVNPGMLSTAFL	12	2,00E+03	1245,6428
APGDAVGVLPHADHL	15	1,99E+03	1467,7471
MVVFAKALFV	10	1,97E+03	1123,6462
AGELTLGVTLNP	12	1,97E+03	1183,6448
SVHPLPSAAPGGR	13	1,97E+03	1244,6626
VPNNVTLPVL	10	1,96E+03	1064,623
VVPDDAMSVM(+15,99)MQ	12	1,96E+03	1337,5667
YSSSVSPPASADGN	14	1,96E+03	1337,5735
APLGGNPKA	9	1,96E+03	823,4551
ASKTHPAL	8	1,96E+03	823,4552
ASKHPTAL	8	1,96E+03	823,4552
AVPDARTVSYVK	12	1,95E+03	1304,7087
AVPN(+,98)AGPPANVAVK	14	1,95E+03	1304,7087
QTAGGTQMELRG	12	1,95E+03	1247,5928
EVAPSYSTLGPQ	12	1,95E+03	1247,6033
LGSN(+,98)PSAGPGL	11	1,95E+03	969,4767
HEVAAVYYPNTFE	13	1,94E+03	1538,7041
RHDHELAAL	9	1,94E+03	1060,5413
FNDFLGPV	8	1,93E+03	907,4439
FNDFLRP	7	1,93E+03	907,4551
PSGGPM(+15,99)SSN(+,98)VNEE	13	1,93E+03	1320,5139
PAAEYVRQL	9	1,93E+03	1045,5557
YTHLQLFPGMWME	13	1,92E+03	1651,7527
AGGELKAPKMSAH	13	1,92E+03	1295,6655
SLEDAKRLKFAAAQ	14	1,92E+03	1546,8467
NQFGLTFSPTAHLGQ	15	1,91E+03	1616,7947
KKASANFLFGLF	12	1,90E+03	1341,7444
EGKPVRAY	8	1,90E+03	918,4922
SVKPTAGFL	9	1,90E+03	918,5175

GNQEN(+,98)Q(+,98)FQKF	10	1,90E+03	1240,5359
DVFVPQ(+,98)VVGGLK	12	1,90E+03	1257,6968
DRQVGLPHWNLN	12	1,89E+03	1447,7319
GSSVSLDGNSPVVAN	15	1,88E+03	1401,6736
LNPMLLM(+15,99)TAKL	11	1,88E+03	1259,6982
LNASTSVAGTKVL	13	1,88E+03	1259,7085
EGFMRHFDGQ	10	1,87E+03	1222,5188
GMVNSHNLPLLRLAG	15	1,87E+03	1590,8665
TSPQHMVKSLR	11	1,86E+03	1282,6816
PVLPVTVPVDSSPVAHA	17	1,86E+03	1683,9194
SPEC(+57,02)GVLWLM(+15,99)WPY	13	1,86E+03	1652,7368
M(+15,99)MVSM(+15,99)LAESPLHLY	14	1,86E+03	1652,7612
YLDSKHNPY	9	1,85E+03	1135,5298
QYKVEQGNTADVTTDGQKSGFVK	23	1,84E+03	2499,2241
GDDYTC(+57,02)M(+15,99)EGPY	11	1,84E+03	1322,4431
VFVLGGPEVV	10	1,83E+03	1014,575
DFAPYHGKDNQQWSLR	16	1,83E+03	1960,918
YYKVVLDVKESDATVGVGLL	20	1,82E+03	2167,1775
YNEGDDEY	8	1,82E+03	1003,3406
EAPVEQ(+,98)QEGAVG	12	1,82E+03	1213,5461
LQC(+57,02)LRDLPGGPDTF	14	1,82E+03	1587,7715
M(+15,99)GAASVHFANGK	12	1,82E+03	1204,5659
NLNNPSDSDLFNPR	14	1,81E+03	1601,7434
FLDSATNFLAHQM(+15,99)	13	1,81E+03	1509,6921
LFDSDSPNNLLFE	13	1,81E+03	1509,6987
EPNPSVMAPGDNLF	14	1,80E+03	1486,6763
PETGLMRLQ	9	1,80E+03	1043,5435
AGVN(+,98)Q(+,98)QLAGVK	11	1,80E+03	1085,5715
YNYDEY	6	1,80E+03	865,313
QESAPHGGPHHQKVR	15	1,80E+03	1663,8291
DGANHRMMFLAQ	12	1,80E+03	1389,6282
NNQGLVFDPSNLQPE	15	1,80E+03	1670,79
KGNALLAAAP	10	1,80E+03	924,5392
EVAGN(+,98)HSLNHNLA	13	1,79E+03	1375,6479
QLSPVTYC(+57,02)QLPA	12	1,79E+03	1375,6807
PYFLNDPSPSDLFNVPG	17	1,78E+03	1877,8835
PLAWLPTQSWKPGVAYV	17	1,78E+03	1912,0247
QLGTSVHGGGHVSHLN	16	1,78E+03	1598,7913
KNNAASPGGPHSVHLN	16	1,78E+03	1598,7913
KLLVGEELM(+15,99)KPAPA	14	1,78E+03	1510,8428
NRKGVLVGPN	10	1,76E+03	1052,6091
YVFVLVGPGDL	11	1,76E+03	1177,6382
YVFVPEPKSL	10	1,76E+03	1177,6382
LWFN(+,98)KC(+57,02)M(+15,99)TEAHA	12	1,75E+03	1523,6538
DHSLPTKSWKPVPRH	15	1,75E+03	1783,9482
FGKNSPKLLY	10	1,74E+03	1165,6494
VDFLEPVGMADM(+15,99)L	13	1,74E+03	1451,6677
FEFLPLMLK	9	1,74E+03	1136,6304
YPLSVKGSGKNLP	13	1,74E+03	1358,7559

LLGDVDAPATVFDGQ	15	1,73E+03	1516,741
VALLEN(+,98)HM(+15,99)LGPALF	14	1,73E+03	1540,7959
REAREEEAQVG	11	1,73E+03	1272,6057
GDN(+,98)SLEGNLFVK	12	1,71E+03	1292,6248
PKEQVKKPEVK	11	1,71E+03	1308,7764
MGGASAGQAVM(+15,99)TP	13	1,71E+03	1192,5217
LLKPGNML	8	1,70E+03	884,5153
ENMLWNLYYKVVL	13	1,70E+03	1683,8694
EQLPTAGSWKPVNLY	15	1,70E+03	1701,8726
QQEQHQKLGV	10	1,70E+03	1193,6152
NGTVKEN(+,98)TLYLNL	13	1,69E+03	1478,7617
KNMNGTTGDLNGQGGENL	18	1,68E+03	1818,8167
LNLN(+,98)SLEC(+57,02)LLHVF	13	1,68E+03	1571,8018
LKATPVGPP	9	1,68E+03	878,5225
PGGKAKKPP	9	1,68E+03	878,5338
PYGLPLLKADGLGKAQ	16	1,68E+03	1639,9297
TTLGASAAKGLPVEVLAN	18	1,67E+03	1710,9517
KNLVSERPLHLGF	13	1,67E+03	1508,8462
NSNNKLVTGDPGLM(+15,99)DPGC(+57,02)AL	20	1,66E+03	2087,9614
PPTPGVVVLMH	11	1,66E+03	1145,6267
AN(+,98)GQNLRLT	9	1,66E+03	986,5145
NLEGASANSFYK	12	1,66E+03	1299,6094
VSGGVPEVLGTK	12	1,65E+03	1141,6343
PGGLALPPGPP	11	1,65E+03	971,5439
RVGSPKPQREQSRL	14	1,65E+03	1636,9121
FFFENLFLKGLYY	13	1,65E+03	1699,865
ALKSM(+15,99)PEEVVAH	12	1,65E+03	1325,6648
ALKSQGMGEVVAH	13	1,65E+03	1325,676
LFKGSDKNPAAGSL	14	1,64E+03	1403,7407
AVNMVVSPVLLA	12	1,64E+03	1211,6948
QNEEWFFPGLVNEQ	14	1,63E+03	1735,7842
LDLTTKPVSGN	11	1,62E+03	1143,6135
TKGVLPPGPAA	11	1,62E+03	1006,5811
FDKLVVFN	8	1,62E+03	980,5331
GDN(+,98)SHGEGGLF	11	1,62E+03	1089,4363
NLDQ(+,98)QLAGVKLQ	12	1,62E+03	1326,7144
VVLDVKESDAAASSP	15	1,62E+03	1486,7515
LEVGKLVGNPLY	12	1,61E+03	1300,739
QLLTALVGNPLY	12	1,61E+03	1300,739
PEEPTAPAP	9	1,61E+03	907,4286
MAAKPTM(+15,99)LK	9	1,61E+03	1005,5351
GLQPGMGM(+15,99)WGTE	12	1,61E+03	1278,5374
VAQPSC(+57,02)APASYE	12	1,61E+03	1278,5549
MFKGEQVQGDFVSDVNYLF	19	1,60E+03	2222,0354
VVARLVGPN	9	1,60E+03	923,5552
FGLDADANQN	10	1,59E+03	1063,457
PEEVVHVQ	8	1,59E+03	935,4713
PEERLGVH	8	1,59E+03	935,4824
LQAAAHPTGQHL	12	1,58E+03	1242,647

KPVAAGMTFPGAPTTF	16	1,57E+03	1591,8069
FGGEPWGGEGL	11	1,56E+03	1104,4875
KTVPGHGSLS	10	1,56E+03	981,5244
TKVPGHGSLS	10	1,56E+03	981,5244
VVPPGTPVVE	10	1,56E+03	992,5542
VFVVVVSSGT	10	1,56E+03	992,5543
VFVVFGVLN	9	1,56E+03	992,5695
LLGGVYAMVGDVHL	14	1,56E+03	1442,759
NNASDLFNHEPP	12	1,56E+03	1353,595
ADSSAC(+57,02)C(+57,02)AR	9	1,55E+03	996,3753
GATHAGGQDSSR	12	1,55E+03	1142,5063
GADFNKKAPVSPVENQ	16	1,54E+03	1699,8528
LLGLNGGC(+57,02)GPLWAR	14	1,54E+03	1482,7766
MHKKTDTESLGFELQHGGQ	19	1,54E+03	2142,0164
MKLPSAFK	8	1,53E+03	920,5153
NLVASFPAVGAPN	13	1,53E+03	1255,656
NLDHSVPLPGLTNLP	15	1,52E+03	1585,8464
HATSLNEEMSKFQ	13	1,52E+03	1520,6929
MLKVFSGKKGDTPV	14	1,51E+03	1505,8276
M(+15,99)C(+57,02)NHSNY	7	1,51E+03	940,3168
GDDSHQDGLY	10	1,51E+03	1105,4312
SHNLRAKAAKSGLAAPL	17	1,50E+03	1703,9795
NFLVLVKG	8	1,50E+03	888,5433
NFLVLRK	7	1,50E+03	888,5545
TADSLGENLGLNQL	14	1,50E+03	1443,7205
GDDSLWNLVGGPSG	14	1,49E+03	1372,626
C(+57,02)HQLLLLGGSLLME	14	1,49E+03	1582,821
KRLVGPN	7	1,49E+03	782,4763
EEGEFFPGRAVQ	12	1,48E+03	1364,636
QPAYLYGV	8	1,48E+03	909,4596
GQKGAYVK	8	1,48E+03	849,4708
GQKGSFVK	8	1,48E+03	849,4708
FFELRPGLALEP	12	1,48E+03	1387,75
YSNSPKLLYR	10	1,48E+03	1239,6611
MLKLSFSSSK	10	1,47E+03	1126,6057
HEQFGKGFGLTTTEHGKNA	19	1,47E+03	2057,9919
LLEN(+,98)PGTAM(+15,99)LL	11	1,47E+03	1187,6108
VTPQNFVVVRK	11	1,46E+03	1285,7507
LDTAAPTALPEAVL	14	1,46E+03	1380,75
LLQGKKSGEV	10	1,46E+03	1057,613
LLKVFSGTPP	10	1,46E+03	1057,6172
NAEPQKWSLVG	11	1,45E+03	1227,6248
Q(+,98)MPGQPASSLYTY	13	1,45E+03	1442,6387
YVLVVVPGPTPDL	13	1,44E+03	1367,77
PEEVVAHL	8	1,44E+03	892,4654
LKKPVLVPN	9	1,43E+03	1006,6539
YNLDESM(+15,99)FLTPENLM(+15,99)	15	1,43E+03	1847,7957
DEAVRLPHWNLN	12	1,43E+03	1462,7317
FFNN(+,98)FLGPV	9	1,42E+03	1054,5125

NAVLTLPKNFLVGLA	15	1,42E+03	1568,929
VFVPEVGVGLR	11	1,42E+03	1170,676
PLM(+15,99)KTHVL	8	1,42E+03	953,5368
VARLPGGGADKNLN	14	1,42E+03	1380,7473
DTTNSDNQLN(+,98)LNPR	14	1,42E+03	1601,728
DVFVPEVATGPK	12	1,41E+03	1257,6604
METTDNTAVPDATTLQ	16	1,40E+03	1706,7668
LLM(+15,99)GEPYHSVQQNAGSV	17	1,39E+03	1844,8726
DLGVGGVYVK	10	1,39E+03	1005,5494
NNPAKATNTSVPY	13	1,38E+03	1375,6731
KVNTSLPGPTVLM	13	1,38E+03	1355,7483
HHHSGKAGHAGPTVGNGTPQ	20	1,38E+03	1945,9255
DGDSLSVLNQL	11	1,38E+03	1159,572
VGN(+,98)LHEEASTYTM(+15,99)	13	1,37E+03	1467,6187
PTPGVPVVM(+15,99)LVE	12	1,37E+03	1252,6736
GALWPSPHDVQ	11	1,37E+03	1205,583
LVVEVNQTNALP	12	1,37E+03	1295,7085
LFFDM(+15,99)KMPE	9	1,37E+03	1172,5247
SSALSEGALAQ	11	1,37E+03	1032,5088
C(+57,02)PLPTGYM(+15,99)SSQGPM	14	1,37E+03	1540,636
PN(+,98)ATRAD	7	1,36E+03	744,3402
NPSN(+,98)SN(+,98)LFNPR	11	1,36E+03	1260,5735
LLM(+15,99)WPYHGKLALGDVSLVC(+57,02)	19	1,36E+03	2187,1221
KVPKVGLVGPN	11	1,36E+03	1106,6812
PGLPKNRPPT	10	1,36E+03	1075,6138
HVGVAPNSATGYQAF	15	1,36E+03	1517,7263
KVVLDVQESDAARW	14	1,35E+03	1614,8364
PGSEFAVGVEFASLH	15	1,35E+03	1545,7463
LAVLGGALVLGH	12	1,34E+03	1118,6812
NSLPLRPH	8	1,34E+03	932,5192
EDPYM(+15,99)LDM	8	1,34E+03	1028,3831
AAPLVYGVLGF	11	1,34E+03	1105,6172
HANEPQKWSAVV	12	1,34E+03	1364,6836
HKGENKGAPAATKG	14	1,34E+03	1364,7161
VGSLYVTR	8	1,33E+03	893,4971
EELFGGPPNGTL	12	1,32E+03	1229,5928
VTPPLTLY	8	1,32E+03	902,5113
SFLRPVNLPGQTSLPE	16	1,32E+03	1753,9363
SFLRPVNLPGKHSRM(+15,99)	15	1,32E+03	1753,9409
AFPPSYALLALPESADL	17	1,32E+03	1773,9187
PM(+15,99)LALLADSGAKSV	14	1,32E+03	1387,738
LHSAKVVL	8	1,31E+03	865,5385
GWTVPGGGEEETFNAF	16	1,31E+03	1696,7368
VVLSYPHLY	9	1,31E+03	1089,5859
VSAPGAPTR	9	1,31E+03	854,461
TQ(+,98)TANQNNKLSGPPAFNQDATPP	23	1,31E+03	2411,1353
HMGPPTFVPLVGNY	14	1,31E+03	1527,7544
NAKGSFVKL	9	1,30E+03	962,5549
HGKENQKWGL	10	1,30E+03	1195,6099

FFYVSLGRNYLAVY	14	1,30E+03	1710,877
NQLDQNHVGDFFL	13	1,30E+03	1545,7212
WALPTVFVPGDGVLL	15	1,29E+03	1582,876
WALPTVFVGKAAAPR	15	1,29E+03	1582,8984
QAPVFLLTPNL	11	1,29E+03	1211,6914
GTVSLTGHVL	10	1,29E+03	982,5447
ETSVGGM(+15,99)TKVLA	12	1,28E+03	1207,6118
LNGGGGSPLVLV	12	1,28E+03	1081,613
YFGGQPSSKFSTY	13	1,28E+03	1467,667
AAEPDYLV	8	1,27E+03	876,4229
SNGKLSM(+15,99)TADSL	12	1,27E+03	1238,5813
YFGELGLGTPPQFK	14	1,27E+03	1552,7925
ARVLPHADNLGGAH	14	1,27E+03	1426,7429
PKTLPHWNLGGAH	13	1,27E+03	1426,7468
AVRLPHSVNLGGAH	14	1,27E+03	1426,7793
QATVQAGLLASLSAR	15	1,26E+03	1484,8311
QTGVLRKSSGA	11	1,26E+03	1102,6094
YVNGNQKVSAER	12	1,26E+03	1363,6843
LGPTLGMADVLEWVSF	16	1,26E+03	1733,8699
LTGHLLGDGAPVK	13	1,26E+03	1276,7139
NGNAMWNLYYKVV	13	1,25E+03	1570,7603
FGPYVLGDYMAPWD	14	1,25E+03	1629,7173
LNEGHLPPTGVPGAL	15	1,25E+03	1470,783
LHRKGRTA	8	1,25E+03	937,5569
NPPVDLVVALHD	12	1,24E+03	1287,6824
DSGLVLAEAF	10	1,24E+03	1020,5128
RAEEEAGAR	9	1,24E+03	987,4733
NLESALVSM(+15,99)Y	10	1,23E+03	1141,5325
NAPKRLVGPN	10	1,23E+03	1064,6091
PGKETGPPGPDTHQLRN	17	1,23E+03	1799,8914
TTTGPTM(+15,99)TAVPDADAAVS	18	1,23E+03	1720,7825
TAVPERTR	8	1,22E+03	928,509
VLM(+15,99)PNGNSVFDGRW	14	1,22E+03	1606,7561
LADPSSATLKGATEVGASPS	20	1,21E+03	1857,9319
KSDTLFFQV	9	1,21E+03	1083,5601
KSDTLFM(+15,99)VK	9	1,21E+03	1083,5635
AAPGSFVKLL	10	1,21E+03	1001,5909
VDFVVLDGAPVV	12	1,21E+03	1228,6704
SSELKSAPLGPN	12	1,21E+03	1198,6194
ETVSQRVLGPN	11	1,21E+03	1198,6306
FM(+15,99)PGVPGPLQRQ	12	1,21E+03	1341,6863
QHPPTTHQ	8	1,20E+03	944,4464
MLAGAKAVSGDFVSNGNPPLM	21	1,20E+03	2075,0178
NQKNLNANVP	10	1,20E+03	1110,5781
FYQ(+,98)LVFASWM(+15,99)L	11	1,20E+03	1420,6736
NLPDLAFWWYP	11	1,20E+03	1420,6816
NQLDFFRGMQTL	12	1,20E+03	1468,7134
VEALAKPPLAHA	12	1,20E+03	1215,6975
WVTLKELPSLW	11	1,20E+03	1370,7598

PKALWNGLLLPSYSNAPQLF	20	1,20E+03	2228,1992
FPVGSGENLVLQL	13	1,20E+03	1371,7397
VDC(+57,02)LWNLGLLPKL	13	1,19E+03	1539,8484
YVTRGGSRVQ	10	1,18E+03	1121,5942
FAGFLAQGGL	10	1,18E+03	979,5127
SSFN(+,98)LGEN(+,98)PPAHLM(+15,99)	14	1,18E+03	1530,666
DSFVVSVLDTTVSLYY	16	1,18E+03	1806,8928
EMEHKNKFGLVHPVAQQHGGAG	22	1,17E+03	2370,165
SEAFNVDEQLVRKL	14	1,17E+03	1646,8628
EAFNLDEQLARLK	13	1,17E+03	1545,8149
NSNNKLDSTGSSGPGPTNVSK	21	1,17E+03	2059,9771
VVVPGPTPFPGV	12	1,16E+03	1164,6543
VPLNPSDSDLFNPR	14	1,15E+03	1569,7786
GVGAATALGH	10	1,15E+03	852,4454
VGPPTPAKSVVYP	13	1,14E+03	1310,7234
EGGPPTLEHGAPL	13	1,13E+03	1273,6301
NSAVLLTPAR	10	1,13E+03	1040,5979
WLPTN(+,98)VFVPSLTPR	14	1,13E+03	1626,877
VYVSLLAAASPP	12	1,13E+03	1186,6597
PQEEDAFFGYFL	12	1,13E+03	1461,6453
VNQGLVKEPVFSF	13	1,12E+03	1462,782
GVQNSQ(+,98)Q(+,98)AGAVG	12	1,12E+03	1116,5046
LDSMKTDALFAETF	14	1,11E+03	1587,749
LWTNGVN(+,98)LPGGM(+15,99)MAETF	17	1,11E+03	1853,8328
LWNTGVDLPGGM(+15,99)MAETF	17	1,11E+03	1853,8328
QNGLGEEEEDPPTT	14	1,11E+03	1514,6372
QGGSNVQFGLYVNF	14	1,11E+03	1528,731
VNGGPTPPP	9	1,11E+03	834,4235
PLVNGAAGH	9	1,11E+03	834,4348
EATLGSM(+15,99)M(+15,99)PGAV	12	1,10E+03	1194,5261
SHLLESADK	9	1,10E+03	998,5032
GSDAVMKPALM(+15,99)ADPYHGKDNQKMALRA	27	1,10E+03	2930,4199
PAYSNSPKLLY	11	1,10E+03	1251,6499
NQLDLMGNAPALVNL	15	1,10E+03	1581,8184
GDDSLWNLQALL	12	1,10E+03	1343,6721
LDAVLTENHNGVT	13	1,10E+03	1381,6838
LAPVPEALGSMTVGQ	15	1,09E+03	1468,7595
TTGAAAGAGPP	11	1,09E+03	869,4243
WADVSVQTLGTGGLN	15	1,09E+03	1516,7522
MAGLVSVVEGDGKAAN	16	1,09E+03	1516,7556
LM(+15,99)VYPYC(+57,02)GLDMPETY	15	1,09E+03	1866,7878
LTGVARANKS	10	1,09E+03	1015,5774
NNLM(+15,99)PGAPADAPPFDTGLDLGG	22	1,09E+03	2154,989
DHAPSSALGGLF	12	1,08E+03	1170,5669
EPKMALTYLY	10	1,08E+03	1227,6208
GAPGTSLLGP	10	1,08E+03	868,4654
LGESTTDAGV	10	1,07E+03	948,44
EDAGPSLAGLSM(+15,99)	12	1,06E+03	1162,5176
FMYSGLVLSM(+15,99)	10	1,06E+03	1162,5403

ALSAGGALLQ(+,98)KENEYVKA	18	1,06E+03	1861,9785
ALSGGAALLEKEGGEYVKA	19	1,06E+03	1861,9785
PKAWPSHGEGVP	12	1,06E+03	1260,625
SDLVTGGSVVSF	12	1,06E+03	1166,5818
LGQ(+,98)LKRLVGNP	11	1,06E+03	1194,7085
GVAVPDATRW	10	1,06E+03	1070,5508
MAGKPLAPGGAR	12	1,06E+03	1124,6123
FFELC(+57,02)ASSAVML	12	1,06E+03	1373,636
MYWGPFLLPANTK	13	1,06E+03	1536,7798
QPPKGFTFPGSVLY	14	1,06E+03	1536,7976
VGNAGANGLP	10	1,06E+03	868,4402
QTAKTLARSRESP	13	1,05E+03	1443,7793
AGPASPGVPVH	11	1,05E+03	987,5137
QALAPVYVGA	10	1,05E+03	987,5389
PETSRTLLT	9	1,05E+03	1016,5502
NKGAPTNLPLLQ	12	1,04E+03	1264,7139
WRESVSNTPVDLMW	14	1,04E+03	1718,8086
VSLEREGGV	9	1,04E+03	944,4927
ANENQKVSEAR	11	1,04E+03	1244,6108
GQEGGQKVSEAR	12	1,04E+03	1244,6108
EVNAHHGVRVQ	11	1,04E+03	1244,6375
HGQPNQKVSATAL	13	1,04E+03	1349,7051
GGNGVLTLVGPN	12	1,04E+03	1096,5876
LM(+15,99)HGKENQKVSPKM	14	1,04E+03	1641,833
KASNKGLEWVSFK	13	1,04E+03	1492,8037
PKDEWSGPD	9	1,03E+03	1029,4403
PEDQWMPK	8	1,03E+03	1029,459
PENQMRGAQ	9	1,03E+03	1029,4662
YDDAAVVTYLSM(+15,99)	12	1,03E+03	1362,6013
AEVVEGM(+15,99)HELSF	12	1,03E+03	1362,6125
HFKGPQKGAGKTH	13	1,03E+03	1391,7422
NLDNLVGVPLGDLL	14	1,03E+03	1450,8032
GEVFVPVTSALVP	13	1,02E+03	1313,7231
VVATHPSSAM(+15,99)V	11	1,02E+03	1113,5488
LHLSVYMPTQPMWPY	15	1,02E+03	1861,8894
VDTVAVSVGSVAP	13	1,02E+03	1199,6396
RLTEWGTLR	9	1,02E+03	1130,6196
ALLLSKHPNLSAS	13	1,02E+03	1349,7666
MLKVVGGQPL	10	1,02E+03	1040,6052
LGKKFGVGL	9	1,02E+03	917,5698
DLPEWFFPVALNN	13	1,01E+03	1560,7612
NSNNQTTGLAGPGGLMGC(+57,02)LAQ	21	1,01E+03	2059,9414
PMAPVPEGHLNVEAAAQ	17	1,00E+03	1729,8457
AGAEPAQAPQ	10	9,96E+02	938,4457
M(+15,99)SVPNGEGK	9	9,95E+02	933,4225
VNSARLLPLGEL	12	9,93E+02	1280,7451
VAKQTEKTHK	10	9,92E+02	1168,6565
GAGSVKEDTLYLAK	14	9,91E+02	1450,7666
MNKPAVQAVATP	12	9,88E+02	1225,6489

LGLLPAYSNSKLPLYLV	17	9,88E+02	1860,0759
MLLAVC(+57,02)VEPGREV	13	9,87E+02	1471,7527
VDFLPTAGPKAEEV	14	9,87E+02	1471,7559
RGVPGPHLY	9	9,86E+02	994,5348
MFNGFALALVNP	12	9,86E+02	1292,6587
QVTESSDNQLDLNPR	15	9,83E+02	1714,8123
LDTTNSN(+,98)NQLDLNPR	15	9,83E+02	1714,8123
QN(+,98)AYLYR	7	9,83E+02	927,445
EGLPTVFVTVTPPR	14	9,79E+02	1511,8347
VSADGTFAQAVLM(+15,99)	13	9,79E+02	1324,6333
LC(+57,02)DVHELEFGYDDL	14	9,77E+02	1723,74
YYKVVLR	7	9,77E+02	939,5541
DVAPPRVPAADLL	13	9,77E+02	1332,74
QTGAAPTGLPHVSHL	15	9,74E+02	1484,7734
EGLPSLFVE	9	9,74E+02	989,5069
YKVVLDVK	8	9,72E+02	962,58
LSRENLADSNPLDVF	15	9,71E+02	1688,8369
KGEAAALLEGYM(+15,99)PKAVKA	18	9,70E+02	1861,9971
LPVEGHPPMLLY	12	9,68E+02	1364,7163
VLMVVSPPAPVE	12	9,68E+02	1236,6787
LVMFVALM(+15,99)AVK	11	9,68E+02	1236,6975
YTGSGVSGFTFAEV	14	9,66E+02	1420,6511
SSVTWAGQRGEYVK	14	9,65E+02	1566,7791
VPETSSAVLL	10	9,60E+02	1014,5597
VFVPEVGRL	9	9,59E+02	1014,5862
RSPMSSK	7	9,54E+02	791,3959
LGLLPQYVNGTKAGGSLVVK	20	9,52E+02	2013,1621
LLGLQPYVNGPKLLYVVQ	18	9,52E+02	2013,1663
VVDENGNWFDQLK	13	9,52E+02	1562,7366
N(+,98)VPKEVKVLAVGNP	14	9,50E+02	1463,8347
ALKSMPEEVVAHAF	14	9,50E+02	1527,7754
TAQSGPLKYT	10	9,49E+02	1064,5503
MFGQKALEFGYDDL	14	9,49E+02	1632,7493
KTGVGRGGQVGLP	13	9,49E+02	1224,6938
EGSKHQAM(+15,99)SLSVNL	14	9,48E+02	1515,7351
MAGSLPGASSLLGELN	16	9,48E+02	1515,7603
GPM(+15,99)LAGTGGSC(+57,02)DKLH	15	9,48E+02	1515,6809
KTGPPAMVTAL	11	9,45E+02	1084,595
VQVAYQARLQ	10	9,44E+02	1174,6458
NEHM(+15,99)QMQGYGQLNGVLN	17	9,42E+02	1947,8567
PLDVAR	6	9,42E+02	669,3809
VVLNVSPEEVVANAF	15	9,41E+02	1585,8352
EAVATQPSWGNSLPLN	16	9,40E+02	1682,8264
AVPN(+,98)ATSWKTVSELG	15	9,39E+02	1559,7832
M(+15,99)VVPEPVQV	9	9,39E+02	1012,5263
TLLNPAAGTLVL	12	9,39E+02	1181,7019
KTGDFVVLF	9	9,38E+02	1024,5593
NAGLALKAGNP	11	9,38E+02	1024,5665
MVLPTPVVE	9	9,37E+02	983,5361

MPATGHALGHDVK	13	9,37E+02	1332,6609
PWLLHPSNAAPGLFYVVQ	18	9,35E+02	2008,0571
LLGLEFASNAPQLFYVVQ	18	9,35E+02	2008,0669
THALTHGVVGH	11	9,31E+02	1127,5835
LPTVAAHEPPP	11	9,31E+02	1127,5974
SYYYYSAVVSGLEWATTN	18	9,31E+02	2072,9368
PTVLLVM(+15,99)P	8	9,30E+02	884,5041
C(+57,02)HALPTLSVNAGGWFQ	16	9,27E+02	1756,8354
VFVPVFGLGPD	11	9,23E+02	1145,6121
MKFANAVVEEPGMRVAP	17	9,17E+02	1844,9277
QAWLPTFVKLL	11	9,17E+02	1314,77
FGLNADNVPNSGK	13	9,15E+02	1331,647
PGHVGNSQDSSRNLFS	16	9,11E+02	1700,7866
YVVAGETSGPGGRM	14	9,11E+02	1379,6504
PMKELKGDADKP	12	9,10E+02	1327,6804
AALLEKEGGEYVK	13	9,07E+02	1405,7451
MVDHTSFLA	9	9,04E+02	1019,4746
EQLGVSGSNALGENLQ	16	9,03E+02	1614,7849
NDAVRLPHWHLN	12	9,02E+02	1470,748
TLNKYATLTLNSSGR	15	8,99E+02	1637,8738
LTLEYNLPSVAHGPK	15	8,99E+02	1637,8777
YVFVGGLNKAW	11	8,95E+02	1252,6604
LKSPTPPGVVVLM(+15,99)	13	8,95E+02	1352,7737
AVPDPKGTM	9	8,93E+02	914,4531
PEEVVAHAD	9	8,92E+02	965,4454
FPKGKGLAKM	10	8,88E+02	1075,6211
WALQ(+,98)PDTGAGPK	12	8,85E+02	1240,6086
NVSLAVSVADKPAKN	15	8,81E+02	1511,8308
PETLSTRLW	9	8,81E+02	1101,5818
PNVTPLVLH	9	8,79E+02	988,5706
LDQNHLASFM(+15,99)L	11	8,78E+02	1303,623
VPRSVSQPV	9	8,77E+02	967,545
FFLKPGDVAR	10	8,76E+02	1148,6343
SRSVPGPLGTQGG	13	8,75E+02	1211,6257
VEHN(+,98)LN(+,98)LVAPERS	13	8,74E+02	1478,7366
YAGGHAGPY	9	8,73E+02	891,3875
ELGATSGLSPFAGVP	15	8,72E+02	1401,7139
LGDFLPQAFAPL	12	8,71E+02	1287,6863
PVDELTFSGPR	11	8,71E+02	1216,6086
RHNLNNDSGSH	11	8,68E+02	1249,5547
RSGNTFDNAHPGMWPETF	18	8,66E+02	2062,8955
LLSGRLVGPNSVAE	14	8,64E+02	1410,783
SPDGGTM(+15,99)GAVGLVGM(+15,99)SSLY	19	8,63E+02	1829,8176
DGGGVLALVATL	12	8,62E+02	1084,6128
LVDVVFHAL	9	8,60E+02	1011,5753
YMNSVDTDVKALP	13	8,59E+02	1451,6965
Q(+,98)SAGGPGKEPGPGTPPHQQH	20	8,57E+02	1963,9136
AHLKALSGAGTAPYLY	16	8,57E+02	1631,8672
YAN(+,98)SPAELLMRPF	13	8,57E+02	1508,7334

FLPEVASGAL	10	8,56E+02	1002,5386
VAKSC(+57,02)MKVVK	10	8,50E+02	1148,6409
DSDLANNMTN	10	8,48E+02	1093,4346
HGKEPQGAWEKV	12	8,45E+02	1364,6836
LNKGDLDLLTVGAP	14	8,45E+02	1424,7874
HQKVR	5	8,45E+02	666,3925
MLAATVFLAPKN	12	8,41E+02	1274,7056
MLALAFVVGLEL	12	8,41E+02	1274,7307
WALPQLC(+57,02)DNGKKM	13	8,38E+02	1559,7588
VLDVKQ(+,98)MAAAMGPGLL	16	8,35E+02	1613,8521
LQ(+,98)ATSTLRFVVFGPNLDLM	19	8,34E+02	2122,113
LVGGPVVVGPN	11	8,32E+02	1006,5811
VAPKKAMANV	10	8,30E+02	1027,5847
M(+15,99)VLPM(+15,99)PGLYLVT	12	8,29E+02	1364,7085
QGGLDNYKGSNPR	13	8,28E+02	1404,6746
ELLN(+,98)NYPEAQTL	12	8,28E+02	1404,6772
EEAGTANRAEEEAGAR	16	8,26E+02	1659,7449
PTKGEPVVVQ	10	8,25E+02	1052,5867
KELHTKK	7	8,24E+02	882,5287
KNPNMYPSLLE	11	8,23E+02	1304,6436
YMFMPAAGM(+15,99)M(+15,99)M(+15,99)	11	8,21E+02	1327,478
GNPEN(+,98)Q(+,98)FRQQ	10	8,20E+02	1218,5266
VRGAASLKSMPEEVVAH	17	8,20E+02	1779,9302
EMDLGGEEFVANLLL	15	8,19E+02	1648,8018
AALLEEFHSYVAK	13	8,19E+02	1476,7612
AALLEKDGAEYVKA	14	8,19E+02	1476,7822
LPTVVDGHLPPP	12	8,19E+02	1240,6816
NGTVKQ(+,98)DLTPVVH	13	8,17E+02	1407,7358
TLPAALGEVL	10	8,16E+02	982,5699
LPTQSGEKFRSVP	13	8,15E+02	1444,7673
KEVNALLSTAFALPE	15	8,15E+02	1601,8665
LKESVYVK	8	8,13E+02	964,5593
M(+15,99)LPGTM(+15,99)SPVMEC(+57,02)M(+15,99)PETF	17	8,12E+02	2004,801
ESTLAPLSGE	10	8,11E+02	1002,4869
RPFLVLNTRP	10	8,08E+02	1211,7139
LQAGPPDGPKN	11	8,05E+02	1092,5564
ELDGHVQGQTVQ	12	8,05E+02	1309,6262
M(+15,99)EGPYHGK	8	8,04E+02	933,4014
LM(+15,99)TVAEGSVSGKD	13	8,04E+02	1308,623
VGALYGGLGTH	11	8,03E+02	1043,54
GDDSLWNLLVLQ	12	8,02E+02	1371,7034
LEGAPNHALSM(+15,99)M(+15,99)L	13	8,02E+02	1414,6584
PFTVPGPVVPTAVE	14	8,01E+02	1408,7603
LWGADMGGPME	11	8,01E+02	1162,4788
KNDKKWAVSV	10	8,01E+02	1173,6506
HGKVGVKPSEGTAAL	15	7,96E+02	1449,7939
KAPGTSLPH	9	7,93E+02	906,4923
FFHAAYKAAYTLMWEM	16	7,91E+02	1978,9109
LLGLLKPLSNSSEP	14	7,91E+02	1466,8345

SGGSATHADDE	11	7,90E+02	1045,3948
LELTGRHGDPNQ	12	7,89E+02	1335,6531
MPAPTEHAPPW	11	7,89E+02	1232,5647
HGKDPQAGEGTVR	13	7,88E+02	1350,6641
LVDQ(+,98)N(+,98)GNSVFDGSALA	16	7,85E+02	1607,7314
YPQGPDDPE	9	7,85E+02	1016,4086
NGSLHVGFGLASW	13	7,84E+02	1343,6621
GVGAGVAGNHL	11	7,83E+02	950,4933
FLNN(+,98)LFELK	9	7,82E+02	1137,6069
LLLGAGPLALAAC(+57,02)D	14	7,82E+02	1353,7327
EMGTKTPPKY	10	7,82E+02	1150,5693
LGEQ(+,98)TYSSDFMQ	12	7,81E+02	1405,5708
NGKM(+15,99)NRAE	8	7,81E+02	934,4291
SLSVLEGEVSF	11	7,80E+02	1165,5867
LLPQYVDAPLLLY	13	7,80E+02	1516,854
WALPTTLPKPKHALAP	16	7,78E+02	1740,0085
LTLGTASVLGLALN	14	7,75E+02	1341,7866
TSTGPGPPAPLQLQ	14	7,74E+02	1362,7144
MLLALLMNC(+57,02)SAGAPL	15	7,71E+02	1573,803
MAQVTEAARQVVAN	14	7,70E+02	1486,7561
RHSYGAKGGPLMDAM(+15,99)L	16	7,69E+02	1718,8232
VAGNTVMLGDGLYL	14	7,67E+02	1421,7224
LGPQ(+,98)GFALVAL	11	7,65E+02	1085,6121
VSPLKRLVGPN	11	7,64E+02	1178,7134
LLSGSAHAMVNL	12	7,64E+02	1211,6333
RGLHASVGPDGLSL	14	7,56E+02	1377,7363
MLGGEEKMNALP	12	7,55E+02	1288,6155
YSNSPKLLYQ	10	7,55E+02	1211,6187
DAHWHLPGVVANGPV	15	7,55E+02	1567,7896
VVEDDSPSVPPGESPP	16	7,55E+02	1606,7361
FFSGSLDKDVPAYM(+15,99)	14	7,53E+02	1591,7229
EGPAEWM(+15,99)FPGLEPML	15	7,53E+02	1718,7683
KGQ(+,98)AAALLTRENEYVGAALVL	21	7,52E+02	2187,1897
GDN(+,98)SLGELAPVSK	13	7,52E+02	1286,6353
RMRHLEQR	8	7,51E+02	1124,5984
AAQGAAFPP	9	7,49E+02	828,413
DYQADFSGLVSGLEQETTN	19	7,48E+02	2072,9172
HKKPGDKLR	9	7,48E+02	1077,6406
TFVLLAPP	8	7,47E+02	856,5058
AWLPTLSGLL	10	7,46E+02	1069,6172
MLGGANGNLWVQ	12	7,44E+02	1258,6128
NGALDNYPVNL	11	7,43E+02	1188,5774
LELN(+,98)NYGPGKPK	12	7,42E+02	1329,6929
PNGHTKGPGVR	11	7,42E+02	1118,5945
LGGGQDNNGGL	11	7,40E+02	1000,4573
KGPSDKNTSGKKP	13	7,39E+02	1342,7205
LTYVNGPKLLY	11	7,37E+02	1279,7175
SNKGLEWVSFK	11	7,36E+02	1293,6716
QSAPRYLTP	9	7,34E+02	1031,54

PQNFLVLLK	9	7,34E+02	1070,6487
QWYLYGDGATTSKQ	14	7,33E+02	1616,7471
ALPDDPQHA	9	7,25E+02	962,4457
TAVSSMPDR	9	7,25E+02	962,4491
C(+57,02)PPEGEFFPGLGPGMK	16	7,23E+02	1718,7795
FVGAGRVK	8	7,22E+02	832,4919
KVM(+15,99)RGVK	7	7,22E+02	832,4953
VFVPGGNARRA	11	7,21E+02	1142,6309
QASVLPTVQVL	11	7,20E+02	1153,6707
NTPVLDKGFAPL	12	7,18E+02	1270,6921
MDLSVYSL	8	7,15E+02	926,4419
PSLAGPN(+,98)HLAVDVQ	14	7,15E+02	1417,7202
SEASLYGAWLLNGM(+15,99)C(+57,02)KY	17	7,11E+02	1977,8965
FFPMPTL	7	7,08E+02	851,4251
TDTGLVSPHGNATSNVL	17	7,07E+02	1681,8271
YPTYADC(+57,02)Q	8	7,05E+02	1016,3909
YPFSC(+57,02)GTGE	9	7,05E+02	1016,3909
DGQLLGFAKTLGPE	14	7,05E+02	1444,7561
LTELGSNAEHL	11	7,03E+02	1182,5881
LTAAHKVLLAPD	12	7,02E+02	1247,7236
EGC(+57,02)PDTTQ	8	7,01E+02	906,3389
GVGDLLALR	9	7,00E+02	912,5392
NSVHPVGLVAGATH	14	7,00E+02	1357,7102
LLEPTFPANKK	11	6,99E+02	1256,7129
LM(+15,99)DC(+57,02)HC(+57,02)NM(+15,99)	8	6,99E+02	1111,3555
SGPGGPAAPSH	11	6,96E+02	933,4304
LPVLTDGLSAGS	12	6,96E+02	1128,6025
FMWMLKPGAFDPASSASH	18	6,95E+02	1978,907
DADVSTSVVM(+15,99)PVA	13	6,92E+02	1305,6123
LTVFVLAGNPAK	12	6,89E+02	1228,718
NLNTDLRMGLFK	12	6,88E+02	1420,7498
PEVFVVNL	8	6,87E+02	915,5065
PEMSVKLL	8	6,87E+02	915,5099
VVFN(+,98)DKTVVV	10	6,86E+02	1119,6174
PC(+57,02)LLKLLAAN	10	6,86E+02	1111,6423
RVVALGPAAAH	11	6,84E+02	1060,614
RRGGSELFPGESVVSF	16	6,83E+02	1722,8689
WLLGGLGDANLAN	13	6,83E+02	1312,6775
GDGSLVGLM(+15,99)	9	6,81E+02	863,4059
FFEFEGPLNVPGALMV	16	6,81E+02	1765,8748
PM(+15,99)AFFPGLNVQ	11	6,79E+02	1235,6008
EVFLKAAPQNV	11	6,79E+02	1214,6658
VSVM(+15,99)PGSAHVS	11	6,78E+02	1085,5176
EEPSTTME	8	6,78E+02	922,3589
FLAGNPEDQ(+,98)M(+15,99)TLPT	14	6,77E+02	1549,697
NEGGTM(+15,99)KAANVQ	12	6,76E+02	1234,5613
VNNN(+,98)TGFSVSPV	12	6,76E+02	1234,583
LNEEPPHQVQ	10	6,75E+02	1189,5728
NHPQVGADVTPL	12	6,75E+02	1246,6306

SKGAVPPLFLL	11	6,74E+02	1140,6907
VKTVPEQSLPK	11	6,73E+02	1224,7078
EMTAAGPQTVN	11	6,73E+02	1117,5073
QATVVAFLPARVV	13	6,72E+02	1369,8081
EGNDVARYLV	10	6,67E+02	1134,5669
M(+15,99)YNM(+15,99)FM(+15,99)	6	6,66E+02	883,2914
PGHHATTGDDLHPLE	15	6,65E+02	1595,7329
YSNSPKLLYH	10	6,65E+02	1220,6189
VPEVRGLR	8	6,65E+02	924,5505
GVTTGFLLEVGP	12	6,63E+02	1188,6389
LTVDYDAGPPGAEVALK	17	6,62E+02	1714,8777
KSGAHSSNVDPKVP	14	6,61E+02	1421,7263
LTFVPDPRSV	10	6,60E+02	1129,613
GKEAAALLEQEVPNHVS	17	6,59E+02	1790,9163
NQLDNYPPVM(+15,99)GVAPN	15	6,59E+02	1643,7612
TLAGPGSAVLQLGE	14	6,59E+02	1311,7034
WLLLGGPFRSPAVAR	15	6,55E+02	1638,9358
LAFHLLLKGP	10	6,54E+02	1107,6804
SLENYLTVKPGAA	13	6,54E+02	1361,719
SLEGLAALFAKEN	13	6,54E+02	1361,719
MLTWPKGSPELM	12	6,53E+02	1388,6831
GTAELLKF	8	6,52E+02	877,4909
TPAPESSFLAY	11	6,52E+02	1181,5603
VAEETQQLPGNC(+57,02)PMLGTSEGNW	22	6,52E+02	2417,0627
KVVHVSGNPPLLN	13	6,51E+02	1372,7827
KVM(+15,99)MVK	6	6,51E+02	750,4132
GKYVFVALPLEQ	12	6,49E+02	1362,7546
PQNFLVKGVL	10	6,48E+02	1113,6545
DRNM(+15,99)TVLGPGAGVGE	15	6,48E+02	1487,7039
NSSPKLLY	8	6,47E+02	920,4967
FAWTPLAPQA	10	6,45E+02	1100,5654
FPSLGTTKLL	10	6,44E+02	1075,6277
MLKKSVTKAA	10	6,44E+02	1075,6423
LDSHDLDLVAGAVC(+57,02)LP	16	6,44E+02	1693,8345
VGLPHWHLN	9	6,44E+02	1071,5613
EPESVVGVGE	10	6,41E+02	1000,4713
WEYLGGLSGQHVEFGYDDL	19	6,38E+02	2183,98
M(+15,99)STM(+15,99)GVSM(+15,99)	8	6,36E+02	890,3184
HLKPPGVKVKGNRH	14	6,35E+02	1565,9265
LNLPLTSRPLL	11	6,35E+02	1235,76
RALGGLNNFR	10	6,33E+02	1116,6152
VLAASSGNNV	10	6,33E+02	930,4771
LGKDGKKAASKAPA	14	6,31E+02	1340,7776
SKLFVAVGPN	10	6,29E+02	1030,5811
DPSSPNNNTM	10	6,29E+02	1075,4241
GALVGEGVNL	10	6,28E+02	927,5025
YYKVVLDVKESDAGGLW	17	6,27E+02	1940,9883
HGYGLPTSSEAQVGH	15	6,23E+02	1538,7114
GSALN(+,98)TSPFGSL	12	6,21E+02	1150,5505

FPTLLKQHQPVFH	13	6,20E+02	1590,8669
MSVFVTHEGVQ	11	6,18E+02	1232,5859
VM(+15,99)VPALGLSL	10	6,17E+02	1014,5783
YDGAAGLM(+15,99)GGD	11	6,17E+02	1041,4072
LNGGHGGDEGLSSQ	14	6,17E+02	1326,5801
WDVSLPGVLPVAS	13	6,15E+02	1338,7183
EDASQGDAR	9	6,15E+02	947,3944
ATVFPPTPGVV	11	6,15E+02	1083,5964
QTPKTQLAL	9	6,14E+02	998,576
GVTLNAKPGTR	11	6,14E+02	1112,6301
LFEGVQGPE	9	6,12E+02	974,4709
MVVSAEGPLVTQ	12	6,08E+02	1229,6326
LVDQAASYPTK	11	6,08E+02	1191,6135
LTVDYDHGNGATLVL	15	6,07E+02	1586,7939
SHNLKVSVGLLSVPP	15	6,07E+02	1545,8879
NQKWSGVL	8	6,05E+02	930,4923
LVFVPAKGGQL	11	6,05E+02	1127,6702
HGTFLM(+15,99)L	7	6,04E+02	833,4106
LAGSGQGQAATVL	13	6,03E+02	1171,6196
TAVPQTQPPNTGLL	14	6,03E+02	1435,7671
VGTVKFGL	8	6,02E+02	819,4854
VMFLNFFLA	9	6,02E+02	1100,5728
AVRPLHW	7	6,00E+02	877,4922
SLVDNFM(+15,99)RRMGLE	13	6,00E+02	1582,7595
TLNKYNLPFLTHPP	14	5,99E+02	1653,8879
NNLFNHPDGLDVQ	13	5,99E+02	1481,6899
PEEVVAHY	8	5,98E+02	942,4446
DNARNGNSVHAALWP	15	5,98E+02	1620,7756
LTKC(+57,02)GMATTQAVGEGGK	17	5,96E+02	1707,8284
TNDTGVLGVAPSVSLYAVKVL	21	5,95E+02	2102,1621
PANPTGASSVGAHPGPVV	18	5,93E+02	1613,8162
LFM(+15,99)PGVPTGPAKPQ	14	5,92E+02	1454,759
LSVDYDTGLNHGVAL	15	5,91E+02	1572,7783
FMM(+15,99)DPADY	8	5,91E+02	1004,3619
NRC(+57,02)GMFAVMPM(+15,99)ENLFS	16	5,88E+02	1918,8198
WALSPLVFDGVMNQN	15	5,85E+02	1689,8184
ETGVLHNVGTQ	11	5,85E+02	1153,5728
PFAATLPTA	9	5,84E+02	887,4752
PPPRGLDAPLL	11	5,84E+02	1144,6604
ATVPNGQTSTVL	12	5,83E+02	1186,6194
LSAM(+15,99)NLN(+,98)Q(+,98)QLAGVKL	15	5,82E+02	1616,8442
LSAFNLNTPVDLASR	15	5,82E+02	1616,8523
EVAGAVGKGTPVVLM(+15,99)	15	5,80E+02	1442,7803
PTVNATLVGATFPR	14	5,80E+02	1442,7881
YPHM(+15,99)HC(+57,02)Q	7	5,80E+02	987,3691
YAYVLVHQP	9	5,79E+02	1088,5654
LTLWM(+15,99)DC(+57,02)DSVPLA	13	5,79E+02	1535,7
VM(+15,99)NGM(+15,99)HSTGE	10	5,77E+02	1093,4167
PMLRLY	6	5,77E+02	791,4363

SGGGGALEHSANPS	14	5,77E+02	1239,5479
YDLSRTPLL	9	5,76E+02	1076,5867
NSAHTGWAAVSPY	13	5,76E+02	1359,6208
WLETRHGAEPC(+57,02)DAGYYL	17	5,75E+02	2036,905
SSAVLGVTSNVHL	13	5,75E+02	1282,688
YTLHQLFPEGC(+57,02)FVQTM(+15,99)	16	5,74E+02	1985,9016
DVSLPPNNLEFGYDPM	16	5,73E+02	1806,8135
VLADVLPSPAPL	12	5,72E+02	1190,6909
VAPETM(+15,99)VM(+15,99)DGQ	11	5,72E+02	1208,5054
EAVMGALALC(+57,02)NVQ	13	5,70E+02	1374,6636
DVFVPEPKSLH	11	5,69E+02	1266,6609
AHPVSLLVGPN	11	5,67E+02	1102,6135
VVFDDTSWKTNKSR	14	5,66E+02	1681,8423
DQNHVMSTFLAF	12	5,66E+02	1408,6445
LPTSPTLGHTPPP	13	5,64E+02	1313,698
YVNGPKLLRY	10	5,62E+02	1221,687
VNSSVGSYDYVAL	13	5,61E+02	1372,6511
QGPLEAKQGGAGSPPL	16	5,60E+02	1505,7837
MLRAASVVR	9	5,59E+02	1001,5804
MDAGLN(+,98)ANLGDGSLRPY	17	5,58E+02	1763,8147
FPQDEGNLGDGSLRM(+15,99)L	16	5,58E+02	1763,8147
MLVSELTEVNALP	13	5,57E+02	1414,7378
AGYN(+,98)FPLGLLPK	12	5,56E+02	1289,7019
RSPAGMLGSLAR	12	5,53E+02	1214,6553
TKAALVADVF	10	5,52E+02	1033,5808
RTFGNNPGSVSKVP	14	5,52E+02	1458,7578
NEAPQ(+,98)LVPAHADFL	14	5,52E+02	1521,7463
KGSVPEAGALL	11	5,50E+02	1040,5865
LYADSPAHNGTLF	13	5,50E+02	1404,6672
TM(+15,99)HDPLVSAAAM(+15,99)TLYL	16	5,46E+02	1764,8425
VGGERKPVTK	10	5,46E+02	1069,6243
NGGVPLKPGM(+15,99)DLLQ	14	5,45E+02	1453,7598
C(+57,02)VLVGALVSAPELPLKMP	18	5,44E+02	1893,0466
EGSKSPFAAN	10	5,42E+02	1006,4719
LTTN(+,98)QGKSGFVK	12	5,41E+02	1279,6772
PGAPNVEYL	9	5,41E+02	958,476
WGMLDANLALVTSF	14	5,40E+02	1536,7646
PSEPDWMGH	9	5,38E+02	1054,4177
AVPETSYTAVAVK	13	5,37E+02	1334,7083
VM(+15,99)TNHM(+15,99)GSE	9	5,35E+02	1036,3954
NPSPM(+15,99)SSDVKDE	12	5,35E+02	1320,5503
MFVFVKPHGLP	11	5,35E+02	1270,6897
QDPFDANLY	9	5,35E+02	1081,4717
NVEESN(+,98)AAQLTLL	13	5,35E+02	1401,6987
LN(+,98)LGLLLERLL	11	5,34E+02	1266,791
ERSPGHQPQ	9	5,34E+02	1034,4893
NVMFGDGLN	9	5,34E+02	965,4277
FNTC(+57,02)LVGGRVFL	12	5,32E+02	1381,7175
WDM(+15,99)KQSLLV	9	5,31E+02	1134,5742

KVVDGGASRNVNGGGPK	17	5,29E+02	1610,8489
NVGTGLFLGATSLEP	15	5,27E+02	1474,7666
VGNDVKVLPH	10	5,26E+02	1076,5979
VTN(+,98)GTNANRYLY	12	5,25E+02	1385,6575
HLVGDPKLKR	10	5,24E+02	1161,6982
DAALAGFVVHV	11	5,21E+02	1097,5869
MGMAQESATNGE	12	5,21E+02	1224,4751
QKLPTGFFKLAAP	13	5,21E+02	1416,8127
LLAPAGFVFVE	11	5,21E+02	1161,6433
VAVSGLPVFSF	11	5,20E+02	1121,6121
NALTVFKVVSKVP	13	5,19E+02	1400,8391
KSTTSGEVLGYGDPR	15	5,19E+02	1565,7686
QFLAFGLQVM(+15,99)GSPR	14	5,19E+02	1565,8025
LLMAAKSASLL	11	5,19E+02	1116,6577
LTVGSASPLNP	11	5,18E+02	1054,5659
RLTAVPETR	9	5,14E+02	1041,593
LNGLLPHSAAALN	13	5,14E+02	1289,7092
SDAAVDGALL	10	5,13E+02	930,4658
YDDVSNGRAVTQN	13	5,12E+02	1437,6484
VMTQPAAGSRVMHLL	15	5,05E+02	1609,8433
MQNTAKAASGPD	12	5,05E+02	1189,5398
WALPTAVEFVHVD	13	5,05E+02	1482,7507
WVLALDVTADKF	12	5,05E+02	1376,7339
LLASAWPMLGAL	12	5,04E+02	1241,6841
AGELKVANQYVK	12	5,04E+02	1318,7244
EPTPEGSSSGYG	12	5,03E+02	1166,4727
LQNPPSMVL	9	5,02E+02	997,5267
MLKAFMSMTALP	12	5,02E+02	1339,6702
VKSVPEVGRL	10	5,01E+02	1082,6448
KATVMGKNPLVSPK	14	5,01E+02	1468,8435
LTGKKGSM(+15,99)VKLL	12	5,00E+02	1289,7742
MVGSAFAPAYAHA	13	4,99E+02	1291,6018
SSVLTGHPPLLVK	13	4,96E+02	1346,7922
MVVVHVLSA	9	4,95E+02	953,5368
RAKLKFGR	8	4,94E+02	974,6137
M(+15,99)VANNPLFPGLM(+15,99)	12	4,94E+02	1334,6362
HSELQPC(+57,02)LHSK	11	4,94E+02	1334,6401
VSESAAPSAPY	11	4,92E+02	1077,4978
VVN(+,98)ENGLFGAAHHGADVV	18	4,92E+02	1805,8696
NTGSTVQAKKPLN	13	4,91E+02	1356,7361
KDNVLAAPGAYAAQVL	16	4,91E+02	1599,8621
NANAPGM(+15,99)HVGLTTE	14	4,88E+02	1426,6511
YLTSESC(+57,02)GNNAYP	13	4,87E+02	1474,6035
VMPVVMEVDGMV	12	4,86E+02	1304,6179
ANQNNQLDQPAAGPQLA	17	4,86E+02	1748,8442
HPTLGSSFYYGKGLLGGVEAAAQ	23	4,86E+02	2322,1643
EHEDMKFLNNDALP	14	4,80E+02	1671,7563
NNSALPGLLQ(+,98)AM(+15,99)M(+15,99)GC(+57,02)P	16	4,79E+02	1705,7473
NMAVLLVGTAP	11	4,79E+02	1084,595

LAPHGPKLL	9	4,78E+02	944,5807
M(+15,99)GALGGLGPSPFS	13	4,77E+02	1205,575
DDVLTPLSKAQDLPK	15	4,77E+02	1638,8828
NVKEEGSKADAAALMSVEGSLVP	23	4,77E+02	2301,1521
QDGALPPSQNANPVK	15	4,77E+02	1534,7739
HC(+57,02)GEGEFFLNP	11	4,76E+02	1305,5447
PKKFPPTPGVVVM(+15,99)L	14	4,74E+02	1524,8738
PLLKADEVTPK	11	4,74E+02	1209,6968
FFYGESGN(+,98)FVSTYGPQY	17	4,73E+02	1962,8311
NVEGPVLTSGGM(+15,99)	12	4,73E+02	1175,5493
VVN(+,98)ENGLSTTGAKASMAGTGLAP	23	4,67E+02	2146,0576
MLLLTPHNDSPP	12	4,66E+02	1333,6699
YTHLLSGVVE	10	4,66E+02	1116,5815
MKSHKAHFVVANAKME	16	4,65E+02	1826,9285
VPLDGKPPL	9	4,63E+02	934,5487
NSRLVPLSSP	10	4,60E+02	1068,5928
YPGNYQPY	8	4,58E+02	1000,429
GTKVHRVPGGGL	12	4,57E+02	1176,6726
FMWGHYPGGRTTPM	14	4,57E+02	1636,7278
PGVVLLLH	8	4,56E+02	846,5327
RGNLLWLMWME	11	4,55E+02	1447,7104
EPNPLGQQFLAAF	13	4,53E+02	1430,7192
PYHGKDNKKWLDQ	13	4,52E+02	1627,8105
DFVVVGPVVPKPTR	14	4,51E+02	1508,8713
PSSM(+15,99)DAR	7	4,50E+02	778,3279
ALLWPAKP	8	4,47E+02	894,5327
VSLEPDSSVSH	11	4,46E+02	1155,5408
VGGPGLDAYKL	11	4,46E+02	1088,5867
GPGQEFRAN	9	4,44E+02	974,457
MVQVAGLVANYAPL	14	4,44E+02	1444,7749
LFGM(+15,99)KMASEGGDQW	14	4,44E+02	1571,6748
QAAN(+,98)LVTPKLL	11	4,43E+02	1167,6863
MSLVLGSPALFDNFFVLPPM	20	4,42E+02	2194,1206
SSAGDENGPGDEQ	13	4,40E+02	1261,4695
ALAAAFGLKK	10	4,39E+02	988,6069
KVAPAYVK	8	4,38E+02	874,5276
EPAASVSDGNPAMSFPEFL	19	4,38E+02	1964,8826
RGFSWRTS	8	4,38E+02	995,4937
FSNTTASSS	9	4,38E+02	900,3825
AAFVFMGHGRP	11	4,38E+02	1188,5862
MFKPANSSFLLLKNRKPDSLMF	22	4,37E+02	2583,3704
AAALLPHVHN	10	4,36E+02	1041,572
M(+15,99)GNFVGVAPGSNRSQ	15	4,34E+02	1535,7151
LWANGKEM(+15,99)GQPFPR	14	4,32E+02	1645,8035
M(+15,99)APVQTAGAH	10	4,32E+02	997,4651
GMVSLEGNLGQQGSFVK	17	4,30E+02	1749,8718
M(+15,99)VLVALMEYMPNTF	14	4,29E+02	1673,7866
ADLEPDGGGPTK	12	4,29E+02	1155,5408
QGSVKGC(+57,02)KLYLQ	12	4,28E+02	1379,7231

ATVPFNKAAGVL	12	4,25E+02	1186,6709
PEEVVAHAF	9	4,23E+02	997,4869
EGNTEFGGLLV	11	4,22E+02	1134,5557
PLTTTQP	7	4,21E+02	756,4018
C(+57,02)AKAGEGNLLVKDL	14	4,21E+02	1486,7812
LGGVNNVVPP	10	4,18E+02	964,5342
VLSWVAYK	8	4,18E+02	964,5381
NGN(+,98)SLGSGVSNPGK	14	4,18E+02	1287,6055
PESLSASLLQL	11	4,16E+02	1156,634
TALPDATDAH	10	4,13E+02	1010,4669
AWLPTALEC(+57,02)GKHLQ	14	4,13E+02	1622,824
LFLVFTSVRP	10	4,11E+02	1177,6858
VQAPLLVGPN	10	4,10E+02	1006,5811
EKNAGKATAAGDLGVNP	17	4,08E+02	1611,8215
PDATKGSEPV	10	4,08E+02	999,4872
GGTFLAFAGKHL	12	4,07E+02	1217,6558
LTAGVPLLEEVGVPPE	16	4,07E+02	1618,8818
KTPVFPPTQ	9	4,05E+02	1013,5546
LAVGVAALVLGH	12	4,02E+02	1118,6812
RN(+,98)LPGC(+57,02)M(+15,99)M(+15,99)AETF	12	4,00E+02	1458,5942
MPAKSTADSLLNGE	14	4,00E+02	1432,6868
SVALPTDLK	9	3,98E+02	942,5386
FEGESGVVTFPM(+15,99)D	13	3,98E+02	1429,6072
MLKVTSKAKA	10	3,97E+02	1075,6423
LMKVGFLRL	9	3,97E+02	1075,6575
LTLGPVLSSAHS	12	3,97E+02	1180,6453
ASSGVGALLFPGVDA	15	3,96E+02	1359,7034
YELVVALGAHLM(+15,99)	12	3,95E+02	1330,6956
M(+15,99)ALLGVEP	8	3,95E+02	844,4364
PTMASADNLGEYLY	14	3,95E+02	1543,6865
MDAGGLSSM(+15,99)EDDRA	14	3,94E+02	1469,5762
GAVLNKSVKVE	11	3,94E+02	1142,6658
VLGAKYK	7	3,93E+02	777,4749
WNAVLAVC(+57,02)APLL	12	3,91E+02	1325,7166
VLTGDAALLPPK	12	3,91E+02	1193,7019
VGPAKTTPH	9	3,91E+02	906,4923
VFVPPGRML	9	3,91E+02	1014,5684
VFVPQVAPGGAH	12	3,90E+02	1177,6243
FMLSKPPL	8	3,90E+02	931,5201
GSQLANGNSVFTANVE	16	3,89E+02	1606,7588
LAHAMLVVTGLMK	13	3,89E+02	1382,7778
QTGVLRKSSK	10	3,88E+02	1102,6458
C(+57,02)ASKAQVWNLN(+,98)A	12	3,87E+02	1361,6396
SAAGGAADVSVPA	13	3,86E+02	1071,5195
FEAGNLLDEFQKM(+15,99)	13	3,85E+02	1556,718
NNGEKPLVL	9	3,85E+02	982,5447
DDSLPAGTRS	10	3,83E+02	1017,4727
VDLTTYM(+15,99)QGPDFDEAETF	18	3,83E+02	2093,8774
LMAGPDRQHPR	11	3,80E+02	1276,6458

LAPDALGHANL	11	3,78E+02	1090,5771
NPDKVVLDVKESDAARGE	18	3,77E+02	1940,9802
FEKEKEMTSGK	11	3,77E+02	1312,6333
LVVN(+,98)GDC(+57,02)GSTPLA	13	3,74E+02	1302,6125
FARGNPLFAGLL	12	3,72E+02	1274,7134
MNVSLSDGYPADL	13	3,72E+02	1380,623
SLTPLGPAGPVPGPK	15	3,72E+02	1386,7871
HPLPQAATAM(+15,99)	10	3,67E+02	1051,512
KTELLTSS	8	3,66E+02	877,4756
Q(+,98)AHAPPGAE	9	3,64E+02	877,3929
VNGAAEGEPL	10	3,63E+02	955,4611
MSSVSKVPPA	10	3,61E+02	1001,5215
LAEPSGQKPE	10	3,60E+02	1054,5295
LGTPETASVTVL	12	3,59E+02	1186,6445
VPVLLPHSVNNL	12	3,59E+02	1300,7502
MGAGGFLAHSL	11	3,59E+02	1059,5171
Q(+,98)ANKVHGTYGEKHQKRL	17	3,58E+02	1994,0444
Q(+,98)SDAAVNELL	10	3,57E+02	1059,5083
MDDYGFQ(+,98)LVNQLHN	14	3,56E+02	1693,7405
LLASSTGGVLN	11	3,55E+02	1030,5659
DNPGPHGVLN	10	3,55E+02	1018,4832
C(+57,02)ANATKGLAN	10	3,55E+02	1018,4866
GDN(+,98)SLWNLKAVV	12	3,51E+02	1315,6772
ELLMGGLTGPGTQ	13	3,51E+02	1272,6384
NLNDVGDTVFN(+,98)GNVGVKANVN	21	3,51E+02	2160,0447
PTPGAEDDLMVH	12	3,49E+02	1280,5706
MVSVVVGPVP	10	3,47E+02	982,5521
YDWC(+57,02)PGHD	8	3,42E+02	1048,3708
NKKGWPADGSTSLWALLL	18	3,41E+02	1956,0469
MLLALDASPLVTVYTPGPV	19	3,41E+02	1956,0642
M(+15,99)DGAGEEHNV	10	3,40E+02	1073,4084
MFKPSNGNSAAGAL	14	3,38E+02	1363,6555
KPPAYSNSKPLLY	13	3,37E+02	1476,7976
FGLVHAGLK	9	3,36E+02	940,5494
KGAGSFVGKL	10	3,36E+02	962,5549
FLM(+15,99)TFTNMANY	11	3,32E+02	1367,5891
LMKVVTGMVGGGVNRVVK	18	3,31E+02	1843,0535
MVGGTTSPNLE	11	3,30E+02	1104,5122
DSSPNNKMS	9	3,29E+02	978,4077
PVLPVVTPGPGMGLVPPRN	19	3,29E+02	1896,0654
YASMDSSLY	9	3,29E+02	1035,4219
FPGTTGSMSPSPD	13	3,26E+02	1279,5391
MNVHPLVVAKTTWFL	15	3,25E+02	1754,9541
VVPSASRSGV	10	3,25E+02	957,5243
VFVPLTGSPV	10	3,24E+02	1014,575
HSVKNPHSVGLAH	13	3,24E+02	1381,7214
MLAALGNYMHGK	12	3,24E+02	1304,637
MTTGHSAGC(+57,02)LYVW	13	3,24E+02	1481,6431
SADVFLKTPATPE	13	3,23E+02	1374,7031

KAWLSLMANGLLL	13	3,23E+02	1428,8162
HGGLPLRL	8	3,22E+02	861,5184
HNLLPLR	7	3,22E+02	861,5184
DVEKLAAGSF	10	3,22E+02	1035,5237
GNKVSPM(+15,99)GKRGSN	13	3,21E+02	1346,6724
VAQPSSM(+15,99)AVDVQ	12	3,20E+02	1246,5864
QTAVM(+15,99)LLQPQ	10	3,16E+02	1143,5957
LDVFPAGAAPVP	12	3,15E+02	1152,6179
M(+15,99)VDVNAMSSPPPYYL	15	3,15E+02	1698,7634
VQGVPKKGPN	10	3,14E+02	1022,5872
VTVASAAPSSGQVK	14	3,14E+02	1300,6987
FFNETGYVLLLDTSC(+57,02)	15	3,13E+02	1777,8232
RSFATTLEVHV	11	3,13E+02	1258,667
LDAALLGPR	9	3,13E+02	924,5392
PGPVLAGHKP	10	3,12E+02	971,5552
LEFGLFLGDALEGK	14	3,11E+02	1507,7922
PDDVGSGNAAPPGNLVQ	17	3,10E+02	1606,7585
LLYPNFNRTLPL	12	3,10E+02	1459,8186
QALPGAGTFPADKT	14	3,08E+02	1372,6987
HASLATRTTK	10	3,08E+02	1084,5989
ESASKSGGGSPVL	13	3,07E+02	1174,583
YMVAKPGGVLNWLMPQ	16	3,04E+02	1802,9211
PFGGDFLSLAPERA	14	3,04E+02	1475,7407
NGPEDEFVGAAN	12	3,01E+02	1218,5151
RVTPGGVKFLSSF	13	3,01E+02	1393,7717
PGEDARVVF	9	3,00E+02	988,4977
ELGVPVGLDFNC(+57,02)	12	3,00E+02	1318,6228
MFAQGGHVDALAPSYY	16	2,98E+02	1725,782
KTSLTGVWML	10	2,98E+02	1134,6106
SMPEVVHAE	9	2,91E+02	997,4539
QTDMTYSGLVSGLESVATTNGMDVLNM	27	2,90E+02	2833,282
VQSPLVLY	8	2,88E+02	917,5222
LFNMVKL	7	2,87E+02	863,4939
VVNENGNTVFDDLPT	15	2,87E+02	1632,7632
PPSNNNTM	8	2,87E+02	873,3651
C(+57,02)SLAANSVVHELGSHW	16	2,86E+02	1765,8206
VSALPGSALVL	11	2,83E+02	1025,6121
ALSFPPTGPVVVLM(+15,99)	14	2,81E+02	1442,7842
LGGWSPLKDL	10	2,80E+02	1084,5916
SNGNPEGGVGST	12	2,80E+02	1074,4578
MALLHAALAAR	11	2,79E+02	1136,6487
LAASKGPGVPGAL	13	2,79E+02	1136,6553
SVSAGAVPR	9	2,77E+02	842,461
VFLTAFTTKGEL	12	2,74E+02	1325,7231
QLSAQ(+,98)KAVPVGHRA	14	2,73E+02	1461,8052
LRQAQVL	7	2,73E+02	826,5024
LSLPVASLDA	10	2,71E+02	984,5491
LKEAANGAVL	10	2,71E+02	984,5604
KGEAAGGLLLE	11	2,71E+02	1056,5815

TGGGLSSKTPME	12	2,70E+02	1163,5493
RDPLTPANGYLANK	14	2,70E+02	1528,7998
ESVSHSLY	8	2,68E+02	920,424
LYGFLVL	7	2,68E+02	823,4843
FPPLSLYGTL	10	2,66E+02	1106,6011
ESDAARGELL	10	2,66E+02	1059,5195
GTPAQQVLDVQ(+,98)KSVTVN	17	2,65E+02	1783,9316
EGGGGHPTGYFLAN	14	2,64E+02	1375,6157
EGTSLWDGFL	10	2,61E+02	1123,5186
EDKFLQLQGANL	12	2,58E+02	1374,7144
LASPVGFLDHGTGLLE	16	2,58E+02	1624,8459
VPVNLHMTVLF	11	2,56E+02	1268,6951
SSEQLRLVGNP	11	2,54E+02	1198,6306
TPDEVGESVFDGS	13	2,54E+02	1337,5623
QNEAAPGFVFDGS	13	2,54E+02	1337,5886
QGSAQMAAVEGK	12	2,52E+02	1175,5605
KSFVNSAVAPL	11	2,52E+02	1131,6287
TGSWLSNTAADDAPL	15	2,50E+02	1517,6997
VPEGRVVE	8	2,50E+02	883,4763
VFVNVVHA	8	2,50E+02	883,4916
PPSATRGHS	9	2,49E+02	908,4464
PKEGLGFSLPLQ	12	2,47E+02	1284,7078
LHAGGVVNSYAPAL	14	2,45E+02	1367,7197
VLPKKHP	7	2,42E+02	817,5174
LLVNDRWSVFN(+,98)GQ	13	2,41E+02	1547,7732
LHKSAGSNGMT	11	2,41E+02	1101,5237
RNQDPAHSPR	10	2,40E+02	1176,5747
AVPDAFAGPA	10	2,39E+02	914,4497
NLAPQRLVGPN	11	2,38E+02	1177,6567
LQHNVGMQL	9	2,38E+02	1038,5281
VLGVGTANTDLAPPVATY	18	2,35E+02	1757,9199
PGGPGVEAVY	10	2,34E+02	944,4603
NNQGGVFSSLVLM(+15,99)	13	2,34E+02	1380,6707
NNAWVFLKALTPN	13	2,31E+02	1486,7932
VGLKKPH	7	2,31E+02	777,4861
FTKRLVGPN	9	2,29E+02	1030,5923
ARAAMKGVSF	10	2,27E+02	1036,5488
VQGGSPLVL	9	2,25E+02	868,5018
LALLDVHS	8	2,23E+02	866,4861
EVALWPNTSDMM(+15,99)	12	2,21E+02	1408,6003
LELDNYPAGAGPE	13	2,20E+02	1344,6196
VPAGVSVTAH	10	2,19E+02	936,5029
LAVAGVALVLGH	12	2,18E+02	1118,6812
PQGNAAGQETAM(+15,99)F	13	2,18E+02	1336,5718
LSGLPTDFK	9	2,15E+02	976,5229
NVAVGEQHVLN	11	2,15E+02	1178,6042
LGTATGAAKN	10	2,14E+02	902,4821
PKKDAASPNSPKLLY	15	2,14E+02	1627,8933
NPEPNAGDSFPL	12	2,07E+02	1256,5674

LMVNGVLFNL	10	2,07E+02	1118,6157
MYVLMNEGGAPL	12	2,06E+02	1293,6096
LGSDYLGGPA	10	2,05E+02	948,4552
VTTDVFVAAVALEP	14	2,03E+02	1430,7656
VGPFVLLAVSAP	12	2,02E+02	1168,6855
EGSKLGFGLVGL	12	2,01E+02	1175,655
SATPNAAKVGE	11	1,99E+02	1043,5247
PQNAKLVDVE	10	1,97E+02	1111,5874
NGPQGPMARSQ	11	1,92E+02	1141,5298
EM(+15,99)AVALAGAQAPAWLM(+15,99)	16	1,92E+02	1660,7952
RKGYPSGPTGGNP	13	1,91E+02	1286,6367
LLTASGHSL	9	1,91E+02	897,4919
PELGKGQGL	9	1,91E+02	897,4919
MFWQQVLNWGQHM(+15,99)	13	1,90E+02	1719,7651
Q(+,98)SN(+,98)PARC(+57,02)LL	9	1,87E+02	1059,502
Q(+,98)SDAANEVLL	10	1,87E+02	1059,5083
SSKQWYLYGDGSLRM(+15,99)L	16	1,80E+02	1918,9248
NKSLVKL	7	1,76E+02	800,512
PNKDSQP	7	1,68E+02	784,3715
VTFVAPQP	8	1,66E+02	857,4647
M(+15,99)SFWADM(+15,99)	7	1,65E+02	918,3252
VSLLLGPTASPP	12	1,62E+02	1150,6597
TDPPPSLSNPFGYDDL	16	1,57E+02	1733,7783
KNGPSSFA	8	1,56E+02	806,3922
M(+15,99)PSEGDDN(+,98)PGVQ	12	1,56E+02	1261,4768
PEVELGAPL	9	1,55E+02	923,4963
ASAPLNAAPL	10	1,55E+02	923,5076
QSESSNSATE	10	1,53E+02	1038,4102
EGTALTLKM(+15,99)L	10	1,50E+02	1091,5896
YNFVYGMGSGFP	12	1,48E+02	1337,575
MGGEGMC(+57,02)GDV	10	1,44E+02	1011,346
LLAVGHTC(+57,02)HHHLLNL	15	1,42E+02	1733,9148
KSVAGGSVAPAN	12	1,41E+02	1056,5564
LELSGPLPAA	10	1,36E+02	966,5386
ENFVVHYAGPE	11	1,36E+02	1260,5774
LPGKLMGPGGLV	12	1,35E+02	1137,658
VFVPGGAGALK	11	1,34E+02	1014,5862
C(+57,02)SYSVLMYLDFLMFN	15	1,30E+02	1901,8401
VGNALVTPH	9	1,30E+02	906,4923
FVVALPAAFGLAP	13	1,27E+02	1271,7278
TANVPEASATPLA	13	1,21E+02	1240,6299
GKVGVLPHVSDLL	13	1,21E+02	1332,7766
QVVAMMATLK	10	1,09E+02	1090,5879
ETDGLNYPVM(+15,99)P	11	1,07E+02	1250,5488
LGGVLPHTGAALN	13	1,00E+02	1218,6721
VLVFPGTKVE	10	9,96E+01	1087,6277
KGNVMKTSNR	10	9,91E+01	1133,5974
VFPVTSGHA	9	9,56E+01	913,4658
LLLGFPGALDMAAGR	15	9,17E+01	1500,8123

YTAEEM(+15,99)NGG	9	8,96E+01	986,3651
MAPMNVSPSEVK	12	8,60E+01	1288,6155
LWMGLGPFFEGEAPGQ	16	7,56E+01	1734,8076
VQTLFAKN	8	7,45E+01	919,5127
FVPAADALL	9	6,71E+01	915,5065
PEVM(+15,99)KALL	8	6,71E+01	915,5099
RLTR	4	0	544,3445
GHYPK	5	0	600,3019
SWKPV	5	0	615,338
MAAKPV	6	0	615,3414
AGLGNFA	7	0	648,3231
LHASKN	6	0	668,3605
LHFRP	5	0	668,3758
LHRPF	5	0	668,3758
LFPRH	5	0	668,3758
LHRPF	5	0	668,3758
LHRLM	5	0	668,3792
LHRML	5	0	668,3792
PLDVNL	6	0	669,3697
PLDKTP	6	0	669,3697
PEVLGR	6	0	669,3809
RENPGV	6	0	670,3398
TSPNPR	6	0	670,3398
DTKTAH	6	0	671,3239
RESER	5	0	675,33
TNVFAK	6	0	678,3701
HQKLR	5	0	680,4081
NTVGFGP	7	0	690,3337
EEPC(+57,02)C(+57,02)	5	0	693,2098
M(+15,99)LVGPY	6	0	694,336
STLHNQ	6	0	698,3348
GAGKGAPF	8	0	703,3653
VVHAKY	6	0	715,4017
LLHLPQ	6	0	719,433
ADLAGGMS	8	0	720,3112
VLKKHP	6	0	720,4646
KRSGHAA	7	0	725,3932
DVPPSVL	7	0	725,3959
NFLVLK	6	0	732,4534
LLFLGSS	7	0	735,4167
EGTLGFD	7	0	737,3232
VGPVVGLV	8	0	738,4639
PLLGLRA	7	0	738,4752
AGDGNC(+57,02)M(+15,99)	7	0	739,2265
PLLERL	6	0	739,4592
PDNRLQ	6	0	741,377
PEVAKAE	7	0	742,386
PEVSKSP	7	0	742,3861
PEVGRVS	7	0	742,3973

PEVAATR	7	0	742,3973
GGAPDSSGP	9	0	743,3085
Q(+,98)PVSVTL	7	0	743,4065
SAC(+57,02)M(+15,99)TC(+57,02)	6	0	744,2241
PDWAKE	6	0	744,3442
PDAVTGW	7	0	744,3442
PN(+,98)ADLSK	7	0	744,3654
PDATSAAL	8	0	744,3654
TGVKKVN	7	0	744,4494
TTQGGGAR	8	0	746,3671
RGSSGAGR	8	0	746,3783
VVVTAMK	7	0	746,436
VLPPGHK	7	0	746,4439
NRAFNQ	6	0	748,3616
VGLVLLH	7	0	749,4799
SSPLSSSS	8	0	750,3395
SSPHPVK	7	0	750,4024
Q(+,98)GRYVK	6	0	750,4024
LMFNVK	6	0	750,4099
QC(+57,02)PGHGP	7	0	751,3071
FVPYVK	6	0	751,4268
RAGAGPKP	8	0	752,4293
LAAKGSAH	8	0	753,4133
PAPFNLP	7	0	754,4013
EVGDVHT	7	0	755,345
M(+15,99)GGPEDH	7	0	757,2701
M(+15,99)SFDDM	6	0	760,2408
GSFVKLL	7	0	762,4639
DGPANYK	7	0	763,35
PGLTKGPP	8	0	765,4385
PLTGKGPP	8	0	765,4385
KLVGVGGH	8	0	765,4497
PLETNPP	7	0	766,3861
PLETPNP	7	0	766,3861
LAQLPLL	7	0	766,4952
NGGAEM(+15,99)SS	8	0	767,2756
QSSM(+15,99)GDE	7	0	768,2596
TSSSSEGD	8	0	768,2773
VLPDTKP	7	0	768,4381
VPLVADGV	8	0	768,4381
VPEVNLV	7	0	768,4381
VPEVVVQ	7	0	768,4381
VPEVPTK	7	0	768,4381
VPELPKS	7	0	768,4381
VPELPSK	7	0	768,4381
VPELLAQ	7	0	768,4381
VPEVLNV	7	0	768,4381
VPELPSK	7	0	768,4381
VPEVVNL	7	0	768,4381

VPETKVP	7	0	768,4381
VPLDRVA	7	0	768,4493
VPEVLGR	7	0	768,4493
VPLVDAR	7	0	768,4493
YVLKSY	6	0	771,4167
LKPGNML	7	0	771,4313
SKAPLTR	7	0	771,4603
RTALALQ	7	0	771,4603
VGNLLTR	7	0	771,4603
RGGLLTR	7	0	771,4715
RLGGLTR	7	0	771,4715
RGAVLTR	7	0	771,4715
RAGVLTR	7	0	771,4715
RLGGLTR	7	0	771,4715
GVARLTR	7	0	771,4715
RGGKKAR	7	0	771,4827
TAVPEGVT	8	0	772,3967
TAVPEAVS	8	0	772,3967
TAVPETR	7	0	772,4079
TAVQAGNL	8	0	772,4079
TAVPETR	7	0	772,4079
TAVPSGGKG	9	0	772,4079
TAVPERT	7	0	772,4079
TAVSGGGKP	9	0	772,4079
TAVPSNGK	8	0	772,4079
TAVRGGVN	8	0	772,4191
EPSAEM(+15,99)P	7	0	775,3058
DDGRMAN	7	0	777,3076
GVLKKPH	7	0	777,4861
GVLKHKP	7	0	777,4861
PEPVTHT	7	0	779,3813
M(+15,99)VTTDSL	7	0	781,3528
PEVLTKP	7	0	782,4538
PEVGRLL	7	0	782,465
NSSAVGDH	8	0	785,3304
NPPAMEE	7	0	786,3218
VPFVVKV	7	0	786,5003
SSYAFTN	7	0	788,334
LKPNSTM	7	0	789,4055
LKPSNTM	7	0	789,4055
SC(+57,02)KKLR	6	0	790,4483
SC(+57,02)KKRL	6	0	790,4483
SGEGDDN(+,98)V	8	0	792,2773
EGLDMTK	7	0	792,3687
EGLHSPGP	8	0	792,3766
NGGLGVYN	8	0	792,3766
VGPVVPMP	8	0	794,436
VLGVGVPGV	9	0	795,4854
PEVKGATP	8	0	797,4283

PEVGVLGQ	8	0	797,4283
PEVQTPK	7	0	797,4283
PEVGRLQ	7	0	797,4395
RPNGSLR	7	0	798,446
TPVAKKR	7	0	798,5075
PN(+,98)ATKRL	7	0	799,4552
FPGHGSLS	8	0	800,3817
KGAAKGGGR	9	0	800,4616
GVLYAGQP	8	0	803,4177
NGPKLLY	7	0	803,4541
DGPKLLY	7	0	804,4381
PFLSFTP	7	0	807,4167
PM(+15,99)LVYGL	7	0	807,42
PSSASDM(+15,99)T	8	0	810,3066
RGPSEEH	7	0	810,362
LPTVQTGV	8	0	813,4596
SAVSPNLE	8	0	815,4025
N(+,98)PVDSRE	7	0	816,3613
QKWEVK	6	0	816,4493
NGC(+57,02)EC(+57,02)GE	7	0	824,2429
RSASPLPV	8	0	825,4708
AGADKAAPQ	9	0	827,4137
VGSVTSAAH	9	0	827,4137
VTPNNRQ	7	0	827,425
SWKVPVL	7	0	827,4905
LLSGEWQ	7	0	831,4127
LLGGDVMQ	8	0	831,416
GAGKVGSEK	9	0	831,445
KGAGVSSVQ	9	0	831,445
KVFSHSK	7	0	831,4603
NLGGTTASL	9	0	832,429
PGPPTQTH	8	0	833,4031
PDLVFPF	7	0	833,4323
VVGAPSPHA	9	0	833,4395
NSPKLLY	7	0	833,4647
VLTYARL	7	0	834,4963
LEAGAGYR	8	0	835,4188
LLAPGATPP	9	0	835,4803
SGGGGASM(+15,99)TP	10	0	836,3334
M(+15,99)AGPFGNQ	8	0	836,3487
LAVVPMPL	8	0	838,4986
LLSGANGPL	9	0	840,4705
PGMVSGVVP	9	0	841,4368
PN(+,98)ATKVVL	8	0	841,4909
MKVLQPK	7	0	842,5048
ASAGGPSARA	10	0	843,4199
N(+,98)AGELNLL	8	0	843,4338
DGSAPATLL	9	0	843,4338
DALNGELL	8	0	843,4338

SVGSGPDLL	9	0	843,4338
VSGSGPDLL	9	0	843,4338
SSAKGNAPL	9	0	843,445
ASTAGAPAAK	10	0	843,445
DAAERGLL	8	0	843,445
VAGQASAQL	9	0	843,445
MFGGGDM(+15,99)D	8	0	844,2731
M(+15,99)ALGPNLN	8	0	844,4113
M(+15,99)ALGPRAN	8	0	844,4225
M(+15,99)AGAPRLN	8	0	844,4225
FALLHMN	7	0	844,4265
FALSPVDP	8	0	844,433
MLSVVPAE	8	0	844,4364
MSLLGPVE	8	0	844,4364
MSLLVGPE	8	0	844,4364
FALLNGPN	8	0	844,4443
M(+15,99)DDM(+15,99)VGM(+15,99)	7	0	845,2606
HGKSPANH	8	0	846,4096
ELLHPLQ	7	0	848,4756
LELHLPQ	7	0	848,4756
LAKGVAGPH	9	0	848,4868
RGVFPM(+15,99)K	7	0	849,4531
LSGSNLLF	8	0	849,4596
AKLELGGY	8	0	849,4596
RGGLTAYN	8	0	850,4297
LAGM(+15,99)AFGGN	9	0	852,3799
RTLMVHV	7	0	854,4797
AASRFPHA	8	0	855,4351
PEVGRTTP	8	0	855,445
PEVWGLR	7	0	855,4603
LPEVGQTL	8	0	855,4702
KLDPTALV	8	0	855,5065
LSPVWMK	7	0	859,4626
VVPGVPVPP	9	0	859,5167
LVVVQGFV	8	0	859,5167
HGPSAAKGH	9	0	860,4253
QNFLVLK	7	0	860,512
KVFTPAAK	8	0	860,512
KPAGVYVK	8	0	860,512
DGGSLVGYP	9	0	863,4025
GDGSLVGM(+15,99)L	9	0	863,4059
N(+,98)GGSLRM(+15,99)L	8	0	863,4171
YPGLRME	7	0	864,4163
VMSLRME	7	0	864,4197
PGVVRPGSP	9	0	864,4817
PELGEPVK	8	0	867,4702
KPERPKN	7	0	867,4926
KDKRAPGP	8	0	867,4926
KDKHVNK	7	0	867,4926

VGAGGGADGLP	11	0	869,4243
EPSAPTLR	8	0	869,4606
EPDNRLQ	7	0	870,4195
GVSRSPNR	8	0	871,4624
LEDRVLQ	7	0	871,4763
LEDARLGV	8	0	871,4763
VNSLNPTK	8	0	871,4763
VLLGDEAR	8	0	871,4763
LVSGPMGVL	9	0	871,4837
LVGLM(+15,99)QPV	8	0	871,4837
EGNGVRLK	8	0	871,4875
MKLAAGGPK	9	0	871,4949
PETKSVVL	8	0	871,5015
NSDGASYC(+57,02)	8	0	872,2971
PN(+,98)ATSKSAP	9	0	872,424
NKLKVSSV	8	0	873,5283
PGPLKLLH	8	0	873,5436
DSLGQAATL	9	0	874,4396
VGHLLEPN	8	0	877,4658
VLHGLEPN	8	0	877,4658
LRHLEPN	7	0	877,477
RTTLLTSS	8	0	877,4869
VPEVNLVL	8	0	881,5222
VPEVLLGR	8	0	881,5334
NHSSYLY	7	0	882,3871
VPSVRVMP	8	0	883,4949
LMM(+15,99)YVVN	7	0	884,4136
PPGGSLVPY	9	0	885,4596
LLKPGDML	8	0	885,4993
NLAPRGFL	8	0	886,5024
PFTPSGSVP	9	0	887,4388
KPRC(+57,02)MTP	7	0	888,431
MLKKMKL	7	0	890,5446
MLKKKLM	7	0	890,5446
LEEYLLN	7	0	892,4542
NQGAGPGFM(+15,99)	9	0	893,3701
GGM(+15,99)FAGQNP	9	0	893,3701
TPPGVPVVE	9	0	893,4858
PTPGVVPVE	9	0	893,4858
VVPVGPAQQ	9	0	893,497
FVVVFGQV	8	0	893,501
VVPGVPVMP	9	0	893,5045
QAPRVPVE	8	0	894,4922
QAPRPVVE	8	0	894,4922
NLPLKTLP	8	0	894,5538
PGAAVVDTAP	10	0	896,4603
VPEVGGLVQ	9	0	896,4967
PEAVGAELL	9	0	897,4807
NMAFGC(+57,02)AE	8	0	898,3313

LTVVFPSH	8	0	898,4912
LDDDGGHGD	9	0	899,3257
LDM(+15,99)DYMP	7	0	899,3405
DDSSKSDF	8	0	899,3508
VFPTQPPD	8	0	899,4388
VFAFSKDS	8	0	899,4388
PPTC(+57,02)AKVK	8	0	899,4899
PYGLLPLK	8	0	899,548
PYGLPLKL	8	0	899,548
SPSASSHND	9	0	900,3573
VMSHRHH	7	0	902,4293
DSLWNRL	7	0	902,461
LLKPSNTM	8	0	902,4896
LLKPSNMT	8	0	902,4896
VRLYAGPQ	8	0	902,4974
NVPKAVLY	8	0	902,5225
AAAVAPKLY	9	0	902,5225
VKAPLNVY	8	0	902,5225
VVNVPTLY	8	0	903,5065
KVFAGGAAGK	10	0	904,513
PSLPGHLW	8	0	905,4759
PEPKPELP	8	0	905,4858
M(+15,99)DTYQSM(+15,99)	7	0	906,3099
VGALGKEPH	9	0	906,4922
AAAAVAVTPH	10	0	906,4923
KAPGTTVPH	9	0	906,4923
TQDC(+57,02)EEE	7	0	909,3022
RGTPLVLVG	9	0	910,56
FKVAVHVL	8	0	911,5593
GANGRGGDPL	10	0	912,4413
AGNPKGC(+57,02)PL	9	0	912,4487
EPEVSKPE	8	0	913,4392
PPEFVVLN	8	0	913,4909
ATSVFVKY	8	0	913,4909
LGPDAFAQP	9	0	914,4498
LGPN(+,98)AFAQP	9	0	914,4498
LGPDADSKL	9	0	914,4709
AVPDATASLA	10	0	914,4709
AVPDALSDK	9	0	914,4709
EM(+15,99)EMTME	7	0	915,3024
MNDYEMN	7	0	915,3102
NGPN(+,98)AGDRD	9	0	915,3682
NGPN(+,98)AKAML	9	0	915,4484
SSAPKADLAG	10	0	915,4661
PVDVLGLGF	9	0	915,5065
LVEMLAGAL	9	0	915,5099
PEVVM(+15,99)VVK	8	0	915,5099
PEGANFER	8	0	918,4195
SNSPKLLY	8	0	920,4967

PFATTGTVK	9	0	920,4967
AGVVFMAVK	9	0	920,5153
LAPAGSPGPR	10	0	921,5032
PSPLAPVLE	9	0	921,5171
PKSLTALPP	9	0	922,5487
HDAGQKLVG	9	0	923,4825
VDLRAALAP	9	0	924,5392
EGLALLPGR	9	0	924,5392
LSVLFLPH	8	0	924,5433
PMPLATALL	9	0	925,5306
PEEVVAHF	8	0	926,4498
LAPDSNKAN	9	0	928,4614
PSPDARTSV	9	0	928,4614
PSVPYGRGP	9	0	928,4766
PSLGTVKDL	9	0	928,5229
MAAGNYGTM(+15,99)	9	0	930,3575
PFKVKLTV	8	0	930,5902
VPEFNKAK	8	0	931,5127
M(+15,99)SVPGEDAL	9	0	933,4113
FEGPSGNVQ	9	0	933,4192
M(+15,99)WVADVTP	8	0	933,4266
M(+15,99)VSPYPQP	8	0	933,4266
PDAAAKKAY	9	0	933,4919
GGLHVLRPS	9	0	934,5349
AGPEAPSGPGP	11	0	935,4348
LN(+,98)MVKMSL	8	0	935,482
HTAASHWK	8	0	936,4566
LGEGPM(+15,99)STM	9	0	937,3885
EQHQKAVV	8	0	937,4981
LGSKGPAAGAL	11	0	940,5341
NGDYMGTAN	9	0	941,3549
PTAGSTGRVP	10	0	941,493
LC(+57,02)GAKLLAP	9	0	941,5368
VSSGALAPLK	10	0	941,5546
PTMGVDKVP	9	0	942,4844
MEGAC(+57,02)TEM(+15,99)	8	0	943,3085
ATGVWGAVW	9	0	945,4708
LLNKMSLK	8	0	945,5681
VSTVTKAKL	9	0	945,5859
YPYMNME	7	0	946,3565
VVSLM(+15,99)GAALA	10	0	946,5157
VKGTPVYLA	9	0	946,5488
LVRNTGFAA	9	0	947,5189
LVGVVPPGNP	10	0	947,544
EYC(+57,02)FSDE	7	0	948,3171
LMM(+15,99)RPASQ	8	0	948,4521
SGSAASSTALV	11	0	949,4716
MVHHTLNV	8	0	949,4804
KGGLMEGPY	9	0	950,4531

ELLMWPY	7	0	950,4572
LELMWPY	7	0	950,4572
LAGVLGVPLL	10	0	950,6164
SKAGKLWY	8	0	951,5178
LLGSLAKGPP	10	0	951,5753
EN(+,98)GEGC(+57,02)TGE	9	0	952,308
QSAGVSPGPGP	11	0	952,4614
AGSAPKPPTQ	10	0	952,4977
MLVFVTLM	8	0	952,5126
VRVLM(+15,99)HAL	8	0	953,548
LGLAPGALDK	10	0	953,5545
AALVPLQKD	9	0	953,5546
KRLVGPDGL	9	0	953,5658
VPEGRLDVA	9	0	954,5134
VPEVRGLGE	9	0	954,5134
VPEVGGLVW	9	0	954,5175
AQGANVTTVP	10	0	956,4927
GNTVSRVPE	9	0	957,4879
RNKPGHHL	8	0	957,5256
PQDFLVLK	8	0	958,5487
M(+15,99)LVLNLLQ	8	0	958,5521
M(+15,99)LVLQVLQ	8	0	958,5521
SATVLKVLE	9	0	958,5699
LFVEPPMK	8	0	959,515
SSVSGALVLK	10	0	959,5651
AVFETPAAR	9	0	960,5029
LVSFRGPGE	9	0	960,5029
PHRPVEDL	8	0	961,4981
M(+15,99)FDAEAQH	8	0	963,3756
NAASAFMRP	9	0	963,4596
LLELLHNL	8	0	963,5753
NLELLKHV	8	0	964,5706
VLSSLVVSY	9	0	965,5433
MVVHGQPAE	9	0	966,4593
MGLTQGTFL	9	0	966,4844
VGPTPPFVGP	10	0	966,5174
VTPPGPFGPV	10	0	966,5174
VEPLLEGVL	9	0	967,559
LGSPTAVPDN	10	0	969,4767
PN(+,98)SAPKMLL	9	0	970,5157
LVM(+15,99)GPLPTK	9	0	970,5521
VPFLVVGLK	9	0	970,6215
GDDSYNMDG	9	0	972,313
GYMGSGDDDG	10	0	972,313
STDDMTC(+57,02)SG	9	0	972,3165
MGVMGGM(+15,99)DC(+57,02)	9	0	972,3174
GDDSDYGTSG	10	0	972,3308
GDDSTGGYDS	10	0	972,3308
GDDSNYTDS	9	0	972,3308

GDDSC(+57,02)AFAM	9	0	972,3317
MGTM(+15,99)GSGGEM	10	0	972,3351
GDATC(+57,02)MTM(+15,99)A	9	0	972,3351
MGDSGSSMAM	10	0	972,3351
LM(+15,99)C(+57,02)GC(+57,02)DTT	8	0	972,3351
MLKVKKAR	8	0	972,6266
GTHYPKSW	8	0	974,461
GDTGPYGFY	9	0	975,3974
PSLFAKASR	9	0	975,5501
PSEGYSDVQ	9	0	980,4087
SAVSLTHGVL	10	0	982,5447
TKNPTGGPVL	10	0	982,5447
NNGTRPLVL	9	0	982,556
TLGAWAVGVL	10	0	985,5596
GSDAATPSGLL	11	0	987,4872
GSDAAGVEGLL	11	0	987,4872
GSDAASPASLL	11	0	987,4872
GSDARQELL	9	0	987,4985
GSDAATPRTL	10	0	987,4985
GSDVC(+57,02)PKLL	9	0	987,5059
SGSPAC(+57,02)ALLL	10	0	987,5059
ATSAMAPGGLL	11	0	987,5059
LLGPDEVFV	9	0	987,5276
FDAVLPQSL	9	0	988,5229
DPGVLAPPPK	10	0	989,5545
AVLDLDFEA	9	0	991,4862
AVLDLDLM(+15,99)S	9	0	991,4896
LGEGQVFML	9	0	992,5001
VVPVGPTPVE	10	0	992,5542
PTPVGVPVVE	10	0	992,5542
VVVGPTPPVE	10	0	992,5542
TPPGVVPVVE	10	0	992,5542
VVVPGPVAQQ	10	0	992,5654
VVPGVVPVNN	10	0	992,5655
VVPVGPVVMP	10	0	992,5729
VVVGEPLLAP	10	0	992,5906
NASGSTLAHH	10	0	993,4628
RGVHLEARG	9	0	993,5468
RGVHLEQR	8	0	993,5468
RRHLEQR	7	0	993,558
RPPDRGAVQ	9	0	994,5308
GGAEEVDHAL	10	0	996,4512
MTADM(+15,99)PMAD	9	0	997,3555
MSPENGHRA	9	0	997,4399
M(+15,99)APEYSTAL	9	0	997,4426
SGGNEPTHSL	10	0	997,4465
MSPEVVEAH	9	0	997,4539
M(+15,99)APEEVVAH	9	0	997,4539
FAPEVAFSM	9	0	997,4579

FAPEPMVHA	9	0	997,4691
LN(+,98)SEVVEAH	9	0	997,4716
PAVSPGGNSTN	11	0	999,4621
NMVGGLQGLL	10	0	1000,5375
TGGGKGSNTPK	11	0	1002,5094
DGTKPVYAR	9	0	1005,5243
LDGVNAYVR	9	0	1005,5243
LC(+57,02)KPVYNL	8	0	1005,5317
PMGVLPQAPP	10	0	1005,5317
VEGVGGHPVR	10	0	1005,5355
SWKYGLPR	8	0	1005,5396
PEEVVAHLL	9	0	1005,5494
LDGVLAYQK	9	0	1005,5494
LAGALVPVGPN	11	0	1006,5811
SVLKPPVGPN	10	0	1006,5811
KTGVLPPGPAA	11	0	1006,5811
LPAAGVLVGPN	11	0	1006,5811
TKGVLAAPGPP	11	0	1006,5811
TQGVLPPKPA	10	0	1006,5811
TKGVLPPGPAA	11	0	1006,5811
TKGVLAPAGPP	11	0	1006,5811
QTGVLAYKK	9	0	1006,5811
LQPGLLVGNP	10	0	1006,5811
NPLLALVGPN	10	0	1006,5811
NPLALLVGPN	10	0	1006,5811
TKGVLAAPGPP	11	0	1006,5811
LPAAVGLVGPN	11	0	1006,5811
TQGVLYAKK	9	0	1006,5811
NPLALLVGPN	10	0	1006,5811
TKGVLPPGPAA	11	0	1006,5811
TQGVLAYKK	9	0	1006,5811
KTGVLPPGAAP	11	0	1006,5811
TQGVLAYKK	9	0	1006,5811
SAAGVLAYKK	10	0	1006,5811
LPAAGVLVGPN	11	0	1006,5811
AGLGPLLVGNP	11	0	1006,5811
QTGVLAYKK	9	0	1006,5811
VVPGRLVGPN	10	0	1006,5923
TKGVLHGKAP	10	0	1006,5923
ALAPRLVGNP	10	0	1006,5923
HSLSGTHNR	9	0	1007,4897
VLDLDHEAP	9	0	1007,4924
PKKGVPDGNP	10	0	1007,54
QPKRLVGGGP	10	0	1007,5876
PPTRPVVLM	9	0	1008,579
PLWYLGYV	8	0	1009,5273
NAKGSFVKY	9	0	1012,5341
MAAKPVVGLQ	10	0	1012,5739
SGTVLALAGEP	11	0	1013,5393

VFVPEPVTK	9	0	1014,575
VFVPEVTKP	9	0	1014,575
VFVGGPVLVE	10	0	1014,575
VFVPELVGR	9	0	1014,5862
VFVPNGGVVK	10	0	1014,5862
VFVPEVLGR	9	0	1014,5862
VFVPERLR	8	0	1014,5974
LLGLANSSGAN	11	0	1015,5298
LLGLANDMGL	10	0	1015,5372
DLGSPPVGYN	10	0	1017,4767
KGGSSVDM(+15,99)PQ	10	0	1020,4546
LQKAVVVGGGP	11	0	1023,6077
PPTPRVVLM(+15,99)	9	0	1024,574
ELKVGLVGPN	10	0	1024,5917
LAGNPMPDVL	10	0	1025,5215
QTGLVGVAGKP	11	0	1025,5869
LTLVLPEEL	9	0	1025,6008
LAVSVNGQPGS	11	0	1027,5298
SAGVPKPLFL	10	0	1027,6066
LDM(+15,99)M(+15,99)NDDGS	9	0	1028,3428
LGGALKFGGPN	11	0	1029,5607
DGGTGTNAARL	11	0	1031,4995
KRLKAGGRF	9	0	1031,6353
C(+57,02)KTTGAVLLA	10	0	1032,5637
KRSLPATFN	9	0	1032,5715
DGDSLTQLGE	10	0	1033,4563
LSPQVGAEHP	10	0	1033,5193
KLGLGMPSTM	10	0	1033,53
KYNLARNGV	9	0	1033,5669
LALTQGATFL	10	0	1033,5808
AASAAAGFSADP	12	0	1034,4668
EVTGNALFVS	10	0	1035,5237
QGDGVDGSSES	11	0	1036,3945
MGASSPVPPPV	11	0	1037,5215
NGAGAVLVLPQ	11	0	1037,5869
LAC(+57,02)NVDGQY	9	0	1038,4441
GMDSTDGC(+57,02)TT	10	0	1043,3535
LNGATSSPGQL	11	0	1043,5247
KSAGGASASGPR	12	0	1044,5312
LGVAAKFNPE	10	0	1044,5603
GLKKGDPVSF	10	0	1046,5759
TADSLTGGGGVN	12	0	1047,4832
YVAAPFGPNL	10	0	1047,5388
LMGAAGSAKRS	11	0	1047,5496
MLEATGSLKV	10	0	1047,5635
LGSANLAAGFK	11	0	1047,5713
NPPAAPKGALN	11	0	1048,5664
ANEMPGGVLY	10	0	1049,4851
QAAESPATLY	10	0	1049,5029

TPPPGGYSLY	10	0	1050,5022
VVPESTGSLY	10	0	1050,5232
SVLATTLSVY	10	0	1052,5754
ALEPDAQAGAL	11	0	1054,5295
ALEPDNLGVQ	10	0	1054,5295
DGDSLVLGSGH	11	0	1055,4883
NPKTGSEVPK	10	0	1055,561
SLGLLVSGTPL	11	0	1055,6226
GKAGVSRSPAK	11	0	1056,604
ESDAPARAML	10	0	1059,502
EQQGGQMGLL	10	0	1059,502
Q(+,98)SDAASPASLL	11	0	1059,5083
Q(+,98)SDAGPKMLL	10	0	1059,5271
ESDAPKAVML	10	0	1059,5271
GVSQKTTAPAT	11	0	1059,5559
DPGVLVGKYL	10	0	1059,5964
FPVGTTSTGPV	11	0	1061,5393
ATGGVLRRHV	10	0	1064,6204
KGPKRLVGNP	10	0	1064,6455
LTGSVASVGLY	11	0	1065,5706
KSEVLLTNY	9	0	1065,5706
YVPPTTLLY	9	0	1065,5747
YVPTPTLLY	9	0	1065,5747
VVLMLDTLY	9	0	1065,5781
VVPGTPPFVGP	11	0	1065,5859
YVNGKPLLY	9	0	1065,5859
YVNAPVKLY	9	0	1065,5859
YVGGGKPLLY	10	0	1065,5859
YVNGLLKPY	9	0	1065,5859
YVNGLKPLY	9	0	1065,5859
YVNPGKLLY	9	0	1065,5859
VPDGLSGPLVL	11	0	1065,6069
VPGVSEGPLVL	11	0	1065,6069
YPLALTLNY	9	0	1066,5698
NPALLTLTQP	10	0	1066,6023
YTHGKTQAY	9	0	1067,5037
LVTPHQPNY	9	0	1067,54
VDAFVAPSPPA	11	0	1069,5444
GKGAGAPVM(+15,99)VAP	12	0	1069,5591
AGPLLANSQSN	11	0	1070,5356
DLNTDLRVK	9	0	1072,5876
PPFSGFEFF	9	0	1073,4858
GADGVGEAAKTV	12	0	1073,5352
EVSQLGSTTGP	11	0	1074,5193
MLKGKKMAGL	10	0	1075,6245
GKKVGPHGALL	11	0	1075,6501
GEKGSFVKLL	10	0	1076,623
YAVPGGAVHAH	11	0	1077,5356
MPAASHSPAPN	11	0	1078,4866

AVLDLDHEPA	10	0	1078,5295
VALDLDHEPA	10	0	1078,5295
FFDVPDNAR	9	0	1079,5037
NKDALEDLY	9	0	1079,5134
ATAGLSVADLY	11	0	1079,5498
KARLPYGGGY	10	0	1080,5715
LVSNSHTVNL	10	0	1082,572
KNSPPTLQAK	10	0	1082,6084
YSNALAGLLY	10	0	1083,5601
YSNSPKLLY	9	0	1083,5601
LHSGGLGAPLY	11	0	1083,5713
EHEGPKLLY	9	0	1084,5552
EHGKPKLNY	9	0	1084,5664
QALLLNHNY	9	0	1084,5664
QALTGGVLNGR	11	0	1084,5989
QALLAHKAAY	10	0	1084,6028
GLPEGFKGKGP	11	0	1085,5869
VAPEGSLSLTL	11	0	1085,5969
LGPGVGAKPKY	11	0	1085,6233
LAAASHPRKH	10	0	1086,6047
YDVSSM(+15,99)DEE	9	0	1089,3809
LNATLADSAES	11	0	1090,5142
HKVEPQTPR	9	0	1090,5884
VVFTEATRVA	10	0	1091,5974
DSDSPGGNQAF	11	0	1093,4312
VEGVLHTGTAL	11	0	1095,5923
MANTGPSSVSM(+15,99)	11	0	1096,4529
NGSKLRGNRP	10	0	1097,6055
GVM(+15,99)EHQKPGT	10	0	1098,5127
NVAASATLHSE	11	0	1098,5305
LPVGDTSTTPN	11	0	1100,5349
QGGVMVLGDLL	11	0	1100,5898
LLLGPDEVVF	10	0	1100,6118
KNSETVPKKA	10	0	1100,6189
LTGSPAMAKLL	11	0	1100,6262
LHLELVGAHL	10	0	1100,6343
SGLDTPSLLAE	11	0	1101,5554
VFN(+,98)KLASPGGL	11	0	1102,6023
KRAAGLFGRK	10	0	1102,6724
FN(+,98)GLDYGNGM(+15,99)	10	0	1103,4229
PPTGVLVVPVE	11	0	1105,6382
LVVVGPTPPVE	11	0	1105,6382
LVVVGPVAPQQ	11	0	1105,6494
LPTPRVVPVE	10	0	1105,6494
FVLVVPGTFK	10	0	1105,6536
MGVTFNPDTE	10	0	1109,47
PSVTGGKAPGLQ	12	0	1110,6033
LGKNTNAN(+,98)PAL	11	0	1112,5825
APGAVGLPPFTS	12	0	1112,5867

NLNAGKGATLR	11	0	1113,6255
EVAGLSPTETL	11	0	1115,571
LC(+57,02)SAM(+15,99)GHVNL	10	0	1116,5056
VSGATPAFAPAE	12	0	1116,5452
TPADKPVGPVH	11	0	1116,5928
GDN(+,98)PMMHGME	10	0	1118,3831
VNPMSHPQLP	10	0	1118,5542
NVPVGPPDPDL	11	0	1118,5608
SQDQHQKLH	9	0	1119,542
NTNEHQKLH	9	0	1119,542
YSSAANVKWP	10	0	1121,5505
GFRM(+15,99)TLGPDL	10	0	1121,554
PSYSNAPQLF	10	0	1122,5344
PSYSNAPGALF	11	0	1122,5344
ATSPEEAQPPV	11	0	1124,5349
LDKVPEVGGVL	11	0	1124,644
KDRVSADPPAA	11	0	1125,5779
PNFVPELLAK	10	0	1126,6387
LTAVLGGDLEAA	12	0	1128,6025
AEPYPLQSPE	10	0	1129,5291
QAGLEAN(+,98)GNGKA	12	0	1129,5364
DVFVPEVGGVL	11	0	1129,6018
VDFVLPAAADL	11	0	1129,6018
VDFVPLDTKP	10	0	1129,6018
VDFVPEPKLS	10	0	1129,6018
DVFVPEVGGVL	11	0	1129,6018
DVFVPQVVDL	10	0	1129,6018
TLM(+15,99)VPLPDTK	10	0	1129,6052
DVFVPEVGRL	10	0	1129,613
DVFVLDGPLR	10	0	1129,613
DVFVPQGGALK	11	0	1129,613
LTFVPSNGKAP	11	0	1129,613
DVFVPEVLGR	10	0	1129,613
LTM(+15,99)VLPEVGR	10	0	1129,6165
LTM(+15,99)VPEGLRV	10	0	1129,6165
VPVVLPSC(+57,02)LF	10	0	1129,6206
TVKMLPVM(+15,99)PV	10	0	1129,624
TLFVPNNLGR	10	0	1129,6243
NGSTHSSEESP	11	0	1130,4475
DGN(+,98)SLADKVVL	11	0	1130,5818
GTVFPDLGVAR	11	0	1130,6084
MALFAAGAGLPN	12	0	1131,5747
SKFVPLVDDL	10	0	1131,6174
KGAGDTTASTLL	12	0	1133,5928
EGEFLGNSALV	11	0	1134,5557
EGNDAFASLLV	11	0	1134,5557
SSGMSVATSLVP	12	0	1134,5591
Q(+,98)GNKM(+15,99)DTLLV	10	0	1134,5591
EGNDYGRLLV	10	0	1134,5669

EGNN(+,98)YRVALV	10	0	1134,5669
ESGKQTMAKGV	11	0	1134,5703
PSYSNSPQLF	10	0	1138,5295
VSSTVKVPGPGL	12	0	1139,655
EPM(+15,99)VPVVSDGP	11	0	1141,5325
LHGPQGMGMSK	11	0	1141,5371
LMGAAGPLDMAP	12	0	1142,5464
LGGFVAAAVKNP	12	0	1142,6448
LLPFVC(+57,02)GAGPN	11	0	1143,5747
NFKNLGVPANA	11	0	1143,6035
LLAGMANVEVK	11	0	1143,6321
NGGAKATSNNNV	12	0	1145,5425
ATASAVASPRES	12	0	1145,5676
WSLQTTAGNGL	11	0	1146,5669
RHLNGPSAAPQ	11	0	1146,5894
NSAAGGLVM(+15,99)LAK	12	0	1146,6067
VSPDGKLVSVF	11	0	1146,6284
KSGDTVDATLAA	12	0	1147,572
VKSHHSSSVAL	11	0	1150,6094
LVPMATSTGLY	11	0	1151,5896
PC(+57,02)LPAAAAGQTP	12	0	1152,5598
VSALPSHALFL	11	0	1153,6494
EPLFRSGHTL	10	0	1155,6035
PARESPAMDAN	11	0	1157,5134
RPFSTGMAHR	10	0	1158,5715
RDSGFTLEVH	10	0	1159,5623
ATVSGFTAGNVH	12	0	1159,5623
PGGYLSVLGARA	12	0	1159,635
DTFVFSLSFV	10	0	1160,5754
GELLPTPLPPQ	11	0	1160,644
ELKTQPVPPR	10	0	1163,6663
VVPRTPTPAVE	11	0	1164,6501
TPVGPVVPFVGP	12	0	1164,6543
VVPGVTPPFVGP	12	0	1164,6543
QSAM(+15,99)KRGGSTK	11	0	1165,5874
VSGGGAGALQKSH	13	0	1167,5996
PDKMVVVFVH	10	0	1169,6267
KTDGVAPKGLGQ	12	0	1169,6404
RGKMVATLAPK	11	0	1170,6907
LLEPVFSSLAP	11	0	1171,6489
LLELVTGM(+15,99)LAP	11	0	1171,6523
YTSSTGPC(+57,02)GVF	11	0	1174,4963
GQLSHSHSQVP	11	0	1175,5684
PM(+15,99)LASGVM(+15,99)LNL	11	0	1176,5884
SHHQDEAAPGM	11	0	1178,4775
LYVNPGKLLY	10	0	1178,6699
LDGPAGTVHLGSG	13	0	1179,5884
TGKVPSSLGAAPV	13	0	1182,6609
M(+15,99)APEEVVAADH	11	0	1183,5178

AMLVSGVPGKVQ	12	0	1184,6587
LGVFVLLGAAEP	12	0	1184,6804
MVTDLPVVGRT	11	0	1186,6379
HLVTFKTGSVV	11	0	1186,6709
KENQKKNGSGV	11	0	1187,6257
C(+57,02)ATAAVRVEVL	11	0	1187,6333
MTKNKGPVTVN	11	0	1187,6333
RGLPGVTPRVH	11	0	1187,6887
LM(+15,99)GDGGAYYNN	11	0	1189,4709
QQYGSSQKLR	10	0	1193,6152
QQEQHKLGAR	10	0	1193,6265
MTHEVHTSQK	10	0	1196,5608
TMHENVPKNQ	10	0	1196,5608
KAWLPVAFPGL	11	0	1197,6909
NDGVLAVNLGMP	12	0	1198,6016
LNDAMRLVGPN	11	0	1198,6128
ELAAC(+57,02)RLVGPN	11	0	1198,6128
SSELQGVLVGPN	12	0	1198,6194
SN(+,98)TLGARLVGPN	12	0	1198,6306
ESSLQRLVGPN	11	0	1198,6306
HNVSGGADSSNR	12	0	1199,5278
NNDELKLLQN	10	0	1199,6145
NMVPEVKLATV	11	0	1199,6584
KGSSFKLPAAVP	12	0	1200,6865
PGPEDQTHAHN	11	0	1201,5112
DGGSLRPDEGSL	12	0	1201,5574
Q(+,98)ENTLVTGALVG	12	0	1201,6189
ADVFTHTADVK	11	0	1202,593
RAPGVATPAHVK	12	0	1202,6882
LHGTAAVGPTALP	13	0	1203,6611
LHVTRPNPLAS	11	0	1203,6724
LHVTRPSNALP	11	0	1203,6724
DALARPM(+15,99)PM(+15,99)GD	11	0	1204,5217
EETYGTVLKAP	11	0	1206,6133
LLDQ(+,98)LGEGVHQ	11	0	1208,6038
FGNPPPNGEPGQ	12	0	1209,5415
QGDPAGKWSLVG	12	0	1213,6091
MEHKLEGFLN	10	0	1216,5911
MLPPPHDANKV	11	0	1217,6226
NGPEN(+,98)EFVPSE	11	0	1218,5039
GGGPQ(+,98)DEFAGNGL	13	0	1218,5151
NGPEN(+,98)EYKPGN	11	0	1218,5151
GNPQ(+,98)DEFLGNQ	11	0	1218,5151
GNPQ(+,98)DEFNVAQ	11	0	1218,5151
NGPQ(+,98)DEFLQGN	11	0	1218,5151
GNPQ(+,98)DEYNGPK	11	0	1218,5151
GGGPQ(+,98)DEFNVAQ	12	0	1218,5151
GNPEN(+,98)EFVGQQ	11	0	1218,5154
GNPEN(+,98)Q(+,98)FANVQ	11	0	1218,5154

NGPQ(+,98)DEFRQQ	10	0	1218,5264
GNPEN(+,98)EFRAGQ	11	0	1218,5264
GNPEDEFQQR	10	0	1218,5264
GNPENTNMSKQ	11	0	1218,5298
TAPEDESGSAKE	12	0	1219,5205
TAPEN(+,98)EFDLPS	11	0	1219,5244
ATPQ(+,98)DEFALGGN	12	0	1219,5356
KASGNYGGTN(+,98)PGP	13	0	1219,5469
TAPKPHTGAPML	12	0	1219,6382
AVGVLPHSVNLD	12	0	1219,656
FVGGVSKSVNLN	12	0	1219,656
LTSGEKVYVPK	11	0	1219,6812
RHAGLPTSKGPT	12	0	1220,6626
PGGPAHGGAGDSSR	14	0	1221,5486
DGDSLWNLVGF	11	0	1221,5667
FM(+15,99)KGMFFDGQ	10	0	1222,5151
M(+15,99)GDFPGSQNAGQ	12	0	1223,4878
FGDFVGPGEDGQ	12	0	1223,5095
M(+15,99)GDFVSAAEPGQ	12	0	1223,5129
M(+15,99)GDFVSGTNPPS	12	0	1223,5129
M(+15,99)GDM(+15,99)VQVDAVQ	11	0	1223,5161
FGDGTGSHFDGK	12	0	1223,5208
MGLEQNSFNGE	11	0	1224,5081
SFLNN(+,98)LFEKL	10	0	1224,6389
LLVN(+,98)VSPLKML	11	0	1226,7307
DGQGAGLAKLAGNG	14	0	1227,6206
KALAETAAVQVE	12	0	1228,6663
FGLQGATVFQY	11	0	1229,6082
NNSKNVQQLW	10	0	1229,6152
ELLASQGTKEVG	12	0	1230,6455
PKMANMM(+15,99)LAPL	11	0	1231,6128
WLPGSKVSVME	11	0	1231,627
QLSENPGYKAQ	11	0	1233,5989
KDDVVGNAASMK	12	0	1233,6023
LADPTLSQYVK	11	0	1233,6604
VVVGLHGSSVALP	13	0	1233,708
KVKDGGPAHVVK	12	0	1233,7192
GNQQ(+,98)N(+,98)AGAGLFY	12	0	1240,5359
DGDSLWVQPPE	11	0	1241,5564
NGHVPFKYSPP	11	0	1241,6191
KGEDQKSVKLD	11	0	1245,6565
ELGAGTTSGMLVL	13	0	1247,6431
QGGPLAVTKGNPN	13	0	1251,6572
VAVSVVMNPQNP	12	0	1253,6438
YVVFVVMRKD	10	0	1254,6794
MMVFVTGKSKK	11	0	1254,6829
VFPPTPRVVLM	11	0	1254,7158
VFPAQPRVVLM	11	0	1255,7112
PAFGSPVSVFLH	12	0	1256,6553

VFPPTPRVTLM	11	0	1256,6951
LM(+15,99)PGQM(+15,99)QTGTY	11	0	1257,5369
TM(+15,99)LVGAVVELNP	12	0	1257,6638
NVETKLLVSQQ	11	0	1257,6929
LTEWRLVETL	10	0	1258,6921
SGSLM(+15,99)HPQSTSL	12	0	1259,5815
MQANGM(+15,99)NGPAKK	12	0	1261,5908
YPPGPFTAGGDLA	13	0	1261,5979
LDAGEPDLQYGS	12	0	1263,5618
FVEGGQPRYLV	11	0	1263,6611
KAWLPSTHVKV	11	0	1264,7292
YNFLPPFLKK	10	0	1265,7173
EGTSLWNLEPK	11	0	1272,635
MYPVDFENLF	10	0	1273,5688
FMEGMKLPHGK	11	0	1273,6311
VDFVPEVGQVW	11	0	1273,6343
FVKGGGSPNGGAGGL	15	0	1273,6414
PAELFVVFPNF	11	0	1278,6648
LLDTSTQLYVK	11	0	1279,7024
NMVNSDMTQGGQ	12	0	1280,5125
YPEEEC(+57,02)EVVQ	10	0	1280,5229
NEEWFFKAPN	10	0	1280,5825
LTLVVNRAAEPT	12	0	1282,7244
EQEEHMANQAT	11	0	1286,5198
VKESN(+,98)PVSSNLL	12	0	1286,6719
NGKSAMTKTPRP	12	0	1286,6765
RGLGPKPNTPPR	12	0	1288,7363
VLLEGPSYTALQ	12	0	1289,6868
ESKGPPTMWLM(+15,99)	11	0	1291,594
GVGVLLGM(+15,99)SSVM(+15,99)L	13	0	1293,6672
LGELDLFDSTGE	12	0	1294,5928
KMTPVNAQ(+,98)LSPN	12	0	1299,6492
LTAVQSQNDVLL	12	0	1299,7034
ETNVVTMPLALL	12	0	1299,7107
LC(+57,02)SALGPAFPGVD	13	0	1302,6277
MSWTAPPLNASM	12	0	1304,5894
LTLGLMFSGSPAL	13	0	1305,7002
FMPVDTPPAVSF	12	0	1306,6267
QENDVRPHANE	11	0	1307,5854
RSASERRGSVAH	12	0	1311,6755
VLPGFTNSGVPLL	13	0	1312,739
AVENFNGPNGKGL	13	0	1315,6521
RFVLNVKVEGR	11	0	1315,7725
KTSPGGTPTHLSH	13	0	1318,6628
VTLGEAPGSPPAPE	14	0	1320,656
LQLDGAAGSYLDV	13	0	1320,656
NQLDFFPALGSN	12	0	1321,6301
LVSGGM(+15,99)VASVYSH	13	0	1321,6335
NQLGAHGSPALSGN	14	0	1321,6375

SLVNAGDLHLM(+15,99)H	12	0	1321,6448
NQLDTGYSAAAVL	13	0	1321,6514
LQ(+,98)LDTGGGGFLML	13	0	1321,6587
NQLDTGPHAGVTL	13	0	1321,6626
NQLDVAGHPTGTL	13	0	1321,6626
NQLDPGGAGQGPVL	14	0	1321,6626
NQLDVNPGLQAPG	13	0	1321,6626
NQLN(+,98)SVPGVKHE	12	0	1321,6626
WGAVGVVSPPGAPE	14	0	1321,6665
TLLAGSAPGALALAP	15	0	1321,7605
TLLSASGPPKALAP	14	0	1321,7605
WLAKKVSPPTVP	12	0	1321,7759
WLALLPAAAQRL	12	0	1321,7871
DKLDNYNSLLE	11	0	1322,6353
FPAHGATPNGLNQ	13	0	1322,6367
DKLDNKSYGGLN	12	0	1322,6465
DGSVVAFAM(+15,99)GGALL	14	0	1322,6541
M(+15,99)PAVKHSSPLGSP	13	0	1322,6653
EGLTKPDLPLLK	12	0	1322,781
NSVGGGGDTTYAEP	14	0	1323,5579
EDAVGVVRVHPM(+15,99)	12	0	1323,6604
DKLDNYSPLLF	11	0	1323,6709
FLLN(+,98)NYSDLKP	11	0	1323,6711
VAFPPTPGVVVLM	13	0	1325,7417
RTSNVLTDPLVL	12	0	1326,7507
FLPAPVPDVYLT	12	0	1330,7173
LGSLSSTEKPGASV	14	0	1331,6931
PTQSEGKRVPHP	12	0	1331,6946
PTESDAKPVVGHP	13	0	1332,6672
AVEMDKPFVANL	12	0	1332,6748
AVC(+57,02)AVPGAAYELL	13	0	1332,6748
AVEYVAKGSLEAP	13	0	1332,6924
KNGSGLGPPEPPAD	14	0	1334,6465
YDSVKQAPAKGGD	13	0	1334,6465
PGGHAPGGGGTLMNN	15	0	1335,5989
KNGAAGPALSLLPQ	14	0	1335,751
NYFVSGEHAGNGS	13	0	1337,5637
EHGTVSDGLFNY	12	0	1337,5889
DWVSLGPVLPVAS	13	0	1338,7183
NSFLSNAHVHKS	12	0	1339,6633
ATANARYLFSVQ	12	0	1339,6885
YPPGGALAVAAEPK	14	0	1339,7136
FLPASGASVVAPPQ	14	0	1339,7136
EALGSEGAKLFGY	13	0	1340,6611
LGSGGGGLQ(+,98)ELLEN	14	0	1343,6567
SGNLFGDKGNKHA	13	0	1343,6582
VDDVLKVSGVSNL	13	0	1343,7295
LTDVFVASPPVSL	13	0	1343,7336
DLAYGLGPNAGNGQ	14	0	1345,6262

NALSQ(+,98)ADSASGGAVV	15	0	1346,6313
PSAEWLDNFLAS	12	0	1348,6299
TSGGALWNLDLGF	13	0	1349,6616
ATN(+,98)SLAN(+,98)NLRYL	12	0	1350,678
QASVLPAKDTPKP	13	0	1350,7507
PM(+15,99)EDAFFQAPLA	12	0	1351,6118
M(+15,99)VAPDADYGSSKP	13	0	1352,5918
VGGGEHGGGADGLHH	15	0	1355,5967
NNGPAALKHGEHL	13	0	1356,6897
SVVFVRPAPTER	12	0	1356,7512
LKSNSASLLTNPL	13	0	1356,7612
NAGAM(+15,99)KTPAASVVQ	14	0	1359,6816
SLQSVVGKPPSTY	13	0	1361,719
SLEEPPKGPLPGAA	14	0	1361,719
SLSLAM(+15,99)NGGAKVKA	14	0	1361,7336
SLFFLLGQPAAGAA	14	0	1361,7344
HLVGPDKKRLLS	12	0	1361,8142
YVFVKMVPAVDP	12	0	1363,7209
HGKEPQQWSLR	11	0	1364,6948
KAAPAVEHVGPGSF	14	0	1365,7041
HGKDNQKEGLSR	12	0	1367,6904
VAQQATVVTTSTY	13	0	1367,6934
HGKN(+,98)DQKVSEVK	12	0	1368,6997
YGLALGKLFAMW	12	0	1368,7263
HDAGGVVVSKSEW	13	0	1369,6626
VSGWDVGVLGPDGL	14	0	1369,6877
EPN(+,98)LTKPPHLSH	12	0	1369,699
ALMAGTDVWYVM(+15,99)	12	0	1371,6204
QPAHPPGSPSGMPL	14	0	1371,6604
TNALFGSGAHRNQ	13	0	1371,6643
MLLGYMVLASFK	12	0	1371,7295
LSRTGLGSEQVLL	13	0	1371,7722
LMVVSVNKAWNL	12	0	1372,7537
SADVFLPQANKW	12	0	1374,6931
SADVFLRGPAVM(+15,99)P	13	0	1374,6965
WSVM(+15,99)LKLTNNGP	12	0	1374,6965
SGEVFLHDAAAFL	13	0	1375,6772
LSPM(+15,99)KDGLM(+15,99)AGNL	13	0	1377,6633
NMASTVHEHQPE	12	0	1378,5935
VNSMAVYGQSN(+,98)PL	13	0	1379,6392
HGKQNEQTGVALT	13	0	1381,6951
HGKQDQKVSVTVG	13	0	1381,7314
HGKEDQKANATGK	13	0	1382,6902
HGKAAPLSGNKAPH	14	0	1383,7371
M(+15,99)LVPEVAAGTVVSP	14	0	1384,7273
LATAAGSGGVAVSVVE	16	0	1386,7354
FGVVQNLTTALQP	13	0	1386,7507
WGLANLFGTALEP	13	0	1387,7136
PM(+15,99)LALLYQGNPGV	13	0	1387,717

GFVVLDKSNALEP	13	0	1387,7346
RADAHARTPLLPA	13	0	1387,7683
MLNQKTKNVSLN	12	0	1388,7446
REEATHAGPGLPR	13	0	1389,7112
ASSKEKFQDKLL	12	0	1392,7612
TKGVSHERVGPKV	13	0	1392,7837
YMMLVGPLDVAW	12	0	1393,6775
PNKLTLKDAGAAVP	14	0	1393,793
ERASEGMVLLMY	12	0	1397,6682
HGFHGGLTKAAAPH	14	0	1399,7109
DVKESDLTASGPLA	14	0	1401,6987
VDSSVVQAAGDGLGK	15	0	1401,71
LM(+15,99)GPYGPQPAPM(+15,99)L	13	0	1402,6624
YPGLN(+,98)SVGAPAPM(+15,99)L	14	0	1402,6802
SKQESDNGQGLKL	13	0	1402,7051
SKKQ(+,98)SNAVTDVLN	13	0	1403,7256
KVM(+15,99)PSGGSSPAFPE	14	0	1405,6548
FYEVKMATEYK	11	0	1407,6743
AKELGHM(+15,99)GGAYKH	13	0	1413,6824
VPAVRLPHEGNLN	13	0	1414,7681
MNADLSVLSDASPP	14	0	1415,6602
NMSLVSVGPTGDGAL	15	0	1416,6919
LEVLWAM(+15,99)NGGKAD	13	0	1418,6863
NYC(+57,02)NARYALLY	11	0	1419,6604
DHGKLSRNLNGPL	13	0	1419,7583
YDVDGPPLVFDGQ	13	0	1420,6511
YDWHVRVM(+15,99)DGK	11	0	1420,6558
RHLVHPGTVATAY	13	0	1420,7576
NVNHTFLMFDGQ	12	0	1421,6399
FGNFMLFASKM(+15,99)N	12	0	1421,6472
M(+15,99)GLM(+15,99)PPLPSSTM(+15,99)L	13	0	1421,6604
EGHAAMNLAHLFN	13	0	1423,6667
VGHPGSKSTLADNAA	15	0	1423,7056
KALTDNLFPGSVY	13	0	1423,7346
FLVGPLAHAGTELT	14	0	1424,7664
TAVFPPTPGVVVLM	14	0	1426,7893
ATVFPPTPRVVLM	13	0	1426,8005
QAVSTATC(+57,02)DLTVY	13	0	1427,6602
PESGVTTLGFVPPK	14	0	1427,7659
MQDLADNLGLPML	13	0	1429,6946
FVVLGHEMGGWSL	13	0	1430,7017
MTDKAGNSVDGGPLA	15	0	1431,6663
MADFVSVSSGHSPN	14	0	1433,6245
KEGAGPMLSVFN(+,98)GQ	14	0	1434,6814
FGDFVSPLAPSGEN	14	0	1435,6619
FGDFLVYEFATQ	12	0	1435,666
FGDFVSSARPPEQ	13	0	1435,6731
FGDM(+15,99)VSVLPRDGQ	13	0	1435,6765
FGDFPGGPPVFDGK	14	0	1435,6772

FGDFVLVAGN(+,98)PATQ	14	0	1435,6982
FGDFVSTEVSHVL	13	0	1435,6982
VAPLLKAEQAVAVQ	14	0	1435,8398
KTLTSPAPVEVLAN	14	0	1438,8032
TFLLAASFPMTSGV	14	0	1440,7322
WLLLGNPELLKF	12	0	1441,8333
MPVKGSDAARADLL	14	0	1442,7551
EAQGNKTLKGHVY	13	0	1443,7471
EGLYNNKEGPLVL	13	0	1444,7561
LPTQSVSQLYPGR	13	0	1444,7673
WLYNNPGAVSLVL	13	0	1444,7715
YNTYSFVGVHNM(+15,99)	12	0	1446,6238
FDVPNGFEYVNF	12	0	1446,6455
FPAQEGFEYVNF	12	0	1446,6455
M(+15,99)KLPGPNGEPAPHS	14	0	1446,6926
NNAVFKGATGQDLL	14	0	1446,7466
YN(+,98)TYEGAVLRDM(+15,99)	12	0	1447,6289
FMQGNNTLYVDF	12	0	1447,6443
NSVPTGLHGGPADVK	15	0	1447,7419
MKTGKVSDPFGPGK	14	0	1447,7493
GMLYNLLLVLSVN	13	0	1447,8108
LPAEFPGDGGKAAGY	15	0	1448,6936
YDTVGQAADKAWE	13	0	1452,6521
VHTGHPLRVAHTE	13	0	1452,7585
NVKAAAVAEPLAYH	14	0	1452,7725
VAAFPKAC(+57,02)GGLTHE	14	0	1456,7131
C(+57,02)LVTLKEPLSLW	12	0	1457,7952
LSVMGKKKNTQPE	13	0	1458,7864
HSANRGDLPNTAHA	14	0	1459,6917
LQ(+,98)DAGLVKKGNGFL	14	0	1459,8035
LEDAKRLQGRFK	12	0	1459,8259
LAPDGAQLEALLGPV	15	0	1462,803
C(+57,02)ALLHDAALMGHR	13	0	1463,7126
HGPLRSQLSNSLR	13	0	1463,7957
HGKEPGAVKWSLR	13	0	1463,7996
FLYTSTVRTNPPA	13	0	1465,7566
PKLGGGPAPKAPLGHA	16	0	1466,8357
EVLSQGTVSNNPPK	14	0	1468,7522
TPLGEGHGKAKVLY	14	0	1468,8037
NDLVLGGPHWHLN	13	0	1470,7368
LLTVYGVLGPAKEL	14	0	1471,865
FPNVMVVAPDSVSL	14	0	1473,7537
MFPLKLPAM(+15,99)SVPK	13	0	1473,8088
GFNFVSSLPEPPGQ	14	0	1474,7092
FAATHGPTSNPHNK	14	0	1477,7063
NLPAYSNKALVKY	13	0	1479,8086
VVN(+,98)GGFFDDFLPR	13	0	1482,7144
KADNFFNTSGLTVA	14	0	1483,7307
WNLLTLTGPSASPK	14	0	1483,8035

RTVGGNGPQKLDFP	14	0	1484,7734
TAAPPSLAM(+15,99)SNANNL	15	0	1486,7085
LSVSSTSVPDRLLN	14	0	1486,7991
LNNSPDSPPAGPPPE	15	0	1487,6892
LNNPSSDNNPAAFK	14	0	1487,7004
YMPHGVQAFFVGH	13	0	1488,6973
LEFGLTTDGPASPNA	15	0	1488,7095
M(+15,99)VLYNVKNGPLPE	13	0	1488,7646
AALLEVHNALAAVGAA	16	0	1489,8252
NDNGQTDN(+,98)PALATC(+57,02)	14	0	1490,5942
LSAAPSDTTNFLAAL	15	0	1490,7617
QSAATAGGLHNGLGNQ	16	0	1494,7175
GLKHWAQNTAKEL	13	0	1494,7942
NFVAPVKGSAGGNALP	16	0	1497,7939
VDGRGNVATSADVLQ	15	0	1500,7532
VDC(+57,02)LWNLDTGVVL	13	0	1502,7439
MNLALGNFTLALPE	14	0	1502,7803
YPHADTSEGNPGLM(+15,99)	14	0	1503,6299
ENTHNKRC(+57,02)WLF	11	0	1503,7041
KTFANM(+15,99)GM(+15,99)QSVFL	13	0	1504,7053
YVPM(+15,99)KGVFSHLLV	13	0	1504,8113
EDGLLALN(+,98)HGVGVPD	15	0	1505,7361
MLGKVVYWDATAGP	14	0	1506,7542
LLSGNYDGLAPKYP	14	0	1506,7717
M(+15,99)DAASLAFLESLPE	14	0	1508,7068
LADPLAGSEPGANGKL	16	0	1508,7834
AYFVLLNYGNPKN	13	0	1511,7771
FLPAVNTGTASVKHA	15	0	1511,8096
RWVFVPEPSKGLV	13	0	1512,8452
FSAVTPVLVTASHSV	15	0	1513,814
NKTLESGDSKKVPN	14	0	1515,7893
LVATNYGNWPRLL	13	0	1515,8198
VATGGGRRRTFLPE	14	0	1515,8269
RVSLEDAKLKGVFG	14	0	1517,8564
RVAAQSSAVGKLFKG	15	0	1517,8677
MPVAGLSLGTKWME	14	0	1518,7573
MLVSGLFSEGGVPVK	15	0	1518,8115
VQLLDTGPKANHVK	14	0	1518,8518
LLAVPSYSNAPKNF	14	0	1519,8035
ENKNTVVVYSGSLD	14	0	1523,7468
MDFMGFQ(+,98)LGFVED	13	0	1535,6311
EHDLALEFGYDDL	13	0	1535,678
FVN(+,98)YGFEFGNQTL	13	0	1535,6931
C(+57,02)SDYGLLFGFMNN	13	0	1536,6377
MYTWFLLGYDDN	12	0	1536,6594
FVDAVFEM(+15,99)GYDKT	13	0	1536,6807
NMASTSFNGLPVQGD	15	0	1536,6877
AVPDASPAAHMPTLY	15	0	1539,739
LGEENLRALLADNL	14	0	1539,8257

ARARVGGGKAATAAKR	16	0	1539,907
GKLLTVGDTATPALKG	16	0	1540,8823
VALNPSNSEGASHLF	15	0	1541,7473
TPKEGSKFAGPTLLT	15	0	1545,8401
QEAHLGFGTGSALPY	15	0	1546,7415
MFNAGGGGLESPGQAR	16	0	1547,7151
GSRGQGAEQHQKVLG	15	0	1550,7913
LVSVNGNELAFELF	14	0	1550,7981
GSVLEQEAGHQKGLV	15	0	1550,8052
GSGVGQKEAGHQKGLV	16	0	1550,8164
GSGLVGAGSGAGHQKVLG	18	0	1550,8164
WGTLEGAAPKSDVLD	15	0	1557,7673
MLVSGGPGRKNYLPA	15	0	1558,8289
VGRLGENDYPVLTK	14	0	1559,8308
YNSVMLMGFGWFL	13	0	1563,7253
SSPPPTGGSNVWLEH	15	0	1563,7317
DLEGAKSLAPPDPM(+15,99)L	15	0	1568,7756
MAPMGWGLGAVFGAHA	16	0	1571,7378
LLTSHGFFMTKPAQ	14	0	1576,8071
YDYDLM(+15,99)YLGMSGH	13	0	1579,6323
EVAPPGGPFLTGDLPN	16	0	1579,7881
GSTAMKQAVDLHGGPN	16	0	1581,7568
NEPAVGVLPHADHNL	15	0	1581,7898
KTAHTQM(+15,99)SAGLTTPK	15	0	1586,8086
LQ(+,98)DAKRAAVFLDQL	14	0	1587,8621
LQ(+,98)DLGGGALKSGNVFL	16	0	1588,8459
SATQGHGPEFGYDDL	15	0	1592,6743
FM(+15,99)EVGREFGYDN(+,98)L	13	0	1592,6816
LTM(+15,99)MSREFGYN(+,98)N(+,98)L	13	0	1592,6851
PTYSSSLQ(+,98)FGYDDL	14	0	1592,6882
MVMYGFELTAGDYP	14	0	1592,689
LNEAGGPPEFGYDDL	15	0	1592,6995
VFNDFLEFGYDDL	13	0	1592,7034
PTDENGNSVFGKTNN	15	0	1592,7065
ETMYGFEVYLNDL	13	0	1592,7068
VVDENGDKGFDGAGTL	16	0	1592,7317
VVDENGNSVM(+15,99)VSSQL	15	0	1592,7351
VVYYGFSSASATNDL	15	0	1592,7358
ATQ(+,98)PTGGSNVFN(+,98)TARA	16	0	1592,7429
PTPM(+15,99)NGNSVFTVSNL	15	0	1592,7505
KADENGNSVPGYDKT	15	0	1593,7271
VTPM(+15,99)NGNEGHVEQAP	15	0	1594,7046
FTDYGFEYLVMPD	13	0	1595,6853
LQNLTVPPLALATTF	15	0	1597,908
PSLYMSGAGYPAQMK	15	0	1599,7424
DGVKEGVPVKC(+57,02)KVEG	15	0	1599,8291
KVPENGNSPAGPANRP	16	0	1603,8066
TPQFGSSFKGNAGYF	15	0	1606,7415
NEEEGFFPGLGREQ	14	0	1607,7214

VNDENGNSVLESGKF	15	0	1607,7427
EAGDFLHVHSSALMP	15	0	1609,7559
KDTPGVAGTDELGDKN	16	0	1615,7688
NLFGLSATPRVENHA	15	0	1624,832
YLKEPTASLNPKRL	14	0	1628,925
GFHVALTLSGAGGGEPY	17	0	1631,7944
SVLPTESEGASSTLAAL	17	0	1631,8254
WLPTESWKPVVQY	13	0	1631,8347
WTSLVSNLSDSTVPK	15	0	1632,8359
PM(+15,99)GLPLLLDNKSPLL	15	0	1635,927
FEPQRNLNSSWME	13	0	1636,7305
KMANGPTRGEMWLM(+15,99)	14	0	1636,7524
FMWMLEARTPPTGT	14	0	1636,7742
C(+57,02)ALSYDLSGPAGPANF	16	0	1638,7349
PEAAVLTM(+15,99)TPGANNLQ	16	0	1641,8032
DYLLGEDFPEGM(+15,99)PF	14	0	1644,7017
MLYPLAHMFLGLQN	14	0	1646,8313
LLPAYSNSKPLLYGL	15	0	1647,9236
FVSGM(+15,99)VGQTGANDGVVP	17	0	1649,772
NKLDFVESEASSKVT	15	0	1652,8257
GTSPMASAFAASKQGED	17	0	1653,7305
LTNKYNLPMAYAKK	14	0	1653,8911
MPSDGVSNLNVTPLLV	16	0	1654,8601
YM(+15,99)AAGPGGTVGGDGGGVGY	19	0	1657,7041
YDATFTDGNVLVTTF	15	0	1662,7776
GVGSVVMEDQHQKLH	15	0	1662,8147
EQLPTLFVTTGSPLAP	16	0	1669,8926
FVLANPSSSKLGNDPK	16	0	1672,8784
WLYNDKDAPLVLVQ	14	0	1672,8823
MNAHAGFELKGSAVDE	16	0	1674,7671
KPAVEGGGPAGNGGFVFD	18	0	1674,8
PTLLPAYMALSLLHH	15	0	1675,9119
MFWEHKWGVSAATQ	14	0	1676,7769
QGNFLFM(+15,99)YQYTNF	13	0	1687,7341
M(+15,99)GLGGWLGM(+15,99)PLDADPQ	16	0	1688,7539
WVDTTLVSSASHKLF	15	0	1689,8726
EKLFMPGVSSLVNLY	15	0	1695,8906
AWLFMPGVPGPAGLRQ	16	0	1695,8918
M(+15,99)KNHAMM(+15,99)ALQVNN(+,98)Y	14	0	1696,7371
AWLPTESWKVPRAY	14	0	1702,8831
AWLPHVGTLRGTSHAT	16	0	1702,8904
RTLPTEGGYRSASPLV	16	0	1702,9001
LSPYSGMLQ(+,98)FGYDDL	15	0	1705,7544
MEGLGGVVENVALC(+57,02)M(+15,99)N	16	0	1707,7629
HHFFLMFLMPDHH	13	0	1707,7803
LADRAHSSSFVAAGLAH	17	0	1708,8645
TVTVPAGEKVKGALLVQ	17	0	1709,0088
KDQLEEQEFHGVLGS	15	0	1714,8162
KTGVLYVEGTTNRGKV	16	0	1720,9473

QKSLGGDGASHQVLPTQ	17	0	1721,8696
FLTLTSKQAPSLGFLT	16	0	1722,9556
KVAGWLTFEVMSVFN	15	0	1726,8752
LQETVKTLEFGHMVP	15	0	1727,8916
GSRMLLPAGVFEFATAA	17	0	1736,8918
LFNMSPSDSDLFNPR	15	0	1738,7983
PLLHANMDSAPNHHVS	16	0	1738,8208
VFDYGMGLQ(+,98)MFGMML	15	0	1739,7432
PSSPLPTYVNGELMLK	16	0	1744,907
RGKGQHKM(+15,99)QHQKLR	14	0	1746,9536
KLASAHGTQALGQDNLK	17	0	1750,9326
QGDEGLLLM(+15,99)GEPYHGK	16	0	1758,8247
NAGNGSVLLM(+15,99)GEPYHGK	17	0	1758,8359
NAGNGSVLLM(+15,99)WPYHGK	16	0	1758,8511
MLSPSGGFEYVMLSLM	16	0	1760,8186
QLLVGPVAENAAYPVVK	17	0	1766,9929
SAEKAVLALTSSASVTPH	18	0	1767,9365
LAPGSTVFVSAASPPDSPA	19	0	1769,8835
VEVSQGLVSNSNPTLNL	17	0	1769,916
KVELAPHLGAGGPKTLSV	18	0	1773,0149
QAWLPTLWPAAVFC(+57,02)D	15	0	1773,8547
FFQMTMGPAEEAQVGH	16	0	1778,7756
HRETMADGANGEAQRH	16	0	1778,7866
APNGLPTQSVSKGLVSMV	18	0	1783,9502
DDDLHLVGSLEWATTN	16	0	1784,8218
QSKAGPDVFLLGAMLAAP	18	0	1784,9495
MLYPWMLLQGGPSADL	16	0	1790,8735
LFHKC(+57,02)GGGEKHKKPAT	16	0	1793,9358
SRGSRDGGVNQHQKLR	16	0	1793,9358
ERTASQMQAHM(+15,99)AQRH	15	0	1796,8159
HRQHNLNEEEQAGVH	15	0	1796,8303
YYKVVLDVKESDARQ	15	0	1811,9417
ELHTPTKSADKPVRAY	16	0	1811,9529
AEAPLPTQSWKPVRAY	16	0	1812,9521
LDANGAGGYVQTTTGAPEP	19	0	1817,843
LSEGDVFFVPRGPRED	16	0	1818,8899
AALFTKHGGKYVAAGVVM	18	0	1818,9814
WLADPGLAVFPGFHGKN	17	0	1824,9312
VVLN(+,98)VKGGGC(+57,02)GGSPWLLL	18	0	1825,9761
MFLLFVAHGPGSLLYY	16	0	1826,9429
KETVGQLFGPQGYELY	16	0	1827,9043
WRVLGGSFPADKGYLY	16	0	1827,9307
NLMSVSGLGPALFWMHA	17	0	1829,8958
KAWLPTQSDAKMSLGTP	17	0	1829,9346
QAWLPTQ(+,98)SWKVPRAY	15	0	1830,9417
DEGVLPGVLPDATGAYLM(+15,99)	18	0	1832,8865
KSLKHFAPTHHSEFVA	16	0	1834,9478
LADRTPSSPFVPEVRVA	17	0	1839,9841
KVVLDVKETTGSKLNGPG	18	0	1841,0259

SYFFSDVDKDNYQSK	15	0	1841,8108
DADLKKVGGSTVVEVGATP	19	0	1841,9734
RHHANGEHHQKVAKTP	16	0	1845,9458
VGDLPN(+,98)AN(+,98)SNGGQC(+57,02)AETM(+15,99)	18	0	1851,7251
NASGSTAMKQAVDVSNM(+15,99)K	18	0	1853,8611
MKFANAVVQQVYNHRG	16	0	1860,9417
C(+57,02)AVVAVAVAHSALPDVVGGA	20	0	1861,9719
TLFLPEVHSSFGTLLTT	17	0	1861,9824
FGVVLTNGFSDQLGPVVD	18	0	1862,9414
YMLALELPHGKLVAFY	16	0	1863,9956
ASRVLGPAAGNGAEAQKTAP	20	0	1864,9753
SLVVQESGAGEQHQKLR	17	0	1864,9756
YYKVVLDVKGDSAARDA	17	0	1868,9631
GLAWLPTKSDAKPVGFW	17	0	1871,9934
HDWAVFSQGVTGASEWL	17	0	1888,8743
FFLSHANADVGAVSGGVKK	19	0	1902,9951
QDVVGMPLMGNELGGEM(+15,99)L	18	0	1904,8682
YMLPTGNEYVATNAAFY	17	0	1923,8713
RPM(+15,99)LTFGSGGPGAVPLRPT	19	0	1926,0144
DSLLGLQASHPDLNVVGGAS	20	0	1948,9854
SARTKSHAGADVAAQVEPAS	20	0	1951,9712
SSLKQHKSSQSAGAASGGKH	20	0	1951,9824
LTTKQHQFC(+57,02)QHQKLR	15	0	1952,0164
NHQQTFSAGGVVPQAGTVY	19	0	1959,9438
C(+57,02)DVLEGNLYVNGPAGLLY	18	0	1965,9507
LLLGGAVLSMTQLAPSSSLL	20	0	1970,1121
LDVVVNYN(+,98)GQTVDDWQL	17	0	1977,9319
VDLVVMDGM(+15,99)MLVDDAENL	18	0	1993,9045
YNHAVQLMWPYMWLM(+15,99)	15	0	1997,8992
VVSSGKVDAGVLGTGVDQGGVV	22	0	1999,0586
LKEKLESLHKNGSHGQTT	18	0	2006,0544
QLSAEVVVGGVAPGRHVTHP	20	0	2009,0806
LTPLPAYSNSLLLLM(+15,99)KVQ	18	0	2016,1328
PAVEPAVSC(+57,02)MC(+57,02)C(+57,02)M(+15,99)VGVKN	18	0	2023,8657
SLGGASGGDSMKKAVFGTQGAE	22	0	2053,9739
TGGATLAHKEC(+57,02)EQHQKLR	18	0	2063,033
ETPETYYFNTVYTEYF	16	0	2065,8833
FMGSGPYSGLVSAVEWAQST	20	0	2072,9512
YATQEFATGQSGLEWAC(+57,02)R	18	0	2073,9214
LDAETM(+15,99)FSEGTADTVPETM(+15,99)	19	0	2075,855
KSSLKHQESDAGDHVSSLR	19	0	2080,0295
QSFVVHQGSASSGQHQKGLV	20	0	2080,0449
KSLPTGNQEGMEQHQKLR	18	0	2080,0483
QSSLRHVM(+15,99)TSEQHQKLR	17	0	2080,0596
QASSRPAKEGMEQHQKLR	18	0	2080,0596
KSAREHQSGGAYQHQKLR	18	0	2080,0674
PFLALSNLANAM(+15,99)AGAKLETH	20	0	2084,0723
YWEFVNVDHGTC(+57,02)AYTPE	17	0	2086,873
NSNNQLDKDSSGSLAEPQVS	20	0	2088,9558

NPNEVVLDVKGSHLAVPPPN	20	0	2095,1062
QATAQVSSASM(+15,99)SSVGAQ(+,98)LEVM	21	0	2097,9558
FLDMFVDFVPC(+57,02)HVMVEM	17	0	2114,9336
VVDEDGNSVMMRKQDLNVV	19	0	2147,0349
HSSQAKFEPLGSVGNEESEH	20	0	2167,9768
LLMWPYHGKENQKDALSR	18	0	2185,1101
AAPSVTNGENGKQEQHQKLR	20	0	2191,1094
EQSLLNAAM(+15,99)VAQQEHQKRL	19	0	2209,1272
NNNTDVAPGSVHDSHLYM(+15,99)M(+15,99)L	20	0	2245,9731
PFSAYLGGLTGHVNEFGYTNQ	21	0	2271,0596
KTALKGRHVSPAVGLVGSAVPDK	23	0	2286,3171
RGKTPSGFSFC(+57,02)GFSLGSANQKP	22	0	2329,1272
M(+15,99)LQPVVM(+15,99)SLPGPM(+15,99)SLGSNAGAKP	23	0	2329,1479
LLTKEDSMTLMDGLADNKPVHS	22	0	2414,1821
STPGSKEKEEKDDKKHKEHGAY	22	0	2527,2302
VVM(+15,99)DDTANQLNELLGSLAHGTGNTSL	26	0	2685,2915
